# Discovery and overproduction of novel highly bioactive pamamycins through transcriptional engineering of the biosynthetic gene cluster

**DOI:** 10.1186/s12934-023-02231-x

**Published:** 2023-11-14

**Authors:** Nikolas Eckert, Yuriy Rebets, Lilya Horbal, Josef Zapp, Jennifer Herrmann, Tobias Busche, Rolf Müller, Jörn Kalinowski, Andriy Luzhetskyy

**Affiliations:** 1 Department of Pharmacy, Pharmaceutical Biotechnology, Saarland University, Campus C2.3, 66123 Saarbrücken, Germany; 2 Department of Pharmacy, Pharmaceutical Biology, Saarland University, Campus C2.3, 66123 Saarbrücken, Germany; 3 Helmholtz Institute for Pharmaceutical Research Saarland (HIPS), Helmholtz Center for Infection Research (HZI), Campus E8.1, 66123 Saarbrücken, Germany; 4 Center for Biotechnology—CeBiTec, University of Bielefeld, Universitätsstraße 25, 33615 Bielefeld, Germany

**Keywords:** *Streptomyces*, Gene cluster, Transcriptional refactoring, Strain engineering, Antibiotic

## Abstract

**Background:**

Pamamycins are a family of highly bioactive macrodiolide polyketides produced by *Streptomyces alboniger* as a complex mixture of derivatives with molecular weights ranging from 579 to 705 Daltons. The large derivatives are produced as a minor fraction, which has prevented their isolation and thus studies of chemical and biological properties.

**Results:**

Herein, we describe the transcriptional engineering of the pamamycin biosynthetic gene cluster (*pam* BGC), which resulted in the shift in production profile toward high molecular weight derivatives. The *pam* BGC library was constructed by inserting randomized promoter sequences in front of key biosynthetic operons. The library was expressed in *Streptomyces albus* strain with improved resistance to pamamycins to overcome sensitivity-related host limitations. Clones with modified pamamycin profiles were selected and the properties of engineered *pam* BGC were studied in detail. The production level and composition of the mixture of pamamycins was found to depend on balance in expression of the corresponding biosynthetic genes. This approach enabled the isolation of known pamamycins and the discovery of three novel derivatives with molecular weights of 663 Da and higher. One of them, homopamamycin 677A, is the largest described representative of this family of natural products with an elucidated structure. The new pamamycin 663A shows extraordinary activity (IC50 2 nM) against hepatocyte cancer cells as well as strong activity (in the one-digit micromolar range) against a range of Gram-positive pathogenic bacteria.

**Conclusion:**

By employing transcriptional gene cluster refactoring, we not only enhanced the production of known pamamycins but also discovered novel derivatives exhibiting promising biological activities. This approach has the potential for broader application in various biosynthetic gene clusters, creating a sustainable supply and discovery platform for bioactive natural products.

**Supplementary Information:**

The online version contains supplementary material available at 10.1186/s12934-023-02231-x.

## Background

Natural products are a prime source of molecular entities with biological activities over the whole spectrum of therapeutically relevant indications, including anticancer, antibacterial, antifungal, antiparasitic, and antiviral activities. The structural and chemical diversity of natural products is enormous. These compounds have been evolutionarily optimized for “drug-like” properties, which can hardly be matched by any synthetic small molecules [20]. Natural products have been playing a key role in the infectious disease’s chemotherapy since the penicillin and tetracycline discovery which promoted the systematic screening for effective drug candidates from microbial resources. In around 8 years of modern chemotherapy, from the early 1940s to the beginning of the present decade, the share of natural products in small molecules for anticancer therapy is around 50%, and almost 70% of antibiotics are either natural products or directly derived therefrom [44]. However, the bacterial pathogens are also evolving under the antibiotics pressure by acquiring resistance mechanisms. The death toll of bacterial AMR (antimicrobial resistance) by some estimates was at the level of 4.95 million people in 2019 [2] and is rising exponentially. It is predicted to exceed 10 million deaths per year by 2050 [34]. A similar situation is observed in cancer treatment, where tumor resistance to chemotherapy is a major threat [4]. Clearly, the development of novel anti-infective and anticancer drugs is critical to stand a chance in the global fight against AMR infections and multidrug resistant cancer [27].

An almost untapped resource of novel bioactive metabolites is hidden in the genomic repertoire of Actinomycetes [38]. However, most natural product biosynthetic gene clusters (BGCs) are either silent under standard laboratory conditions or expressed at the level that produced compounds are accumulated at nanogram quantities, far below the amounts required for their exploration and exploitation [40]. Sustainable production of complex natural products in advanced chassis strains after heterologous expression of the corresponding BGCs is a promising solution to the existing situation [30, 38]. Moreover, the heterologous expression of known BGCs, responsible for the synthesis of high-value compounds, enables the generation of chemical diversity by design-based engineering of the encoded pathways [47]. On the other hand, the efficiency of BGCs expression in heterologous strains is rather low. A key reason for this is very likely the complex and often BGC-specific regulatory networks controlling the expression of BGCs in the native producer. Therefore, in order to achieve the sufficient production of the compound of interest, promoter refactoring of corresponding BGC by replacing native regulatory elements with synthetic ones could be considered as one of the most efficient solutions [19].

**Fig. 1:**
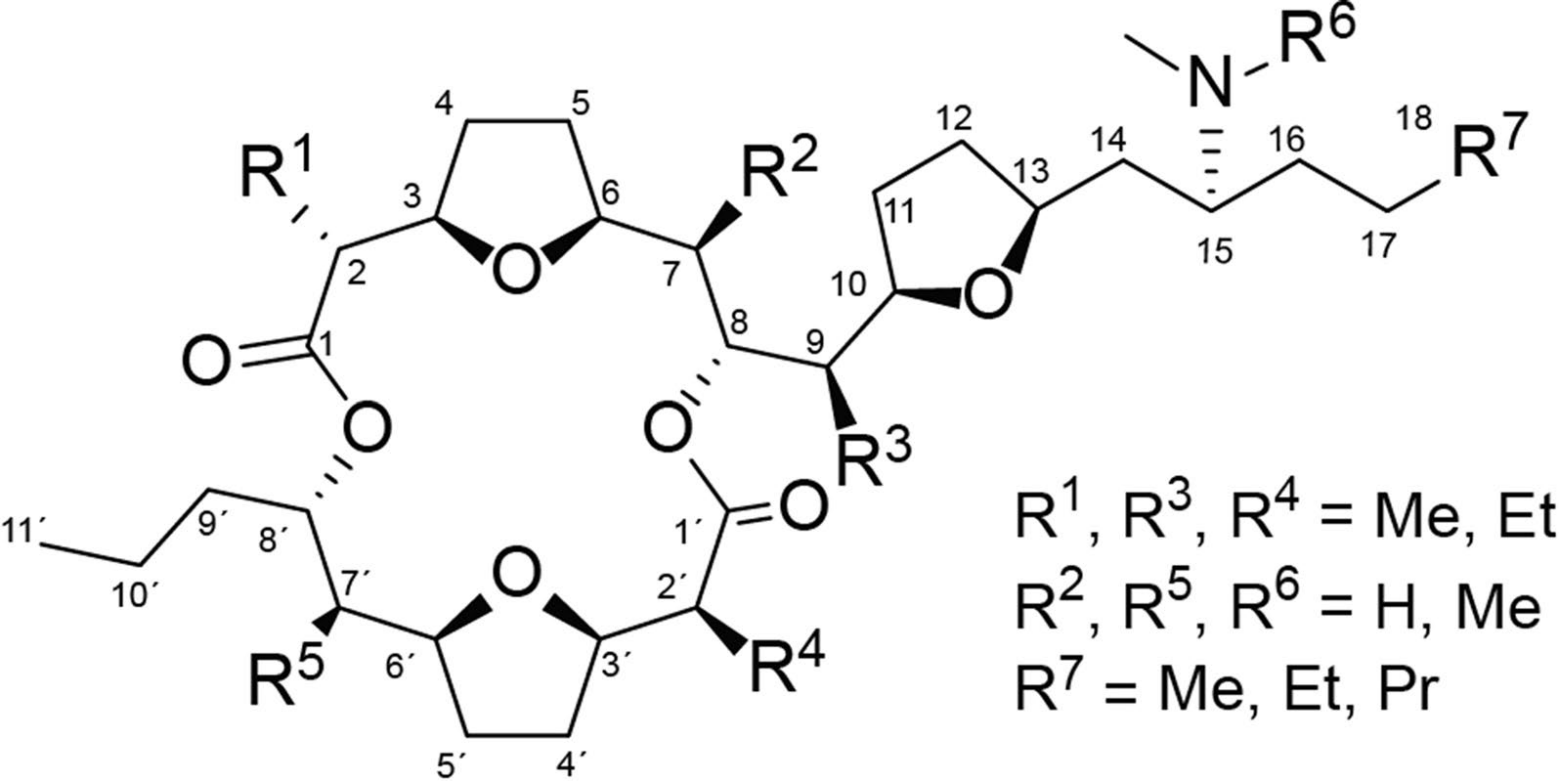
Chemical structures of pamamycin derivatives with side chain substitution patterns

Pamamycins are a group of unusual polyketides with molecular masses ranging from 579 to 705 Da, pronounced bioactivity and a challenging for chemical synthesis scaffold [15]. Pamamycins are produced by several *Streptomyces* species as a complex mixture of derivatives. However, all of them contain a 16-membered macrodiolide ring, formed by two hydroxy acids, commonly called as L (large, C1-C18) and S (small, C′1′- C-11′), with three cis-2,5-disubstituted tetrahydrofurans and adjacent alkyl-substituted chains (Fig. 1). The variations of these alkyl-substituents create a chemical diversity of pamamycins. At the same time, to date, only two pamamycins are known in which the hydroxy acid L (18C) is extended by one or two additional methylene groups: homopamamycin 621A (hydroxy acid L—19C) and bishomopamamycin 635 (hydroxy acid L—20C) [23]. “Light” pamamycins (such as pam-607, pam-621A, etc.) are major components of the produced mixture and thus could be relatively easily isolated from the fermentation broth. They show excellent activity against Gram-positive bacteria, including *Mycobacterium tuberculosis* clinical isolates within a narrow MIC range of 1.5–2.0 mg/L irrespective of their resistance to isoniazid or rifampicin [25]. Larger pamamycins with molecular mass of 649 Da and higher, were detected but never chemically or pharmaceutically characterized because of miserable yields [33]. Due to their extraordinary bioactivities and challenging molecular structure, pamamycins have stimulated intense synthetic as well as biotechnological efforts to provide sufficient amounts and enable pharmacological studies [9, 37]. However, chemical synthesis of pamamycins is very complex and protracted due to the stereospecificity of the tetrahydrofuran rings and the adjacent stereocenters of alkyl chains [21]. On the other hand, the biological production of these compounds, especially high-molecular-weight ones, is limited due to extremely low yields in native strains or heterologous hosts. A metabolic engineering approach was applied to improve access to high-molecular-weight derivatives, resulting in a better accumulation of some particular derivatives [13, 14]. Nonetheless, the problem of sustainable access to these compounds remains unsolved.

Herein, we report the modulation of the heterologous production of pamamycins through transcriptional refactoring of the corresponding BGC. The described approach is based on the construction and screening of a *pam* BGC library with sequence-randomized promoters controlling the transcription of key biosynthetic operons. Combining the engineered *pam* BGCs with the *S. albus* expression host with improved pamamycin resistance led to increased production and, as a result, the discovery and characterization of three novel highly active pamamycins: pam-635G, pam-663A and homopam-677A.

## Results

### Refactoring the transcriptional control of the pamamycin gene cluster by engineering promoters of key operons

Specialized metabolites biosynthesis in actinomycetes is mostly controlled at the transcriptional level. Therefore, the rational engineering of promoters within BGC of interest is an attractive approach to trigger or increase the production of the desired compound [29, 36]. The exchange of native promoters with well-studied natural or synthetic ones makes it possible to overcome regulatory barriers existing in the cell. However, this approach is mainly applied to individual biosynthetic or regulatory genes or operons rather than to the entire BGC due to the transcriptional complexity of the latter [19]. The efficient production of compounds seems to require the well-balanced production of respective enzymes, which is achieved by a well-orchestrated transcription of corresponding genes expression. Breaking this balance leads to the accumulation of side or shunt products, rather than the compound of interest [28].

**Fig. 2:**
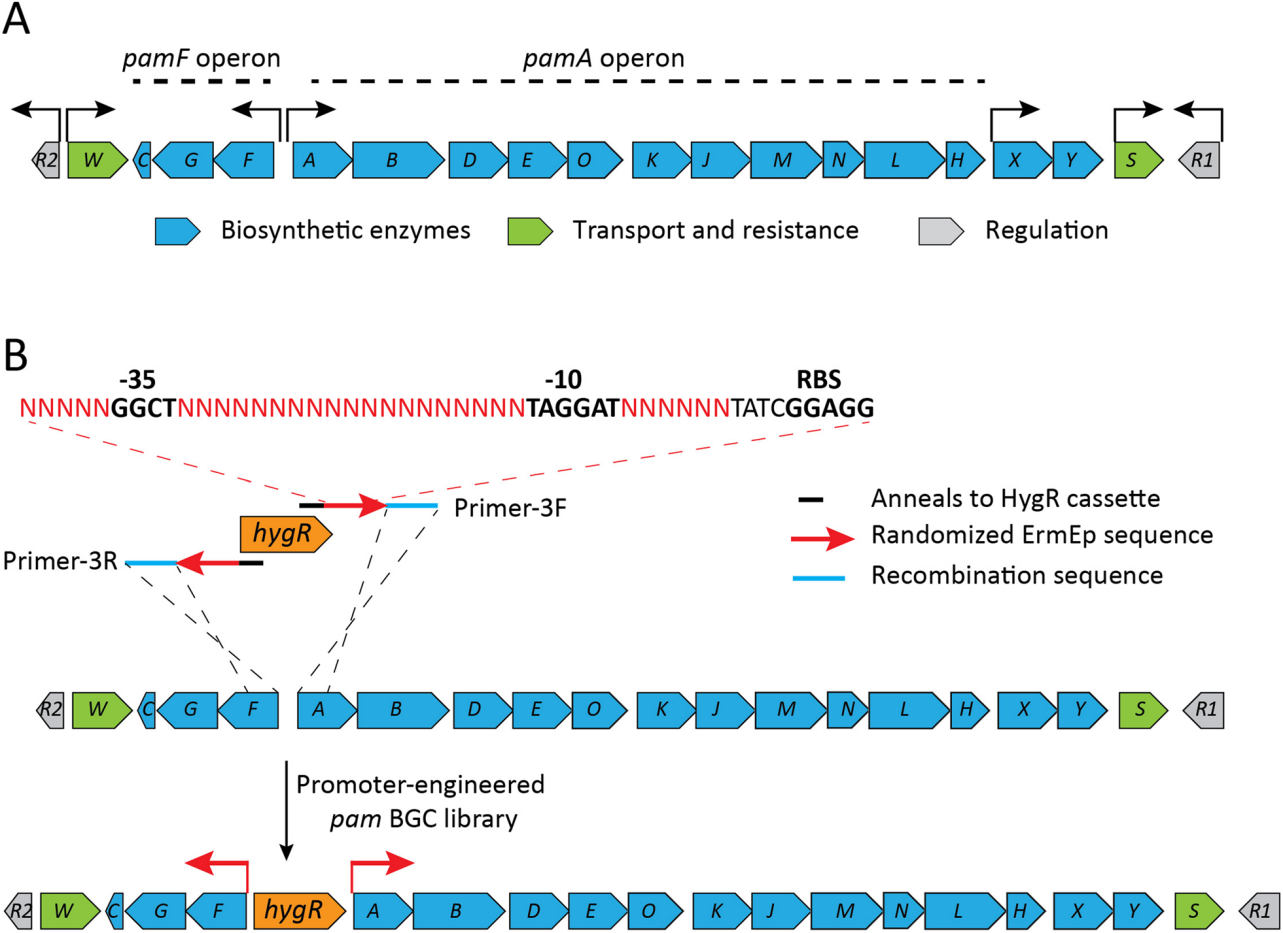
Schematic organization of the *pam* BGC and the applied promoter engineering method. **A** Schematic representation of genetic and transcriptional organization of the *pam* BGC. Arrows shows the location of promoters identified by RNA-seq experiments (unpublished data). **B** Construction of promoter-engineered *pam* BGC library. For details, please see main text

The *pam* BGC is organized into several transcriptional units, including three operons (RNA-seq unpublished data, Fig. 2A); two of them, *pamA,B,D,E,O,K,J,M,N,L,H* (*pamA* operon) and *pamF,G,C* (*pamF* operon), encode key biosynthetic enzymes involved in assembly of hydroxy acids S and L of which the final pamamycin is assembled (Fig. 1); bicistronic *pamX,Y* codes for aminotransferase and methyltransferase enzymes decorating the large chain [37, 39]. Other genes are expressed as single transcripts and encode regulatory and resistance functions. Such a simple transcriptional organization of the *pam* BGC allows one-step rational promoter engineering in order to increase the pamamycins production level.

With this aim, a library of pamamycin gene cluster variants carrying random synthetic promoters driving the expression of the two main operons was constructed (Fig. 2B). The promoters were engineered by preserving consensus − 10 and − 35 sequences and randomizing the spacer sequences of the erythromycin resistance gene promoter (*ermE*p1) from *Saccharopolyspora erythraea* [19]. Randomized promoters were inserted into the R2 cosmid carrying *pam* BGC between the *pamA* and *pamF* genes via Red/ET recombination. The insertion was facilitated by amplifying the hygromycin resistance gene with the primers carrying an aforementioned randomized sequence of *ermE*p1 promoter and RBS (Ribosome binding site). The primers also contain homology regions for recombination with the *pamA* and *pamF* coding sequences (see Materials and Methods section for details). The insertion of such cassette into R2 cosmid resulted in precise replacement of native *pamA* and *pamF* promoters with the random synthetic ones. The library consisting of approximately 5000 clones was constructed and stored as *E. coli* culture for further experiments.

### Construction of the expression host for the pamamycin BGC library

In our previous studies we came to the conclusion that the resistance of *S. albus* might be one of the bottlenecks affecting the level of pamamycin production in this heterologous host [37]. The *pam* BGC carries two genes, *pamS* and *pamW,* putatively involved in the self-resistance (Fig. 2A). The *pamS* encodes a putative pamamycin hydrolase, preventing the assembly of mature antibiotic prior to its export out of the cell, similar to how it is occurring in nonactin biosynthesis [6]. The *pamW* gene encodes a putative pamamycins exporter. To test whether these genes contribute to pamamycin resistance, *S. albus* strains carrying *pamS* or *pamW* genes cloned into a high copy number replicative vector under control of ermE*p promoter were constructed. These two strains, together with *S. albus* J1074 R2, with the chromosome integrated copy of entire *pam* BGC, were studied for their tolerance to pure pamamycin 607 by determining the MIC values in liquid culture (Table 1). As control, *S. albus* J1074 carrying empty vectors was used.

**Table 1:** MIC of pamamycin 607 for different *S. albus* J1074 strains

Strain	MIC, μg/mL
*S. albus* J1074 pUWLH	0.78
*S. albus* J1074 pUWLHpamS	1.56
*S. albus* J1074 pUWLHpamW	3.12
*S. albus* J1074 R2	12.5

*MIC* minimal inhibitory concentration, *R2* cosmid carrying the *pam* BGC, *pUWLH* replicative expression vector, *pamS* putative pamamycin hydrolase; *pamW*, putative pamamycin exporter

As a result, the strain carrying the entire *pam* BGC was found to be resistant up to 12.5 μg/mL of pamamycin 607 when compared to the empty vector control strain (Table 1). At the same time, strains expressing either *pamS* or *pamW* were tolerant up to 1.56 and 3.12 μg/mL of pamamycin. This makes us think that the products of both these genes contribute to pamamycin resistance but to different extents. Furthermore, to reach the maximum resistance the producing strain requires both proteins, since *S. albus* carrying the entire *pam* BGC demonstrated the highest MIC value. In fact, the presence of other than *pamS* and *pamW* resistance determinants within the cluster could not be ruled out. At the same time, the additional overexpression of *pamW* could contribute to better tolerance of the host strain to the produced pamamycins.

To construct a pamamycin resistant strain for expression of the recombinant *pam* BGC library, the *pamW* gene was introduced into the chromosome of *S. albus* J1074. For this purpose, *pamW* was cloned into the pTOS integrative vector [16] under control of the strong constitutive synthetic promoter P21 [45]. The resulting plasmid was then transferred into *S. albus* J1074, and the vector backbone was removed afterwards by expressing Dre recombinase. This resulted in the marker-free insertion of the *pamW* gene into the chromosome of the strain. The obtained strain, designated as *S. albus* J1074 P21pamW, was used as a host for expression and screening of the engineered *pam* BGC library.

### Functional screening of the recombinant pam BGC library

**Fig. 3:**
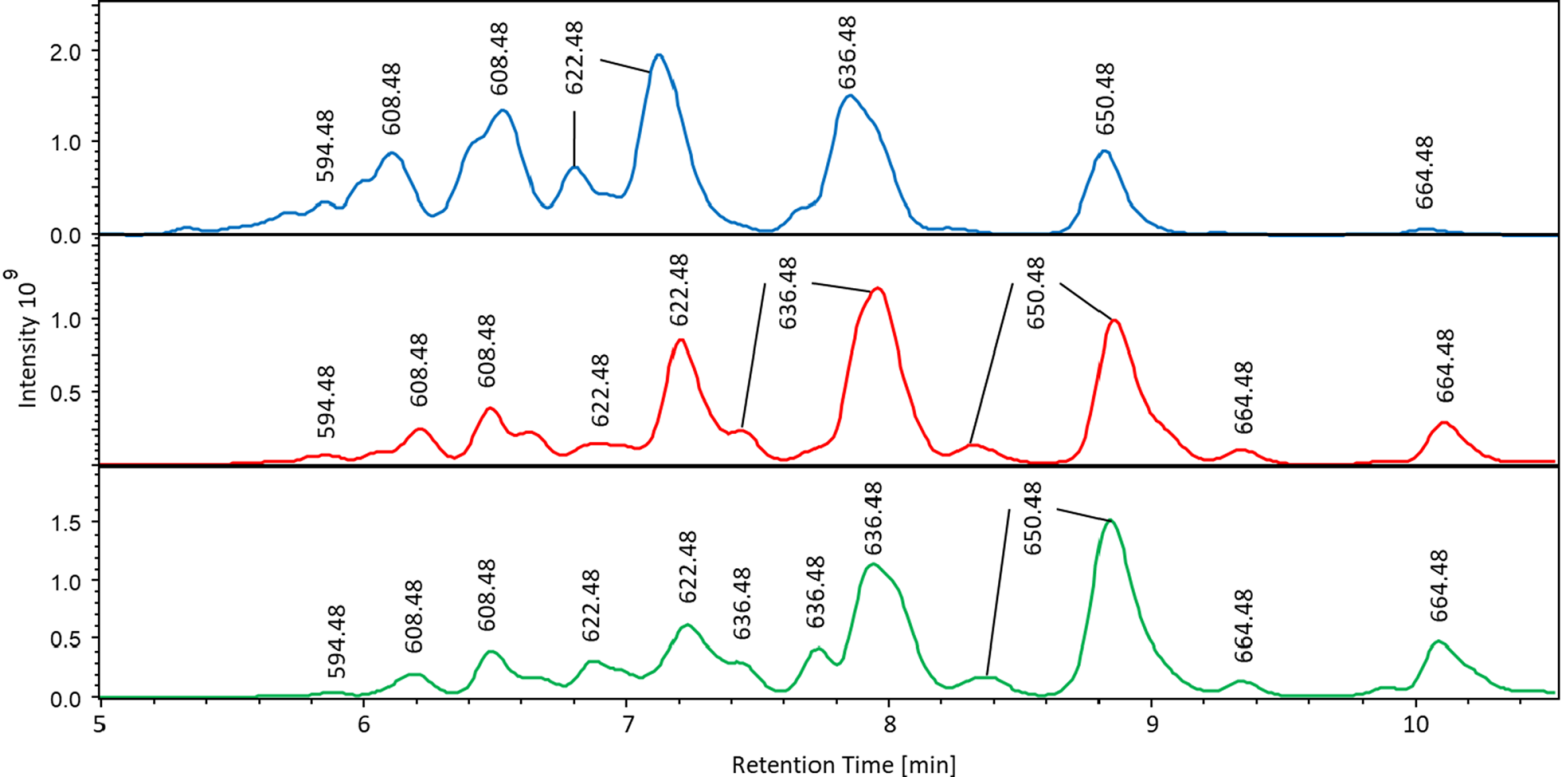
Comparison of the LC–MS chromatograms of *S. albus* J1074 P21pamW containing R2 constructs. (**A**) R2, (**B**) R2-73 and R2-100 cosmids (**C**); Different pamamycins are indicated by the corresponding mass to charge ratio

To avoid biasing which potentially could arise from isolation and retransformation, *E. coli* colonies harboring *pam* BGC library were washed off the plates and directly used in triparental intergeneric conjugation with *E. coli* ET12567 (pUB307) as a helper and *S. albus* J1074 P21pamW as recipient. In total, 106 colonies of transconjugants were selected for further analysis of pamamycins production. These strains were cultivated and pamamycins were extracted and quantified by LC‒MS as described in the materials and methods section. As a control, *S. albus* J1074 P21pamW carrying the parental R2 cosmid was used. Sixty-seven of the 106 selected clones failed to produce any detectable level of pamamycins (Additional file 1: Table S3). In 28 clones the production was restored, however, the yield was significantly lower than that in the control strain. Only 11 strains demonstrated pamamycin production on par or higher than that of *S. albus* J1074 P21pamW with R2 cosmid. Two of these, designated as R2-73 and R2-100, showed significant increase in the accumulation of pamamycins with a molecular weight of 635 Da and higher (Fig. 3).

**Fig. 4:**
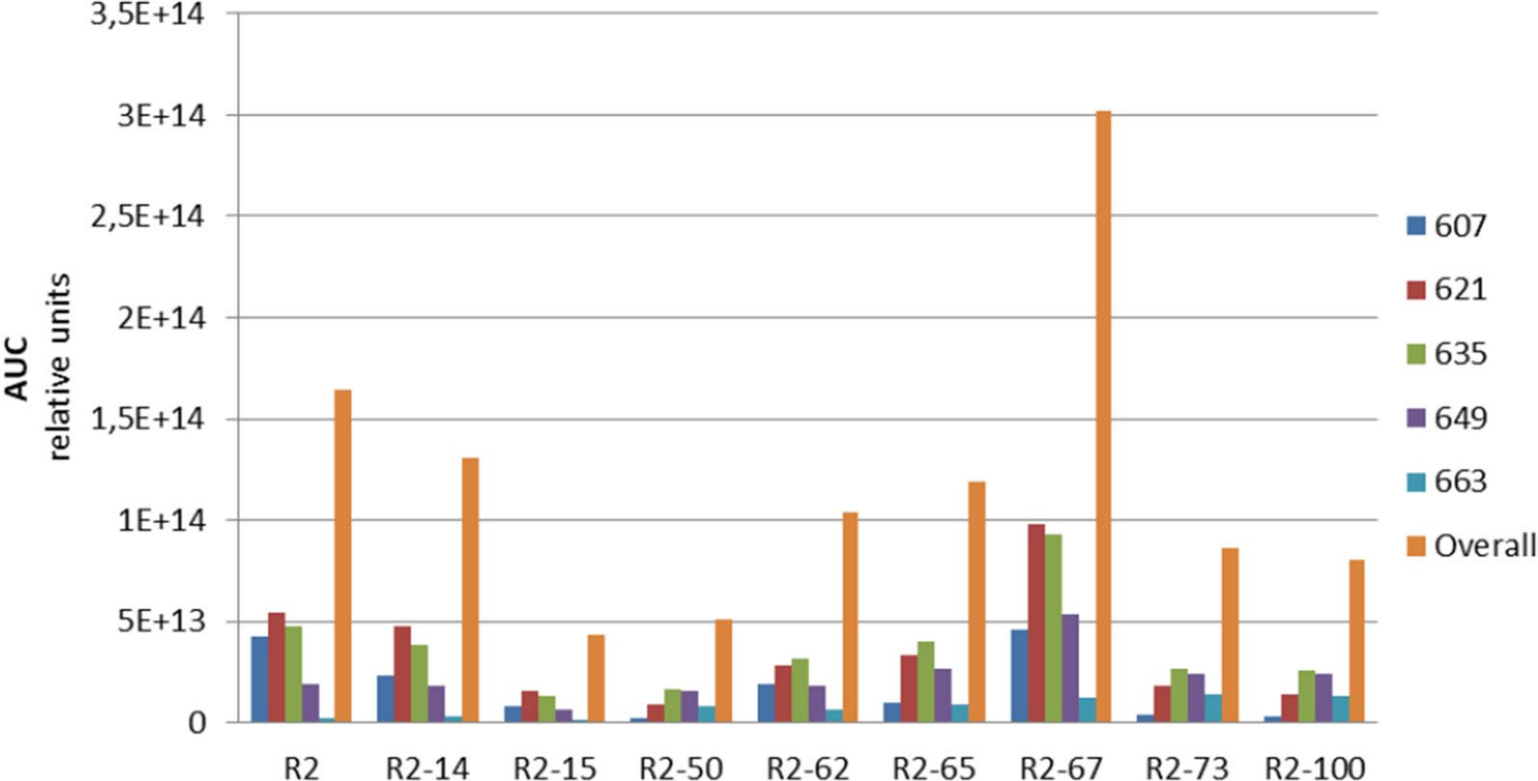
Production of different pamamycin derivatives by *S. albus* J1074 P21pamW strain, carrying engineered R2 cosmids. The pamamycin production was assessed as sum of area under the curve (AUC) of LC–MS peaks with the same m/z value. Pamamycin derivatives are indicated by their molecular weight. Recombinant R2 cosmids are labeled by assigned numbers. Biological triplicates were performed for this experiment (n = 3)

The production of pamamycins 663 by *S. albus* P21pamW R2-73 and R2-100 was 9 and 8 times higher than in the case of the same host carrying the R2 cosmid (Fig. 4). The accumulation of other pamamycins was also affected in the recombinant strains with R2-73 and R2-100 constructs. All compounds with molecular weight of 635 and higher were overproduced at different extent and accumulation of smaller pamamycins was decreased. In fact, the overall pamamycins production was lower in the case of R2-73 and R2-100 when compared to the unmodified R2 cosmid. The highest level of pamamycins production was observed in *S. albus* P21pamW strain with R2-67 cosmid. The overall yield in this strain was almost twofold higher than in the case of the strain with the parental construct. *S. albus* P21pamW R2-67 also demonstrated a shift in the spectra of derivatives with the major increase in accumulation of pamamycins 621 (1.8 times), 635 (1.9 times) and 649 (2.8 times). The pamamycins 663 were also overproduced (fivefold increase) but still did not reach the level of production by the strains with R2-73 and R2-100. Further analysis revealed that another 6 clones had a somewhat shifted spectrum of production of pamamycin derivatives. However, the effect was not as dramatic as in the case of the R2-73 and R2-100 (Fig. 4).

Furthermore, the high-resolution LC‒MS analysis of the extracts of *S. albus* J1074 P21pamW R2-73 strain allowed the detection of pamamycins with MWs of 677 and 691 Da (Additional file 1: Fig. S1), which are absent or produced in miserable amounts by the parental strain. The production level allowed the purification of pamamycins 663 and 677 from the extract of *S. albus* J1074 P21pamW R2-73 in quantities sufficient for structure elucidation.

### Isolation and structure elucidation of new pamamycins

To facilitate the purification of pamamycins 663 and 677 the R2-73 cosmid was transferred into the *S. albus* Del14 strain, which is deficient in intrinsic specialized metabolites production [32]. The use of this strain streamlines the purification of heterologously produced bacterial natural products. The strain *S. albus* Del14 R2-73 was cultivated in 10 L of SGG medium at 29 ℃ for 4 days, and metabolites were extracted as described in the methods section. The crude extract was purified by a two-step chromatography approach. First, the extract was fractionated by size-exclusion chromatography, and then individual pamamycins were purified by reverse-phase chromatography. Three compounds with molecular weight of 635, 663 and 677 Da were purified in quantities sufficient for NMR analysis. Since all pamamycins have the same core structure of the macrodiolide ring and differ mainly by the length and structure of the side chains (Fig. 1), proton NMR was sufficient for analyzing the number of methyl groups and their coupling pattern.

**Fig. 5:**
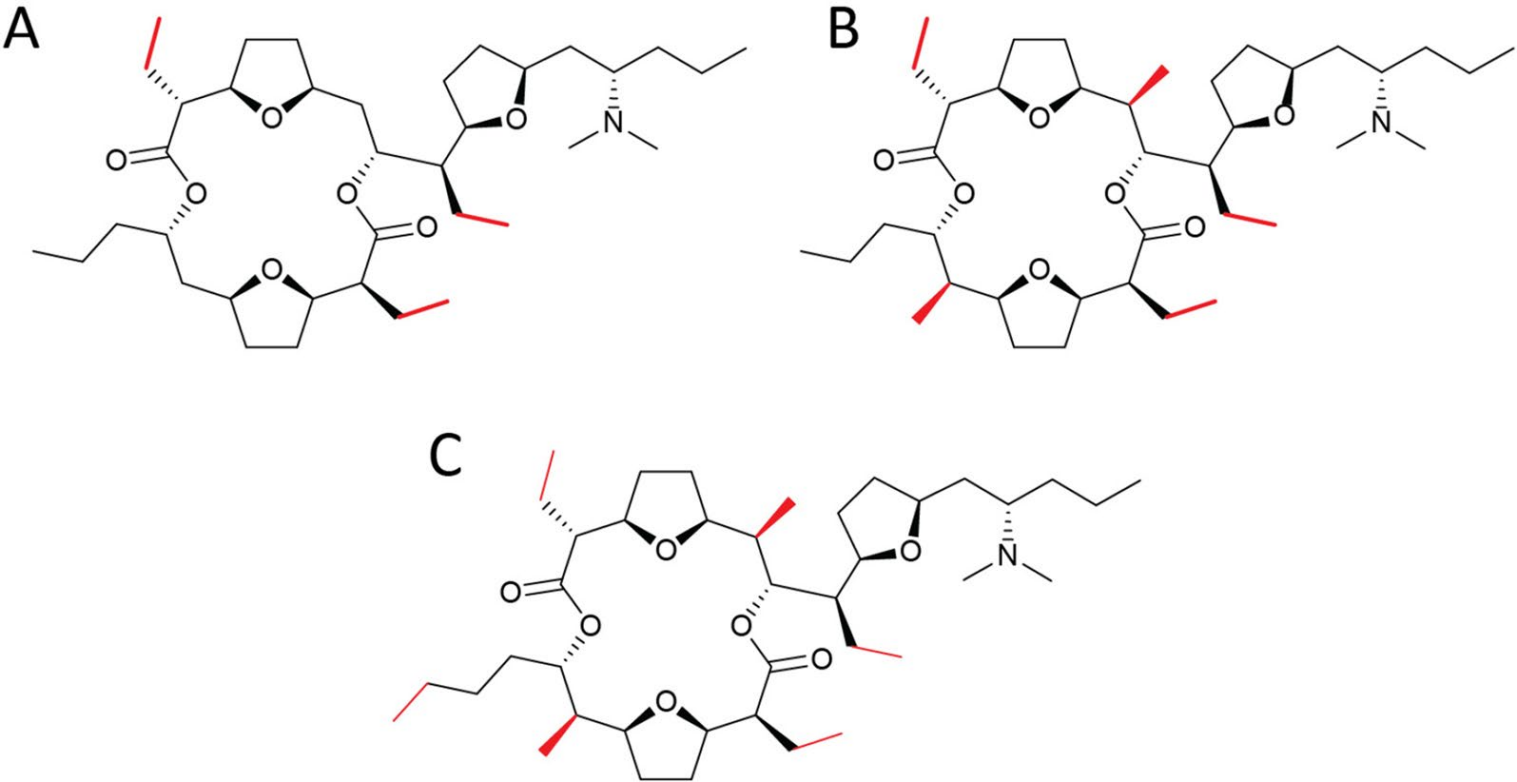
Structures of pamamycin 635G (**A**), pamamycin 663A (**B**) and homopamamycin 677A (**C**). Groups distinguishing these derivatives from previously described are highlighted in red

The core structure of pamamycins and their biosynthesis have been described previously [37]. During biosynthesis, two asymmetric hydroxy acids are produced, which together form the final pamamycin molecule (Fig. 1). Different pamamycins are synthesized by the enzymatic machinery, due to the ability of certain enzymes to accept a broader range of starter and extender units (succinyl-CoA, malonyl-CoA, methylmalonyl-CoA or ethylmalonyl-CoA). The different incorporation pattern might also depend on the availability of the respective units within the cell during different growth phases. Pamamycin 635G, with an exact mass of 635.4761 Da (Fig. 5A), was obtained as a white powder (4.4 mg). It shows only five triplet methyls in addition to the two singlet N-methyl signals in its proton spectrum (Additional file 1: Tables S’1, S’2, Additional file 1: Figs. S’1, S’2). Two of them represent the methyl H-18 and methyl H-11′ chain ends of the two hydroxy acid fragments (Additional file 1: Fig. S’3). This leaves three methyl groups, each of which is presumably attached as part of an ethyl group to the previously known stereogenic centres C-2, C-7, C-9, C-2′ or C-7′ (Additional file 1: Fig. S’5). Since all known pamamycins 635, 635A, 635F [33, 37] and bis-homo-pamamycin-635A [24] bear at least two doublet methyls, pamamycin 635G must be a new pamamycin. 2D NMR measurements support this assumption. According to the analysis of ^1^H-^1^H COSY, HSQCED, HSQC-TOCSY and HMBC (Additional file 1: Fig. S’4–S’7), the three ethyl residues are found at C-2, C-9 and C-2′ (Fig. 5A). Thus, pamamycins 635G is the first known derivative build solely by malonate and ethylmalonate, without involvement of methylmalonate as a precursor.

The NMR data of pamamycin 663A are close to those of pamamycin 635G. However, its MW (exact mass 663.5074 Da) is 28 Da higher, and two additional doublets appear in the proton spectrum, indicating the presence of two methyl groups (Additional file 1: Tables S’3, S’4, Additional file 1: Figs. S’8–S’10). Their positions at C-7 and C-7′ together with the complete assignment can be deduced from the relevant 2D NMR spectra (Additional file 1: Fig. S’11–S’15). No solved structure for a pamamycin with a MW of 663 Da has yet been published. However, since further structural variants are conceivable, we call this compound pamamycin 663A (Fig. 5B). Pamamycin 663A comprises a largest possible derivative with fully saturated side chains’ structure. Further increase of pamamycins in size is possible only by recruitment of larger starting building blocks extending the compound at C-18 and C-12′ positions.

Despite multistep purification, homopamamycin 677A (exact mass 677.5231 Da) still turns out to be a 3 to 1 mixture of two structurally related compounds. Nevertheless, after extensive NMR analysis and comparison of the data obtained with those of pamamycin 635G and pamamycin 663A, a structure can be proposed at least for the main component. The main compound in the mixture has the same number of methyl groups, including the chemical shifts and the coupling patterns, identical to those observed in the case of pamamycin 663A except that chain extension of one of the two hydroxy acid fragments seems to have a place in case of homopamamycin 677A (Additional file 1: Tables S’5, S’6, Additional file 1: Figs. S’16-S’18). A first indication of this position is provided by the ^13^C NMR data of homopamamycin 677A (Additional file 1: Fig. S’19) and the comparison with those of pamamycin 635G (Additional file 1: Fig. S’1). Only the methyl group C-11′ shows a small high-field shift of 0.2 ppm. All other methyl shifts remain almost the same. If to examine the connectivity to its two nearest methylene groups, a significant difference in their ^13^C shifts to the comparative groups at pamamycin 635G could be detected. All this leads to the conclusion that homopamamycin 677A is a homologue of pamamycin 635G, with hydroxy acid small extended by one carbon to C-12′. In analogy to the only chain-extended pamamycin variants known thus far, homopamamycin 621A and bishomopamamycin 635, we named this structure homopamycin 677A. It should be noted that the assignments for homopamamycin 677A were only possible after extensive selective 1D TOCSY measurements (Additional file 1: Fig. S’20–S’30).

### Synthetic promoters delay the pamamycins production onset

**Fig. 6:**
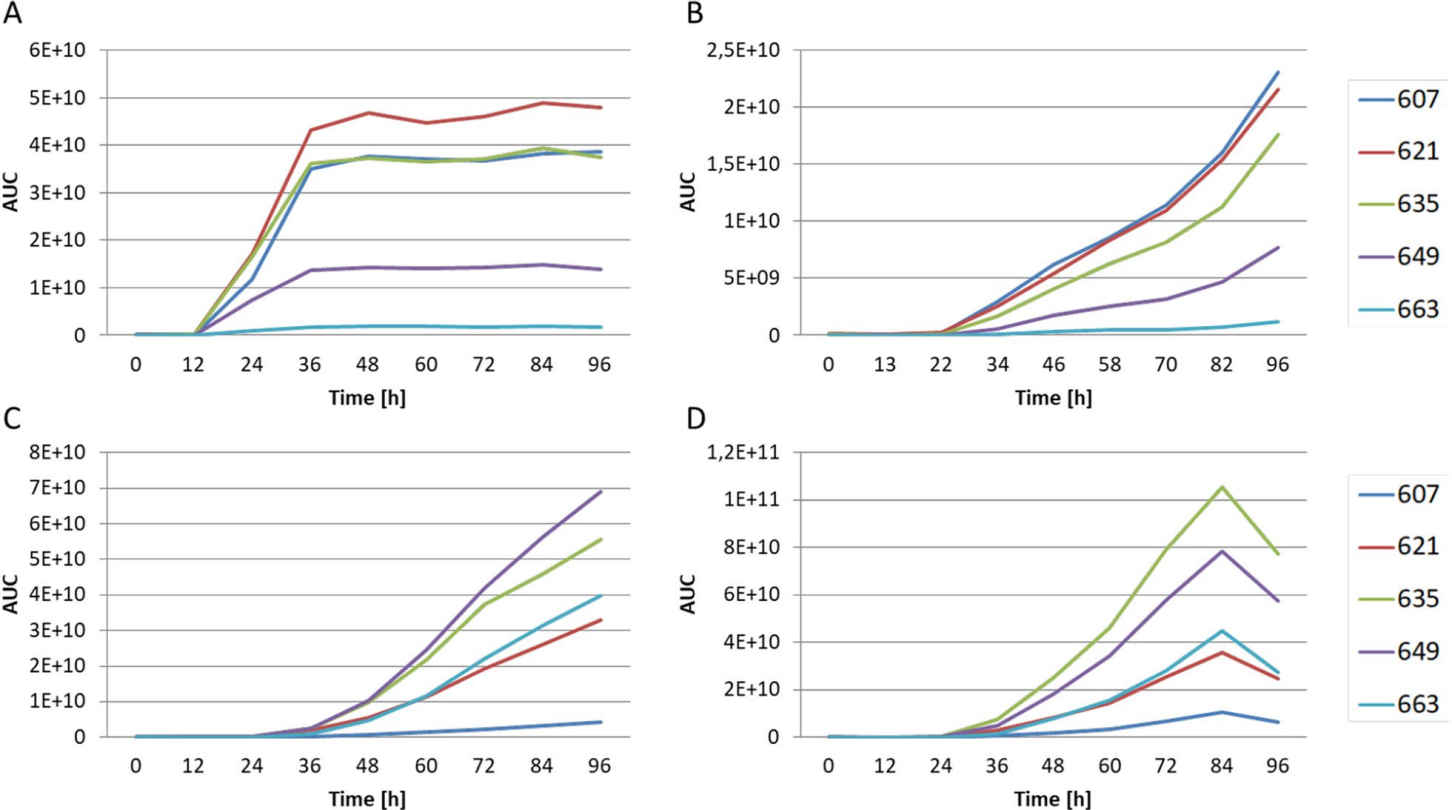
Pamamycin production over time in the different *S. albus* J1074 P21pamW strains. (**A**) R2, (**B**) R2-67, (**C**) R2-73, (**D**) R2-100. Shown is the area under the curve (AUC) for different pamamycins 607–663. Pamamycin derivatives are indicated by their molecular weight. Biological triplicates were performed for this experiment (n = 3)

The timing of bacterial natural products production is often an important factor influencing the yield. The onset of specialized metabolism is tightly controlled by physiological and other factors, and the majority of BGCs contain one or more regulatory genes, some of which act as activators of the transcription of structural genes [46]. Transcriptional regulation of the *pam* BGC is governed by the PamR1 transcriptional activator [37]. The placement of *pam* biosynthetic genes under PamR1 independent promoters might influence the time of onset of pamamycins production which could be at least one of the reasons for the observed shift in the derivatives spectra. To investigate this, an analysis of pamamycin production at different time points was performed. The *S. albus* J1074 P21pamW strains carrying parental R2 cosmid and engineered R2-67, R2-73 and R2-100 were studied. Strain with R2-67 was chosen as such with the highest level of overall production but with the derivatives profile close to the parental R2 construct. The strains were cultivated over a period of 4 days in SGG medium at 29 ℃. The samples were harvested every 12 h and pamamycins production was monitored by LC‒MS. *S. albus* J1074 P21pamW R2 carrying native *pam* BGC starts to produce pamamycins at 12 h of growth and rapidly reaches the maximum of production at 36–48 h (Fig. 6A). In all three strains carrying the modified gene clusters, the onset of pamamycins production was strongly delayed. The compounds could be detected in the extracts of the corresponding strains only after 24 h of growth (Fig. 6). Furthermore, pamamycins production in case of R2-67 and R2-73 carrying strains did not reach a plateau even after 96 h of cultivation. *S. albus* J1074 P21pamW R2-100 demonstrated the decrease in the pamamycin concentrations in culture medium after 84 h. At the same time all strains carrying engineered cluster accumulate more biomass when compared with the R2 harboring strain even at the late stages of growth when the autolysis occurs (Additional file 1: Fig. S2).

The obtained data demonstrates that the artificial promoters driving the transcription of key biosynthetic operons in *pam* BGC are turned on later than the corresponding native promoters. Therefore, the delay in the transcription of the *pam* gene cluster allows the recombinant strains to accumulate biomass before the onset of pamamycins production, diminishing their severe toxic effect. However, this delay in production could not explain the changes in the metabolic profiles of *S. albus* J1074 P21pamW with R2-73 and R2-100, since the strain containing the R2-67 cosmid with likewise synthetic promoters shows only a slight overproduction of heavier pamamycins and an overall production profile more similar to that of *S. albus* J1074 P21pamW R2.

### Analysis of transcription profiles of recombinant pam BGCs

The promoters of the *pamA* and *pamF* genes in the recombinant constructs R2-67, R2-73 and R2-100 were amplified from the genomic DNA of corresponding *S. albus* strains and sequenced. The comparison of these promoters with the *ermE*p1, which served as a template, did not show any clear dependencies or trends in the sequence of 5′ untranslated regions (Additional file 1: Fig. S3, Additional file 1: Table S4). However, the spacer between − 10 and − 35 regions of the *pamF* promoter in the R2-67 construct has 37% GC content. At the same time, the spacer in the *pamF* promoter of R2-73 and R2-100 has a GC content of 58 and 53%, respectively. In the case of *pamA* promoters, the GC content of the spacer region is 53%, 42% and 58% in R2-67, R2-73 and R2-100, respectively. The same region of the parental *ermE*p1 promoter has 63% GC pairs. No other peculiarities could be deduced from the sequence analysis.

**Fig. 7:**
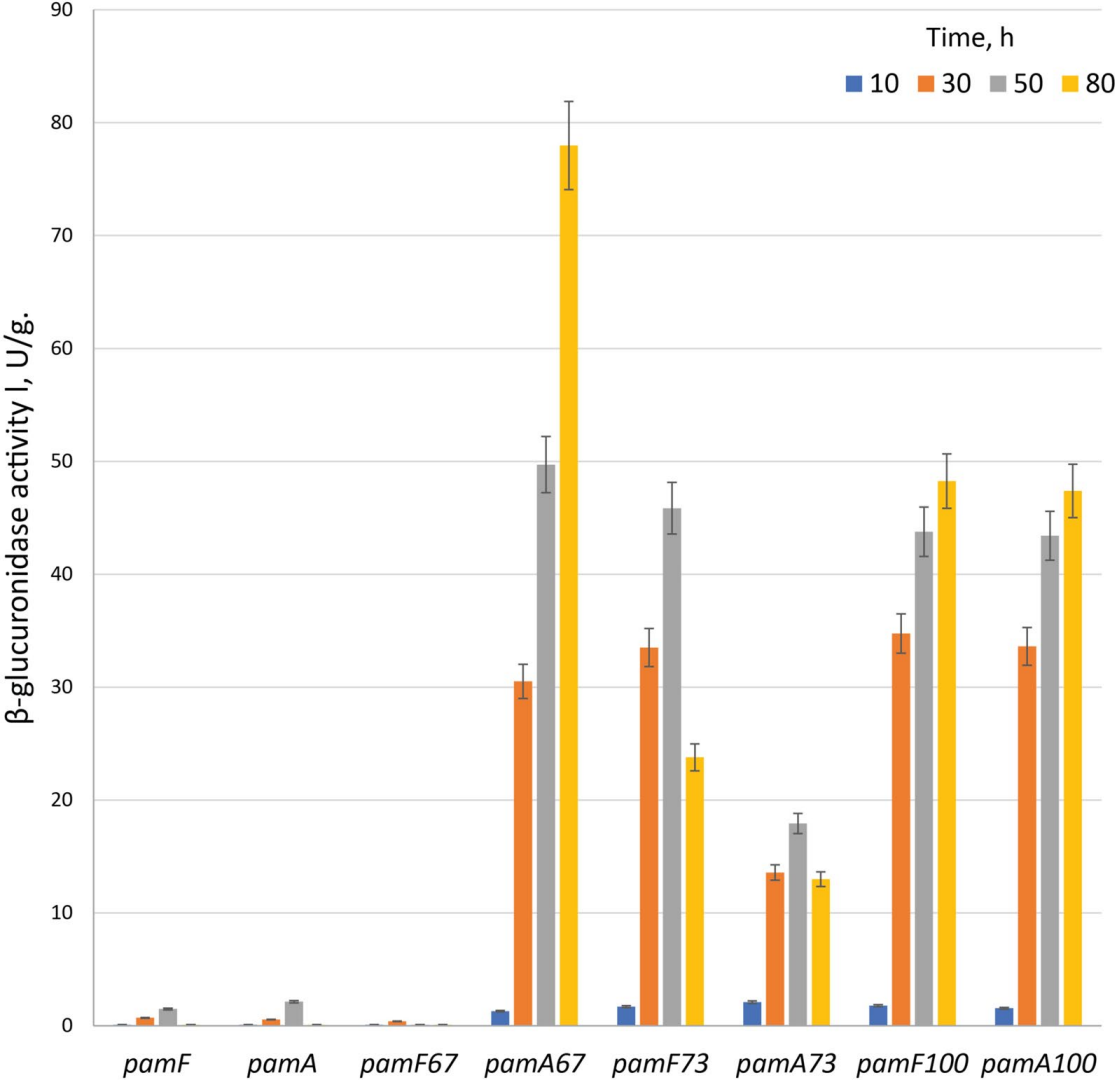
β-glucuronidase activity in cell lysates of *S. albus* J1074 containing *uidA* gene under control of different promoters. The native and synthetic *pamA* and *pamF* genes promoters from the R2, R2-67, R2-73 and R2-100 cosmids were used. The strains were grown for 10, 30, 50 and 80 h in SGG medium. *S. albus* J1074 pGUS was used as the blank sample. The activity was normalized to dry biomass weight. Biological triplicates were performed for this experiment (n = 3)

Due to the differences in the promoter sequences of both *pamA* and *pamF* genes in recombinant R2 cosmids, we decided to quantitatively assess their activity by fusing to the *uidA* reporter gene [45]. The promoters were incorporated into primers that were used to amplify a Hyg^R^ cassette and cloned into the pGUS reporter vector to create transcriptional fusion with the promoterless *uidA* gene. Native *pamA* and *pamF* promoters were used as control. The resulting plasmids were transferred into *S. albus* J1074 and tested for β-glucuronidase activity at different time points (10, 30, 50 and 80 h) during growth in SGG medium (Fig. 7). At 10 h of growth very low or no activity was detected in all tested strains. This coincides with the observed onset of pamamycins production which occurs at 12 h of cultivation or later. This is still surprising, since the *ermE*p1 promoter and thus its derivatives were expected to be constitutive. The native *pamA* and *pamF* promoters’ activity was increased at 30 h time point and reached the maximum at 50 h of cultivation; after that a significant drop of activity was observed. Similar, synthetic promoter’s activity was not observed at 10 h of growth and raise dramatically at 30 h of cultivation.

In the case of both *pamA* and *pamF* promoters obtained from R2-73 and R2-100 cosmids, the activity continues to grow over time reaching the maximum at 50 h with insignificant drop at 80 h in the case of and R2-73. However, the synthetic promoters surpassed the activity of native ones at all tested time points. The difference in activity between native and R2-73 *pamA* promoters reached eightfold at 50 h of cultivation, when the same promoter from the R2-100 construct was 20 times more active. Similarly, *pamF* promoters from R2-73 and R2-100 at 50 h’ time point were 30 and 29 times more active than the native. Different situation was observed in the case of the R2-67 construct. The promoter of *pamA* gene was significantly stronger than the native. Furthermore, this promoter outperformed all other tested reaching 78 U/mg of β-glucuronidase activity at 80 h of culture growth. However, the activity of *pamF* promoter from R2-67 was on par with the native. It was significantly lower and reached the maximum at 30 h of growth. At 50 h’ time point the β-glucuronidase activity driven by R2-67 *pamF* promoter was at the lower limits of detection (Fig. 7).

**Fig. 8:**
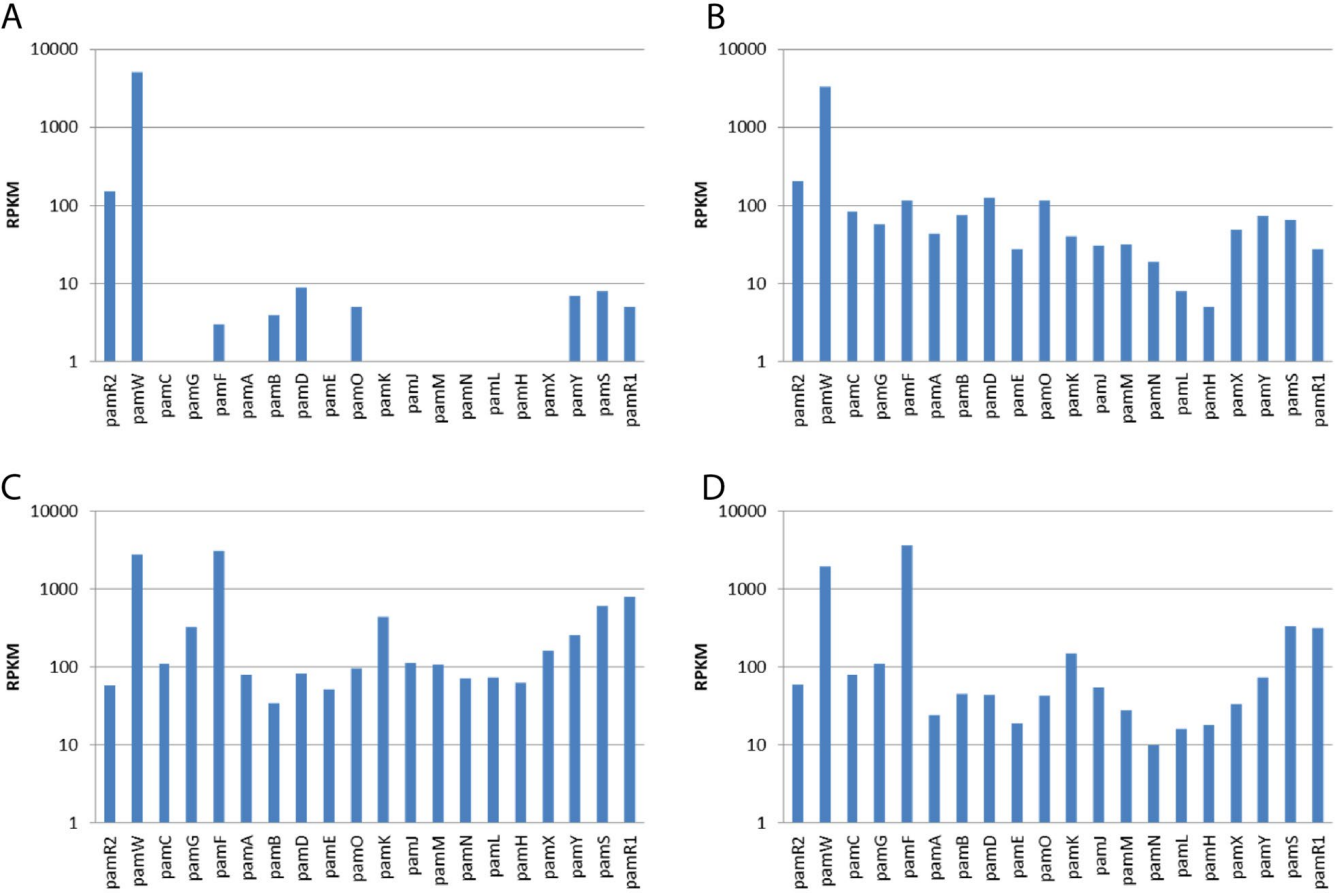
Transcription profile of the pamamycin gene cluster variants determined with RNA-seq approach. (**A**) *S. albus* J1074 P21pamW R2, (**B**) *S. albus* J1074 P21pamW R2-67, (**C**) *S. albus* J1074 P21pamW R2-73, (**D**) *S. albus* J1074 P21pamW R2-100. Biological triplicates were performed for this experiment (n = 3)

The promoter fusion allows detecting the activity of the studied promoter; however, it did not disclose the full transcriptional properties of the studied cluster. The transcriptional profiling of *S. albus* P21pamW R2, R2-67, R2-73 and R2-100 using the RNA-seq approach was performed at 50 h or cultivation in production media (Fig. 8). The obtained sequences were mapped to the genome of *S. albus* with the inserted *pam* BGC in loci that correspond to the *attB* site of the phiC31 actinophage. The transcription profile of the native R2 cosmid confirmed the previous observation regarding the activity of native *pamA* and *pamF* gene promoters: almost all structural genes are transcribed at very low level, barely reaching 10 rpkm. However, this level of transcription still seems to be sufficient for the production of pamamycins. The only genes that demonstrated significant activity were *pamR2* and *pamW*. These two genes have similar high transcription rates in all studied strains. In the case of *S. albus* R2-67, a transcriptional profile similar to that of the R2-carrying strain was observed, with only difference that the overall level of transcription was 10-folds higher and more consistent in the case of the engineered construct.

In the case of *S. albus* P21pamW R2-73 and R2-100, the transcriptional activity of the entire cluster was also higher than that of the R2-carrying strain (Fig. 8). However, in contrast to the R2-67, the level of transcription of the *pamF-pamC* operon was dramatically increased, reaching the same or an even higher than transcription of the *pamW* gene, which is one of the most active genes in the entire genome of *S. albus* (data not shown). Moreover, transcription of the *pamA-pamS* operon was increased but not as strongly as that of the *pamF-pamC* operon. The differences in expression of *pamF-pamC* operon in R2-73, R2-100 and R2-67 constructs makes us to think that the overexpression of this operon, encoding enzymes required for hydroxy acid large biosynthesis, could be the reason for the observed changes in the profile of accumulated pamamycins.

### Pamamycin production depends on balanced expression of both the pamA and pamF operons

**Fig. 9:**
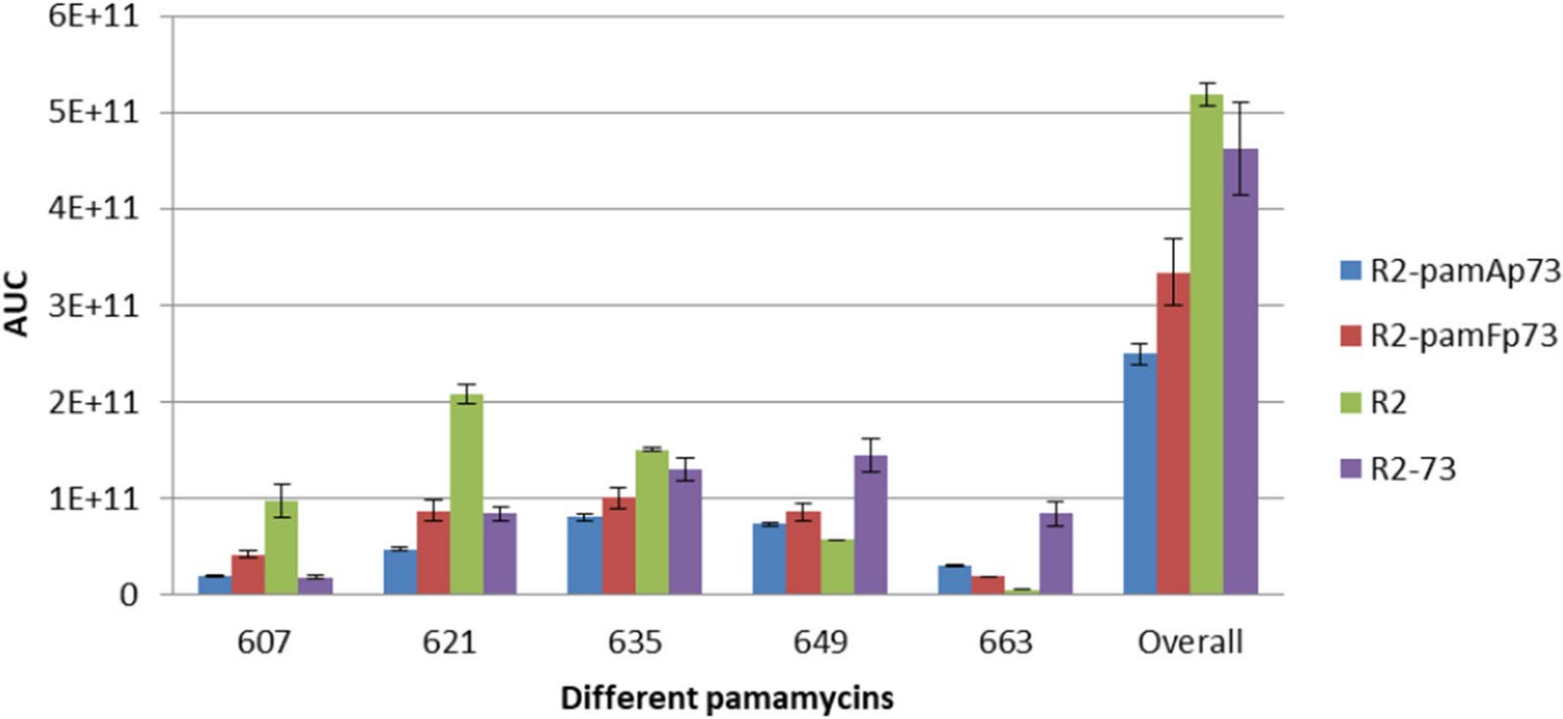
Pamamycin production by *S. albus* P21pamW J1074 containing different variants of the R2 cosmid. The synthetic promoters driving the expression of either *pamA-pamS* operons (R2-pamAp73) or *pamF-pamC* (R2-pamFp73) and both operons (R2-73), were transferred into the R2 cosmid. The native construct R2 was used as control. Biological triplicates were performed for this experiment (n = 3)

The reporter fusion and transcriptomics data demonstrated that the transcriptional control of the *pam* BGC is affected in the case of the engineered constructs. These changes lead to a disbalance in the production of biosynthetic enzymes, which could be the reason for the observed changes in the profile of accumulated pamamycins. To investigate the contribution of overexpression of either *pamA-pamS* or *pamF-pamC* operons to the observed phenotype, two *pam* BGCs were constructed by replacing the promoter of either *pamA* or *pamF* with the respective promoters from R2-73 while preserving the native sequence of opposite one. In this way cosmids R2-pamAp73 (with the synthetic promoter from R2-73 driving the transcription of the *pamA-pamS* operon) and R2-pamFp73 (with the synthetic promoter from R2-73 driving the transcription of the *pamF-pamC* operon) were generated. These constructs were introduced into *S. albus* J1074 P21pamW, and the production of pamamycins was analyzed by LC‒MS. In the case of R2-pamAp73 and R2-pamFp73 carrying strains, a slight increase in the accumulation of high-molecular-weight pamamycins was observed; however, it was not as significant as in the case of recombinant BGC R2-73 with replacement of both promoters (Fig. 9).

Furthermore, the overall production of pamamycins was lower in the case of the R2-pamAp73 and R2-pamFp73 constructs when compared with R2 and R2-73. Therefore, the observed phenotype in the case of strains carrying the R2-73 and R2-100 cosmids is most likely caused by a synergetic effect of the synthetic promoters. The expression of both operons needs to be increased to different extents to reach high production, as well as to cause a shift towards high molecular weight pamamycins.

### Biological activity of high molecular weight pamamycins

Pamamycins are promising antibacterial metabolites. However, there is a limited understanding of structure–activity relationships within this family of natural products. In a course of compound purification pure samples of pamamycin 607, 649A, and 663A were obtained and tested in a number of activity assays. The antibiotic activity was evaluated against Gram-positive and Gram-negative bacteria. The cytotoxicity test was performed on KB-3.1 (cervix carcinoma) and HepG2 (hepatocellular carcinoma) cell lines. The general toxicity of compounds was assessed on zebrafish, and the herbicidal activity was tested on *Agrostis stolonifera*. All tested compounds demonstrated weak or no activity against Gram-negative test cultures with MIC values ranging from 32 µM and higher (Additional file 1: Table S5). In the case of Gram-positive bacteria, all three compounds were found to be highly potent with smaller pamamycins being less active than the large one. Pamamycin 607 demonstrated MIC ranging from 8 to 16 µM depending on the test culture used when pamamycin 663A has much better activity with MIC values of 1 to 8 µM. The latter derivative has an MIC of 1 µM against both *Staphylococcus aureus* laboratory strain Newman and MRSA strain N315, as well as against *Streptococcus pneumoniae.* All three tested pamamycins were able to prevent the growth of *Mycobacterium smegmatis* at concentrations 8–16 µM*.* At the same time, they also showed high cytotoxicity on carcinoma (KB-3.1) and hepatocyte (HepG2) cancer cell lines (Additional file 1: Tables S6). In these experiments a similar to antibacterial activity trends were observed: the high-molecular-weight compounds were more active. The IC50 of pamamycin 663A on the HepG2 cell line was extraordinarily low reaching 2 nM which is 310 times lower than IC50 of pamamycin 607. The doxorubicin, which was used as a control, demonstrated IC50 of 0.29 nM. All tested pamamycins were toxic against zebrafish at different stages of development and demonstrated only some minor activity in the herbicidal test against *Agrostis stolonifera* (Additional file 1: Tables S7, S8).

## Discussion

The progress in genome sequencing made clear that the specialized metabolism potential of *Actinobacteria* has not yet been exploited in full [3, 5, 8, 38]. Therefore, the cryptic gene clusters are potential targets for new natural drug lead discovery. The development of methods and approaches leading to the activation of cryptic gene clusters is a key priority in the synthetic biology of Actinobacteria [35]. Here, we demonstrated that decoupling of the transcription of pamamycin biosynthetic genes from the cellular regulatory network as well as changing the natural balance of gene expression could be used as a tool for modulating the production of specialized metabolites. Such decoupling could be achieved by rational engineering of promoters of corresponding biosynthetic genes and operons. However, the danger of such an approach lies in the imbalance of expression which could lead to production of shunt products but not the target compound as in the case of landomycin gene cluster [28]. On the other hand, the precise tuning of expression of biosynthetic genes and operons could be achieved by an approach based on randomization of corresponding promoters coupled with the intensive screening. This rather laborious way still results in a target compound overproduction as it was in the case of engineered bottromycin gene cluster [19]. The latter approach could be successfully applied to diverse high value natural products which are typically produced at very low quantities. In this work we have demonstrated the efficiency of the latter approach on pamamycins, an unusual family of polyketide antibiotics.

Pamamycins are produced in a mixture of derivatives ranging from 579 to 705 Da. The native cluster when expressed in heterologous host results in primary accumulation of smaller derivatives with molecular weights of 579, 593, 607, 621 and 635 Da, whereas pamamycins 649 and 663 are barely detectible, preventing their isolation and characterization (Figs. 1 and 3). By introducing synthetic derivatives of the *ermEp1* promoter, the transcription of two main operons of the pamamycin BGC was rebalanced, which led to a shift in a spectrum of accumulated derivatives (Fig. 3). The high molecular weight pamamycins production was significantly increased, which enabled purification and structural characterization of new derivatives with molecular weight 663 and 677 Da. These two compounds named as pamamycin 663A and homopamycin 677A are the largest pamamycins with known structure to the date. Together with the pamamycin 635G they expand this family of natural products with new derivatives, bringing deeper understanding of the flexibility of corresponding biosynthetic enzymes in their selection of starter and extender units. Furthermore, also discovered in a course of this project pamamycin 623G represent the derivatives with the largest possible side chain substituents at positions C-2 and C-2′. At the same time, when pamamycin 663A and homopamycin 677A have fully saturated side chains and thus represent the largest possible structure of pamamycins’ macrodiolide ring. The further increase in size of pamamycin 663A is possible solely by implementing larger starter units by the corresponding enzymatic assembly like. In fact, homopamycin 677A could be seen as an outcome of such starter unit selection: in the case of hydroxy acid small instead of usual propionate the butyrate is used. This compound is the first reported pamamycin with the extension of hydroxy acid small, since all other derivatives with the other than propionate starter had longer hydroxy acid large (homopamamycin 621A and bishomopamamycin 635A) [23].

The observed shift in the production of pamamycin derivatives is obviously caused by the changes in the efficiency of expression of the two engineered operons. These operons are coding for key biosynthetic enzymes involved in the assembly of hydroxy acid small (*pamA-pamS*) and its extension to hydroxy acid large (*pamF-pamC*) [37]. The lack of *pamC* function was described to cause the accumulation of small molecular weight derivatives clearly pointing on its importance in assembly of large pamamycin derivatives. The transcriptional studies of engineered *pam* BGCs revealed that besides the increase in overall level of transcription the *pamF-pamC* operon was significantly overexpressed when compared to other *pam* genes. Such increase in transcription of *pamF-pamC* operon was observed in the case of R2-73 and R2-100 constructs, but not in R2-67, which has a similar to native profile of produced pamamycins. Overexpression of ACP (acyl carrier protein) is known to increase the production of other actinobacterial polyketide natural products, for instance tetracenomycin and its derivatives [7]. In the case of pamamycins, it can be proposed that ACP is mediating the preferential recruitment of ethylmalonyl-CoA into the assembly line, somehow influencing the specificity of biosynthetic enzymes.

At the same time, the balance in expression of *pamF-pamC* operon and other biosynthetic genes seems to be important for proper functioning of pamamycins assembly line. The *pam* BGC, in which the promoter of *pamF* was replaced with the strong synthetic promoter from R2-73 construct, demonstrated no changes in the spectra of derivatives. Furthermore, the overall pamamycins production was decreased. This observation reflects the complexity of transcriptional organization of biosynthetic gene clusters. In case of *pam* BGC the expression of two operons *pamF-pamC* and *pamA-pamS* have to be well balanced. Even more, the entire BGC expression has to be properly orchestrated in order to achieve the optimal production of target metabolite. The imbalance in transcription of biosynthetic genes seems to affect the assembly line resulting in sub-par efficiency of biosynthesis.

On the other hand, the replacement of native promoters with the engineered ones led to the delay in onset of pamamycins production. It is not clear if such delay is caused by the properties of *ermE* core promoter sequences. In fact, ermE*p was reported to be constitutively expressed in *Saccharopolyspora erythraea* and is equally active at 24, 48 and 72 h of growth in *S. albus J1074* [26]. The changes in the randomized spacer regions of the engineered promoters seem to affect the timing of their onset. However, the influence of other factors, such as availability of specific sigma factor RNA polymerase, could not be excluded. Regardless of the actual reasons for observed production delay, it could be a useful feature of the producing strain construction. In many biotechnological applications the two-steps process is beneficial for the final product yield, especially when it comes to the toxic compounds. At the first phase the culture is actively growing and after a certain biomass is reached it switches to the production phase. The observed delay in onset of pamamycins production could be another factor which allowed the production of high molecular weight derivatives. The activity tests clearly demonstrated that larger pamamycins are more toxic against bacterial cultures as well as other tested organisms. This could explain the fact that the *S. albus* strains with recombinant cosmids with much higher level of expression of biosynthetic genes still have not outreach the overall production level of pamamycins observed in the case of strain carrying native R2 cosmid. The shift in accumulation toward larger and thus more toxic derivatives could limit the yield. In fact, strain with R2-67 construct, which has similar spectra of derivatives as the native R2, demonstrated twice higher production level then the latter. Additionally, all recombinant strains with the engineered BGC have higher biomass accumulation when compared to *S. albus* J1074 with native *pam* gene cluster. Finally, the delay of the production onset could also contribute to the observed changes in the spectra of produced pamamycins. It was noted that the intracellular concentration of ethylmalonyl-CoA is increasing over time in case of *S. albus* carrying R2 cosmid [12]. Thus, the late expression of biosynthetic genes might coincide with the ethylmalonyl-CoA precursor accumulation leading to production of saturated side chain pamamycins like 635G, 663A and 677A.

The increase in production of heavy pamamycins enabled their isolation and characterization. Pamamycin 663A was found to have strong cytotoxicity being 310 times more active against HepG2 cancer cell line than pamamycin 607. This compound also showed eightfold higher activity against Gram-positive bacteria (Additional file 1: Table S5, S6, S7 and S8). On the other hand, all tested pamamycins were found to be highly toxic on zebrafish tests making them barely suitable for further development. However, the mode of action of this group of natural products still needs to be elucidated in details as well as structure–activity relationship. To date, the structure of 18 pamamycin derivatives have been described, but taking into account the chemical core of these compounds and proposed biosynthesis, at least 288 variants are possible [37]. The understanding of structure–activity dependencies could change the situation with the general cytotoxicity and promote the synthetic or bioengineering studies of this group of compounds.

Summarizing the above data, the proposed transcriptional refactoring approach could be applied to modulate the transcription of specialized metabolites biosynthetic genes with preserving the balance in their expression, thus keeping unchanged the ratio of corresponding enzymes in the assembly line [19]. It could be successfully used to boost the production level of desired compound or awaken the cryptic pathway. It is quite possible that imbalance of expression caused by introduction of artificial promoters into the silent BGC could be one of the reasons for unsuccessful attempt of their activation. However, the slight changes in transcription balance which seems to be tolerated by the assembly line could have a positive effect, as observed in the case of pamamycin BGC, resulting in production of new derivatives which could not be obtained in other ways.

## Conclusion

Transcriptional refactoring of biosynthetic gene clusters may lead to the discovery and sustainable supply of the corresponding natural products, opening the way for their pharmaceutical development. Using this approach, we have discovered and isolated three new pamamycins to conduct initial activity studies. Some of these new derivatives possess much stronger anticancer activity compared to the previously described pamamycin molecules. Therefore, pamamycins could be interesting targets for further studies to unveil their mode of action and potential development. Furthermore, this approach can be extended to other biosynthetic gene clusters, potentially resulting in a significant yield of previously neglected compounds that could be developed for pharmaceutical purposes.

## Materials and methods

### Bacterial strains and culture conditions

The bacterial strains used in this study are listed in Additional file 1: Table S1. *E. coli* strains were grown in liquid lysogeny broth (LB) or solid LB containing 2% (w/v) agar at 37 ℃. Targeted homologous recombination (Red/ET) was performed with the use of the *E. coli* GB05-redCC strain (Additional file 1: Table S1) [11]. *Streptomyces* strains for sporulation were grown on mannitol soy agar for 5–7 days at 29 ℃ [18, 22]). For metabolite production, *Streptomyces* precultures were cultivated in 20 mL of tryptic soy broth (TSB, Sigma‒Aldrich, USA) in a 100 mL flask (4 baffles) with 4 mm diameter glass beads (Carl Roth; Germany) for 1 day at 29 ℃ and agitated at 180 rpm with the respective antibiotics if necessary. The main culture was cultivated in 50 mL of optimized starch glucose glycerol medium (SGG) in a 300 mL flask with one baffle, and glass beads were inoculated with 1 mL of preculture and incubated under the same conditions for 90 h, if not stated otherwise. Optimized SGG medium: 3 *g* of calcium carbonate, 2.5 *g* of corn steep powder, 10 *g* of glycerol, 5 *g* of peptone, 1 *g* of sodium chloride, 10 *g* of starch and 2 *g* of yeast extract were dissolved in 1 L of demineralized water, and the pH was adjusted to 7.2 prior to autoclaving. All chemicals used in this work are supplied by Carl Roth, Germany and Sigma-Aldrich, USA, if another is not stated. Antibiotics were added to the culture at the following concentrations: 50 µg/mL apramycin, 50 µg/mL kanamycin, 120 µg/mL hygromycin, and 50 µg/mL nalidixic acid (Carl Roth, Germany; Sigma-Aldrich, USA).

### DNA manipulations

Chromosomal DNA from *Streptomyces* strains and plasmid DNA from *E. coli* were isolated using standard protocols [22, 43]. Restriction enzymes and molecular biology reagents were used according to the manufacturer’s protocol (NEB, USA; Thermo Fisher Scientific, USA). *E. coli* transformation and intergeneric conjugation were performed as described [10].

### Construction of the *S. albus* J1074 strains

Genes *pamW* and *pamS* were amplified using primer pairs 1 and 2 (Additional file 1: Table S2) and cloned into plasmid pUWLhyg under the control of ermE*p promoter by *EcoR*V-*Pst*I. The constructed plasmids were transferred into *S. albus* J1074 by intergeneric conjugation [10]. Recombinant strains were tested for papamycin 607 resistance with the MIC protocol as described below. The *pamW* gene was amplified using Q5 DNA polymerase (NEB, USA) and primer pair 3 (PamWIntro-F/PamWIntro-R) (Additional file 1: Table S2). The obtained DNA fragment was directly cloned into the *Sna*BI-linearized pTOS vector by blunt-end ligation. As a result, the pTOS-P21pamW plasmid was obtained (Additional file 1: Table S1). The plasmid was verified by sequencing using primers pTOS-F and pTOS-R (Additional file 1: Table S2). The constructed plasmids were transferred into *S. albus* J1074 by intergeneric conjugation [10]. Exconjugants were used as hosts for the screening of the recombinant *pam* BGC library.

### Construction and screening of the recombinant pamamycin BGC library

The *S. alboniger* genomic library cosmid clone R2, carrying the entire pamamycin BGC, was used to construct the randomized promoter library. For this purpose, a hygromycin resistance cassette (Hyg^R^) from the IMES system [31] was amplified using primer pair 5 (PamPrandomHyg^R^-F/PamPrandomHyg^R^-R) (Additional file 1: Table S2). These primers contain a 20 bp region that anneals to the Hyg^R^ cassette, and 50 bp sequences which correspond to the 5′ ends of the *pamA* and *pamF* genes, facilitating recombination, and onward-oriented 40 bp randomized sequences of the *ermEp1* promoter with preserved conserved − 10 and − 35 regions [19]. The obtained PCR fragment was used for Red/ET recombination and facilitated a replacement of the native *pamA* and *pamF* promoters within the R2 cosmid (Fig. 2). Colonies were selected on LB agar plates with 120 μg/mL hygromycin. As a result, a library of the R2 cosmid with random promoters upstream of the *pamA* and *pamF* genes was obtained. Ten independent clones were verified by enzymatic digestion and sequencing using primers Hyg^R^-Seq-F and Hyg^R^-Seq-R (Additional file 1: Table S2). The colonies were washed off the plates after transformation and used in triparental conjugation to transfer the recombinant R2 cosmids into the *S. albus* host strains [10]. A total of 106 colonies of obtained transconjugants were tested for pamamycins production. The randomized promoters from selected clones were amplified and sequenced using primers Hyg^R^-Seq-F and Hyg^R^-Seq-R (Additional file 1: Table S2).

### Construction of R2-pamAp73 and R2-pamFp73 cosmids

Recombinant cosmid clones R2-pamAp73 and R2-pamFp73 were constructed as follows. The hygromycin resistance cassette was amplified with primer pairs 7 and 8 (Additional file 1: Table S2). Primer R2-P73-Down-R (set 7) contains outwards-oriented synthetic promoter of *pamA* operon from R73 recombinant cosmid (pamAp73). Primer R2-P73Up-F contains outwards-oriented synthetic promoter of *pamF* operon from R73 recombinant cosmid (pamFp73). The second primer in each pair contains a region of homology to the pamamycin cluster maintaining the native promoter of opposite operon. The obtained fragments were incorporated into the R2 cosmid via Red/ET recombination to generate the cosmids R2-pamAp73 with synthetic p73 promoter in front of *pamA* operon and native promoter of *pamF* operon and R2-pamFp73 with synthetic p73 promoter in front of *pamF* operon and native promoter of *pamA* operon (Additional file 1: Table S1). The constructs were verified by sequencing. The obtained cosmids were transferred into *S. albus* via conjugation, and pamamycin production was analyzed.

### Determination of relative promoter strength

The native promoters of *pamA* and *pamF* as well as synthetic promoters of these genes from the R2-67, R2-73 and R2-100 clones were cloned by PCR using primer pairs 9–18 (Additional file 1: Table S2). The obtained PCR products, which include the hygromycin resistance cassette, were digested with *Spe*I and *Xba*I and cloned into the *Spe*I/*Xba*I-linearised pGUS vector resulting in transcriptional fusion with the promoterless *uidA* reporter gene (Additional file 1: Table S1). Promoter fusion constructs were selected by resistance to hygromycin. The obtained constructs were verified by sequencing using primers Hyg^R^-Seq-F and Hyg^R^-Seq-R (Additional file 1: Table S2). The plasmids were transferred into *S. albus* J1074 for further analysis. The strains were cultivated in SGG medium. After a certain period of growth, 1 mL of culture was harvested and washed once with distilled water, and β-glucuronidase activity was measured as described in [45]. The enzymatic activity was normalized to the dry weight of the biomass of corresponding culture.

### Isolation of pamamycins

Pamamycins were extracted from the culture broth with ethyl acetate and from the biomass with an acetone and methanol mixture (ratio, 1:1) for 1 h with shaking at 180 rpm (Laboshake, Gerhardt GmBH, Germany). Extracts were evaporated, dissolved in 1 mL methanol, combined together and again evaporated. The final extracts were dissolved in a 250 µL mixture of methanol and DMSO (1:1), centrifuged (19,000 × *g*, 10 min, 4 ℃) and subjected to LC‒MS analysis.

### HPLC‒MS analysis of pamamycin production

Pamamycin extracts were analyzed by LC‒MS (Dionex Ultimate 3000, Thermo Fisher Scientific, USA; AmaZon Speed ETD, Bruker, Germany). Samples were eluted with solvent A (90 mM ammonium formate in water) and solvent B (100:20 acetonitrile/100 mM ammonium formate) in a multistep gradient: 0.2 min 20% B, 20% B to 97% B in 2.8 min, 97% B to 100% B in 7 min, 1 min at 100%, 100% B to 20% B in 1 min, and 3 min equilibration at 20% B. A Waters BEH C18 column (100 mm × 2.1 mm, 1.7 µm) was used with a flow rate of 0.55 mL/min and a column chamber temperature of 45 ℃. The data were analyzed with the software Compass DataAnalysis (Bruker, Germany).

Samples from the recombinant strains were additionally analyzed on a maXis 4G hr-ToF ultrahigh-resolution mass spectrometer using the Apollo II ESI source (Bruker Daltonics, Germany). Extracts were separated on a UPLC system (Dionex Ultimate 3000, Thermo Fisher Scientific, USA) with a Waters BEH C18 column (100 mm × 2.1 mm, 1.7 μm, column, temperature 45 ℃). Buffers A (Milli-Q, 0.1% formic acid) and B (acetonitrile, 0.1% formic acid) and the gradient of solvent B from 5 to 95% in 18 min were used. The flow rate was set to 0.5 mL/min.

### RNA sequencing and data analysis

Whole-transcriptome sequencing was performed by CeBiTec (Beliefeld, Germany). *S. albus* J1074 strains carrying the R2, R2-67, R2-73 or R2-100 cosmids were grown in SGG media, and biomass was collected at 50 h of cultivation. Total RNA was isolated using the peqGOLD TriFast reagent (PEQLAB, Germany) including DNase treatment; rRNA was depleted with the Ribo-Zero Bacteria kit (Illumina, USA); and total RNA was used to synthesize cDNA with the TruSeq Stranded mRNA Library Prep Kit from Illumina (San Diego, CA, United States). The cDNA was sequenced on the HiSeq 1500 platform (Illumina, USA), resulting in the production of 443.3 Mbp of 75 bp-long sequences. Sequences were end-trimmed using Geneious, version 8.1.7 (Biomatters Ltd, New Zealand), and mapped to the *S. albus* J1074 genome with the R2 cosmid sequence inserted into the øC31 actinophage *attB* site with the ReadXplorer v.2 analysis tool (https://bio.tools/readxplorer) [17].

### Isolation and purification of pamamycins

Preparative pamamycin production was performed by scaling up the procedure described above. A total of 10 L of SSG medium (200 flasks by 50 mL) was used. Metabolites were extracted as described. The crude extract was used for pamamycin purification by a two-step chromatography approach. First, size exclusion chromatography was performed: solid phase—cross-linked dextran polymer beads (LH-20, GE Healthcare Bio-Science AB) packed into a 63 cm long column; mobile phase—100% methanol with a total volume of 0.7 L and a flow rate of 1 mL/min. Fractions were collected every 12 min and screened for the presence of pamamycins.

The fractions containing pamamycins were pooled together, evaporated, and dissolved in methanol. Pamamycins were purified on an Agilent Technologies 1260 Infinity semipreparative HPLC using a Synergi column TM 4 µm Fusion RP 80 Å (250 × 10 mm). Samples were eluted with solvents A (Milli-Q, 0.1% formic acid) and B (acetonitrile, 0.1% formic acid) and a multistep gradient: 2 min 5% B, 5% B to 60% B in 4 min, 60% B to 95% B in 20 min, 2 min at 95%, 95% B to 5% B in 1 min, and 3 min equilibration at 5% B. The fractions were collected every 30s and analyzed for purity of compounds with LC‒MS as described above. The fractions containing individual compounds of high purity were evaporated and dissolved in methanol. Purified compounds were used for NMR structure elucidation and activity testing.

### NMR analysis of pamamycins

NMR spectra were acquired at 298 K on a Bruker Ascend 500 or a Bruker Ascend 700 NMR spectrometer (Bruker, Germany). Both spectrometers were equipped with a 5 mm TXI cryoprobe. CDCl_3_ was used as the solvent. The chemical shifts (δ) were reported in parts per million (ppm) relative to CHCl_3_ at 7.26 ppm for the proton spectra and to CDCl_3_ at 77.0 ppm for the carbon spectra. 2D NMR ^1^H-^1^H-COSY, edited-HSQC, HSQC-TOCSY and HMBC were recorded using the standard pulse programs from TOPSPIN v.3.6 software. For homopamamycin 677A, 1D selective TOCSY was measured with mixing times of 75 and 125 ms.

### Biological activity tests

Antimicrobial activity studies were performed against the following test strains: *Acinetobacter baumannii* DSM-30008, *Escherichia* *coli* JW0451-2 (ΔacrB), *E. coli* BW25113, *Staphylococcus aureus* Newman, *Mycobacterium smegmatis* mc2155, *Pseudomonas aeruginosa* PA14, *Bacillus subtilis* DSM-10, *Citrobacter freundii* DSM-30039, *Mucor hiemalis* DSM-2656, *Candida albicans* DSM-1665, *Cryptococcus neoformans* DSM-11959 and *Pichia anomala* DSM-6766. Minimum inhibitory concentrations (MICs) were determined according to standard procedures [1]. Serial dilutions of pamamycins, dissolved in DMSO, ranging from 1 to 128 µM were prepared in sterile 96-well plates, and the bacterial suspension was added. Growth inhibition was assessed after incubation for 16–48 h at 30 or 37 ℃ depending on the test culture.

Cytotoxicity was tested with the human cancer cell lines KB-3.1 (ACC-158) and HepG2 (ACC-180). The cell lines were cultivated according to the collection recommendations. Serial dilutions of pamamycins were added to the cell lines and incubated for 3 days. Viability was determined by photometric measurement of MTT (thiazolyl blue tetrazolium bromide) compared to the DMSO control, and the average value was used to determine the IC50 (standard deviation < 10%).

The maximum tolerated concentration (MTC) was studied on zebrafish as described in [41]. Briefly, 10 zebrafish eggs, either 1- or 3-days post-fertilization, were mixed with 10 µM pamamycins and monitored for 1 day via a LEICA M205 FA stereo microscope (Leica Mikrosysteme Vertrieb GmbH, Wetzlar, Germany).

Herbicidal test was performed on *Agrostis stolonifera* as described in Rodríguez et al. [42]. Serial dilutions of pamamycins dissolved in DMSO (2.5, 5, 10, 20 and 40 µM) were tested. The seeds were incubated for 6 days at room temperature in the light box (Osram Fluora lamp), and the germinated seeds were counted.

### Supplementary Information

**Additional file 1: Table S1.** Bacterial strains and plasmids. **Table S2.** Oligonucleotides used in this study. **Table S3.** Schematic overview of pamamycin production by engineered R2 constructs expressed in *S. albus* J1074 pTOS-P21pamW. **Table S4.** Sequences of the semisynthetic promoters (Sequence (5’→3’). **Table S5.** Results of antibacterial tests of different pamamycins. **Table S6.** Results of activity tests of pamamycins with different molecular weight against cell lines. **Table S7.** Results of activity tests of pamamycins with different molecular weight against zebra fish embryos. **Table S8.** Results of activity tests of pamamycins with different molecular weight against *Agrostis stolonifera*. **Fig. S1.** High resolution MS-chromatograms of *S. albus* J1074 R2-73. Pamamycin derivatives are indicated by their molecular weight. **Fig. S2.** Biomass of *S. albus* J1074 P21pamW strains containing different R2 cosmids over time: Blue: R2; Red: R2-67; green R2-73; R2-100; light blue: dry biomass of the medium. **Fig. S3.** Multiple sequence alignment of ErmEp1 to different synthetic promoters based on the -10 and -35 region of ErmEp1. Despite P21 all promoters were obtained in this research and P21 serves as an example for a very a promoter with strong activity. **Table S’1–Table S’2 and Fig. S’1–Fig. S’7.** Complete set of NMR data for Pamamycin-635 G. **Table S’3–Table S’4 and Fig. S’8–Fig. S’15.** Complete set of NMR data for Pamamycin-663 A. **Table S’5–Table S’6 and Fig. S’16–Fig. S’30.** Complete set of NMR data for Pamamycin-677 A.

## Data Availability

All data generated and analyzed during this study are included in this manuscript and in its Supplementary information file.

## Abbreviations

pam: Pamamycin
BGC: Biosynthetic gene cluster
AMR: Antimicrobial resistance
RBS: Ribosome binding site
MIC: Minimal inhibitory concentration
ACP: Acyl carrier protein
SGG: Starch glucose glycerol medium
Hyg^R^: Hygromycin resistance
