# Supplementary material for: Discovery and overproduction of novel highly bioactive pamamycins through transcriptional engineering of the biosynthetic gene cluster
Source: Microb Cell Fact. 2023 Nov 14;22:233. doi: 10.1186/s12934-023-02231-x (PMC10644645; doi:10.1186/s12934-023-02231-x)
Supplement: Supplementary file 1 — Additional file 1: Table S1. Bacterial strains and plasmids. Table S2. Oligonucleotides used in this study. Table S3. Schematic overview of pamamycin production by engineered R2 constructs expressed in S. albus J1074 pTOS-P21pamW. Table S4. Sequences of the semisynthetic promoters (Sequence (5’→3’). Table S5. Results of antibacterial tests of different pamamycins. Table S6. Results of activity tests of pamamycins with different molecular weight against cell lines. Table S7. Results of activity tests of pamamycins with different molecular weight against zebra fish embryos. Table S8. Results of activity tests of pamamycins with different molecular weight against Agrostis stolonifera. Fig. S1. High resolution MS-chromatograms of S. albus J1074 R2-73. Pamamycin derivatives are indicated by their molecular weight. Fig. S2. Biomass of S. albus J1074 P21pamW strains containing different R2 cosmids over time: Blue: R2; Red: R2-67; green R2-73; R2-100; light blue: dry biomass of the medium. Fig. S3. Multiple sequence alignment of ErmEp1 to different synthetic promoters based on the -10 and -35 region of ErmEp1. Despite P21 all promoters were obtained in this research and P21 serves as an example for a very a promoter with strong activity. Table S’1–Table S’2 and Fig. S’1–Fig. S’7. Complete set of NMR data for Pamamycin-635 G. Table S’3–Table S’4 and Fig. S’8–Fig. S’15. Complete set of NMR data for Pamamycin-663 A. Table S’5–Table S’6 and Fig. S’16–Fig. S’30. Complete set of NMR data for Pamamycin-677 A. [file 12934_2023_2231_MOESM1_ESM.pdf]

Supplementary materials

Discovery of novel highly bioactive pamamycins through transcriptional engineering of the biosynthetic gene cluster

Nikolas Eckert, Liliya Horbal, Yuriy Rebets, Josef Zapp, Jennifer Herrmann, Tobias Busche, Rolf Müller, Jörn Kalinowski, Andriy Luzhetskyy

Table S1. Bacterial strains and plasmids

**Bacterial strains**	**Description**	**Source**
E. coli ET12567 (pUB307)	Conjugative transfer of DNA	[1]
E. coli GB05-redCC	Derivative of GB2005 containing Red/ET mediating plasmid pSC101-BAD-gbaA	[2]
E. coli GB2005	*E. coli* strain used in basic cloning procedures	[3]
S. albus J1074	Parental strain	[4]
S. albus J1074 R2	*S. albus* J1074 containing the R2 cosmid with the native *pam* BGC, AmR	This study
S. albus J1074 P21pamW	*S. albus* J1074 with overexpression of pamamycin transporter gene *pamW,* markerless	This study
S. albus J1074 P21pamW/R2	*S. albus* J1074 pTOS-P21pamW containing the R2 cosmid, AmR	This study
S. albus J1074 P21pamW/R2 library	*S. albus* J1074 pTOS-P21pamW with derivatives of the R2 cluster, AmR	This study
S. albus J1074 Del14	*S. albus* J1074 derived heterologous expression host	[5]
S. albus J1074 pGUS	*S. albus* J1074 strain with the pGUS plasmid	[6]
S. albus J1074 pGUS-p67up/p73up/p100up/pR2up	*S. albus* J1074 strain with the pGUS plasmid with different upstream promoters of R2 constructs cloned in front of the reporter gene, AmR	This study
S. albus J1074 pGUS-p67down/p73down/p100down/pR2down	*S. albus* J1074 strain with the pGUS plasmid with different downstream promoters of R2 constructs cloned in front of the reporter gene	This study
S. albus J1074 R2-p73up	*S. albus* J1074 strain with the R2 cluster and the semisynthetic promoter p73up	This study
S. albus J1074 R2-p73down	*S. albus* J1074 strain with the R2 cluster and the semisynthetic promoter p73down	This study

**Plasmids/Cosmids**	**Description**	**Source**
pTOS	Apramycin resistance (Am^R), VWB-based integrative vector	[7]
pTOS-P21pamW	pTOS derivative containing *pamW* gene under P21 promoter	This work
pUWLH	pIJ101 replicon based replicative vector, HygR	[8]
pUWLHpamS	pUWLH with cloned *pamS* gene	This work
pUWLHpamW	pUWLH with cloned *pamW* gene	This work
R2	Derivative of pOJ436 containing the cluster for pamamycin biosynthesis	[9]
R2 mutant library	R2 cosmids, containing a Hyg^R cassette flanked by 2 random promoters between the *pamF* and *pamA* genes	This work
R2- pamAp73	R2 cosmid containing a Hyg^R cassette with the downstream oriented promoter of the R2 mutant R2-73	This work
R2- pamFp73	R2 cosmid containing a Hyg^R cassette with the upstream oriented promoter of the R2 mutant R2-73	This work
pGUS	pSET152 derivative with promoterless *gusA* gene	[10]
pGUS-pR2Up/p67Up/p73Up/p100Up	Derivative of pGUS containing the upstream promoter of the R2/R2-67/R2-73 or R2-100 cosmids cloned in front of the *gusA* reporter gene	This work
pGUS-pR2Down/p67Down/p73Down/p100Down	Derivative of pGUS containing the downstream promoter of the R2/R2-67/R2-73 or R2-100 cosmids cloned in front of the *gusA* reporter gene	This work

Table S2. Oligonucleotides used in this study

Pair N.	Name	Sequence (5’→3’)	Purpose
1	pamWFEcoRV<br>pamWRPstI	GATATCAATCCCGAAGGAGGCCAGTC<br>CTGCAGCGGCTGCTTCCTGTGGTCA	Cloning of *pamW* gene into pUWL-Hyg vector
2	pamSFEcoRV<br>pamSRPstI	GATATCATTCAGGGTGGGCAGAAGCA<br>CTGCAGCCCCGTCCGTGCACGTCAT	Cloning of *pamS* gene into pUWL-Hyg vector
3	PamWIntro-F<br>PamWIntro-R	ACGATCTGTTCACGGCTCAGCGGCTGCCTCGCCGGCCGCTGCTTC<br>GGCTGCTTCCTGTGGTCATGTGCGGGCTCTAACACGTCCTAGTAT	Amplification of *pamW*
4	pTOS-F<br>pTOS-R	AAAATCCTGTATATCGTGCG<br>ACTCAGACTCACTGAGGCTC	Primer pair for checking for introduction of *pamW*
5	PamPrandomHyg^{R}-F<br><br><br>PamPrandomHyg^{R}-R	GATCAGTGCGTCGCGACCGGGATCCTGCTGCTGATGCGCCATGCGAC<br>TTCCCTCCTTCANNNNNNATCCTANNNNNNNNNNNNNNNNNNNNAG<br>CCNNNNNNCGCGGGATGTATCAGGCGCC<br>CCCCGCGGTTTTTCCGTGCAGCGGATTCAGCCTGTGCAGCCATAACGA<br>TTCCTCCGATANNNNNNATCCTANNNNNNNNNNNNNNNNNNNNAGC<br>CNNNNNAAATACTTGACATATCACTG	Primer pair for homologous recombination and introduction of degenerated promoter sequences
6	Hyg^{R}-Seq-F<br>Hyg^{R}-Seq-R	AGGCTCGCGTAGGAATCATCC<br>AACTGCATCTCAACGCCTTCC	Primer pair for partially sequencing of the introduced promoters attached to the Hyg^{R} cassette
7	R2-pamAp73-F<br><br>R2-pamAp73-R	GGATTCTCCCGGGTACCGGTGGGCCGATGTCCCTCGAATTCCCGGCTC<br>AGGCGCCGGGGGCGGTGT<br>CCCCGCGGTTTTTCCGTGCAGCGGATTCAGCCTGTGCAGCCATAACGA<br>TTCCTCCGATACCTCGTATCCTATATGAATATCACGGGGATGAGCCGG<br>GATAAATACTTGACATATCACTG	Primer for amplification of the downstream p73Down promoter and introduction with an Hyg^{R} cassette into R2
8	R2-pamFp73-F<br><br><br>R2-pamFp73-R	TGCGTCGCGACCGGGATCCTGCTGCTGATGCGCCATGCGACTTCCCTC<br>CTTCACAACTCATCCTAACTCATCGCTAGCCGTTGGAGCCGGTACTCGC<br>GGGATGTATCAGGCGCC<br>CGACCCGCACCGCTCTAGCGTCGGCGGCCTGTCGACAGCGGCACGCA<br>AATACTTGACATATCACTG	Primer for amplification of the upstream p73Up promoter and introduction with an Hyg^{R} cassette into R2
9	R2-pamFp67-GU-R<br><br><br>R2-GU-F-all	AAAAGGTACCGCGACTTCCCTCCTTCAGCTGGTATCCTAATTTCTGGGT<br>TTTTCTTCGAGCCCGGTGAAAATACTTGACATATCACTGT<br>AAAATCTAGATCAGGCGCCGGGGGCGGTGT	Primer for the amplification of the P67-Up promoter and a Hyg^{R} cassette for the cloning into pGUS with restriction sites *Spe*I and *Xba*I
10	R2-pamAp67-GU-R<br>R2-GU-F-all	AAAAGGTACCAACGATTCCTCCGATAGCGA<br>AAAATCTAGATCAGGCGCCGGGGGCGGTGT	Primer for the amplification of the P67Down promoter and a Hyg^{R} cassette for the cloning into pGUS with restriction sites *Spe*I and *Xba*I
11	R2-pamFp73-GU-R<br>R2-GU-F-all	AAAAGGTACCGCGACTTCCCTCCTTCACAACTCATCCTAACTCATCGCT<br>AGCCGTTGGAGCCGGTACTAAATACTTGACATATCACTGT<br>AAAATCTAGATCAGGCGCCGGGGGCGGTGT	Primer for the amplification of the p73Up promoter and a Hyg^{R} cassette for the cloning into pGUS with restriction sites *Spe*I and *Xba*I
12	R2-pamAp73-GU-R<br>R2-GU-F-all	AAAAGGTACCAACGATTCCTCCGATACCTC<br>AAAATCTAGATCAGGCGCCGGGGGCGGTGT	Primer for the amplification of the p73Down promoter and a Hyg^{R} cassette for the cloning into pGUS with restriction sites *Spe*I and *Xba*I
13	R2-pamFp100-GU-R<br>R2-GU-F-all	AAAAGGTACCGCGACTTCCCTCCTTCATCTGCGATCCTAGCACGAAAC<br>AGGGTATGACAGCCTGTGTTAAATACTTGACATATCACTGT<br>AAAATCTAGATCAGGCGCCGGGGGCGGTGT	Primer for the amplification of the P100Up promoter and a Hyg^{R} cassette for the cloning into pGUS with restriction sites *Spe*I and *Xba*I
14	R2-pamAp100-GU-R<br>R2-GU-F-all	AAAAGGTACCAACGATTCCTCCGATAGAGG<br>AAAATCTAGATCAGGCGCCGGGGGCGGTGT	Primer for the amplification of the P100Down promoter and a Hyg^{R} cassette for the cloning into pGUS with restriction sites *Spe*I and *Xba*I
15	R2-pamA-GU-R<br>R2-GU-F-all	AAAAGGTACCACGGGAAAGCTATCCGCCGG<br>AAAATCTAGATCAGGCGCCGGGGGCGGTGT	Primer for the amplification of the native downstream promoter and a Hyg^{R} cassette for the cloning into pGUS with restriction sites *Spe*I and *Xba*I
16	R2-PL-Nat-R<br>R2-PL-Nat-F	AAAAGGTACCGAGACTCCCTGTGTTGCGTG<br>TATGTGAATCACAGTGATATGTCAAGTATTTGCCGGGAATTCGAGGGAC[illegible]	Primer for the amplification of the native upstream promoter for the cloning into pGUS with restriction site *Spe*I
17	R2-PL-Nat-HygR-R<br>R2-GU-F-all	GGTGGGCCGATGTCCCTCGAATTCCCGGCAAATACTTGACATATCACTGT<br>AAAATCTAGATCAGGCGCCGGGGGCGGTGT	Primer for the amplification of a Hyg^{R} cassette for the cloning into pGUS with restriction site *Xba*I
18	R2-pamF-GU-R<br>R2-GU-F-all	AAAAGGTACCGAGACTCCCTGTGTTGCGTG<br>AAAATCTAGATCAGGCGCCGGGGGCGGTGT	Primer for the amplification of the native upstream promoter and a Hyg^{R} cassette for the cloning into pGUS with restriction sites *Spe*I and *Xba*I

Table S3. Schematic overview of pamamycin production by engineered R2 constructs expressed in *S. albus* J1074 pTOS-P21pamW.

Name	Production	Production	Name
R2-1			R2-28
R2-2			R2-29
R2-3			R2-30
R2-4			R2-31
R2-5			R2-32
R2-6			R2-33
R2-7			R2-34
R2-8			R2-35
R2-9			R2-36
R2-10			R2-37
R2-11			R2-38
R2-12			R2-39
R2-13			R2-40
R2-14			R2-41
R2-15			R2-42
R2-16			R2-43
R2-17			R2-44
R2-18			R2-45
R2-19			R2-46
R2-20			R2-47
R2-21			R2-48
R2-22			R2-49
R2-23			R2-50
R2-24			R2-51
R2-25			R2-52
R2-26			R2-53
R2-27			

Name	Production	Production	Name
R2-54			R2-81
R2-55			R2-82
R2-56			R2-83
R2-57			R2-84
R2-58			R2-85
R2-59			R2-86
R2-60			R2-87
R2-61			R2-88
R2-62			R2-89
R2-63			R2-90
R2-64			R2-91
R2-65			R2-92
R2-66			R2-93
R2-67			R2-94
R2-68			R2-95
R2-69			R2-96
R2-70			R2-97
R2-71			R2-98
R2-72			R2-99
R2-73			R2-100
R2-74			R2-101
R2-75			R2-102
R2-76			R2-103
R2-77			R2-104
R2-78			R2-105
R2-79			R2-106
R2-80			

Legend: red – strains producing no pamamycin or with the yield significantly lower than in the control R2 carrying strain; green – strains with the pamamycin production on par or higher than that of *S. albus* J1074 P21pamW/R2.

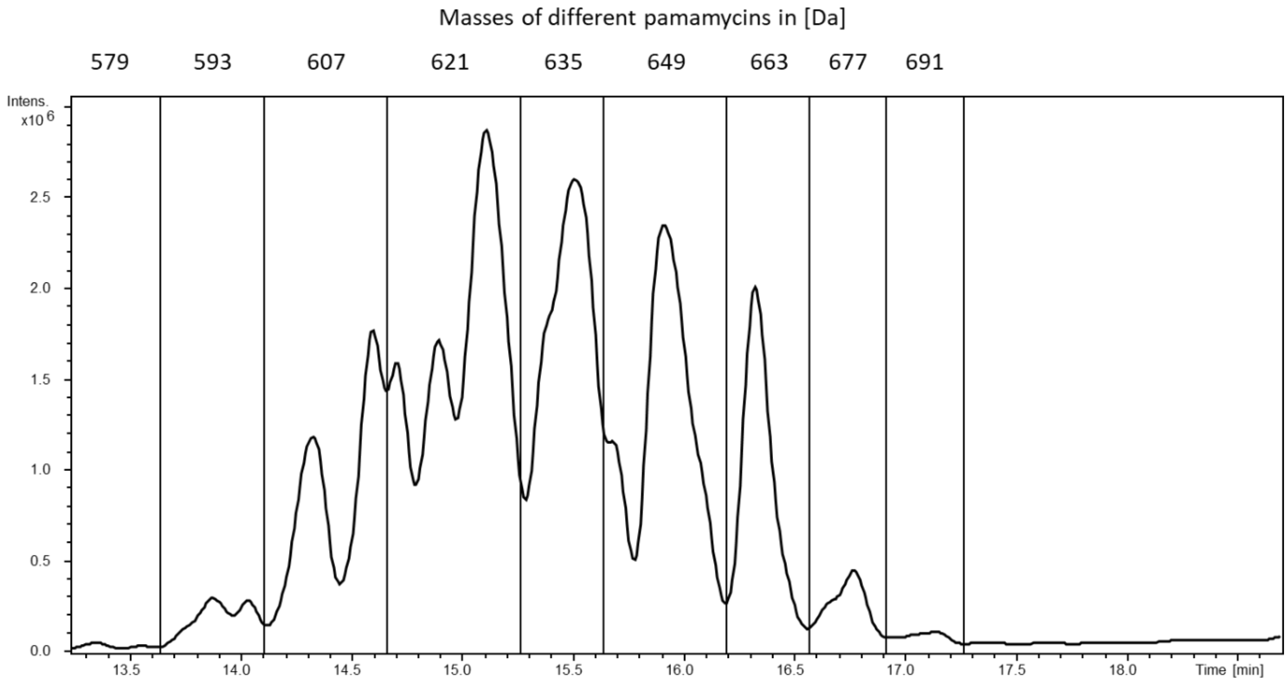

Fig. S1. High resolution MS-chromatograms of *S. albus* J1074 R2-73. Pamamycin derivatives are indicated by their molecular weight.

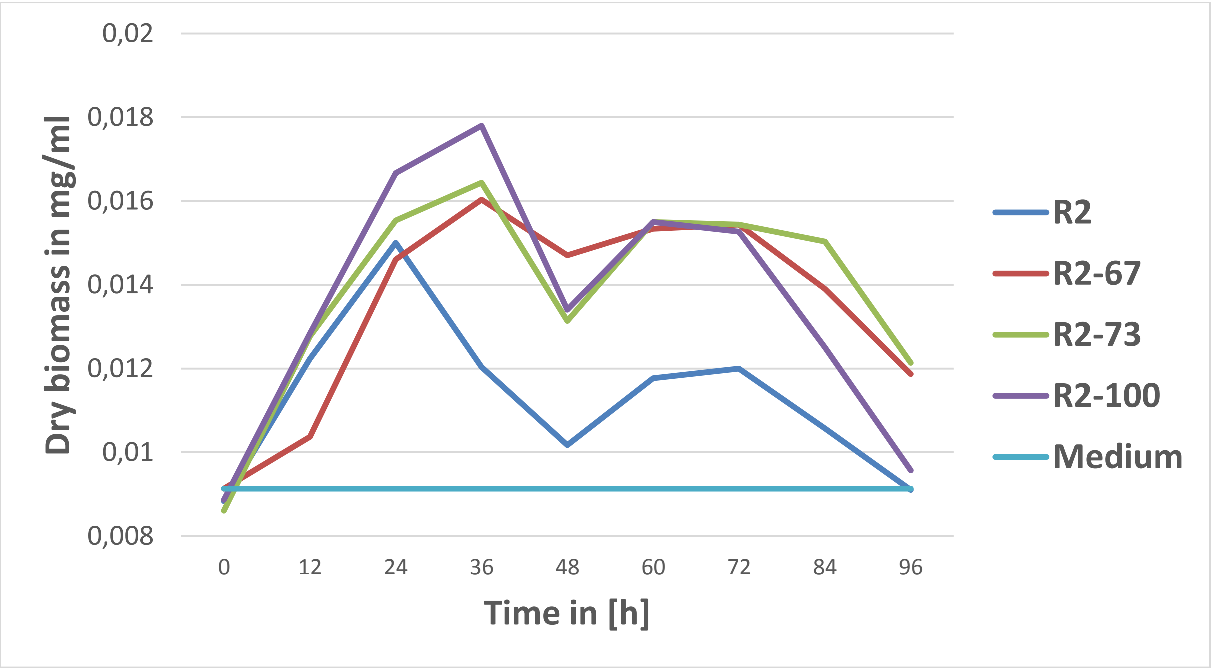

Fig. S2. Biomass of *S. albus* J1074 P21pamW strains containing different R2 cosmids over time: Blue: R2; Red: R2-67; green R2-73; R2-100; light blue: dry biomass of the medium.

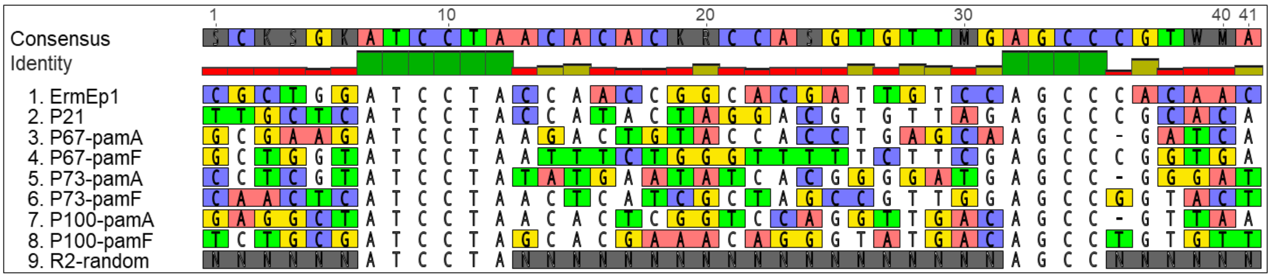

Fig. S3. Multiple sequence alignment of ErmEp1 to different synthetic promoters based on the -10 and -35 region of ErmEp1. Despite P21 all promoters were obtained in this research and P21 serves as an example for a very a promoter with strong activity.

Table S4. Sequences of the semisynthetic promoters (Sequence (5'→3').

Promoter Name	RBS	Conserved	Random 5' UTR	-10 Region	Random spacer	-35 Region	Random
Promoter	CCTCC	TTCA	NNNNNN	ATCCTA	NNNNNNNNNNNNNNNNNN	AGCC	NNNNNN
ErmE			CGCTGG	ATCCTA	CCAACCGGCACGATTGTCC	AGCC	CACAAC
pamF67	CCTCC	TTCA	GCTGGT	ATCCTA	ATTTCTGGGTTTTTCTTCG	AGCC	CGGTGA
pamF73	CCTCC	TTCA	CAACTC	ATCCTA	ACTCATCGCTAGCCGTTGG	AGCC	GGTACT
pamF 100	CCTCC	TTCA	TCTGCG	ATCCTA	GCACGAAACAGGGTATGAC	AGCC	TGTGTT
pamA67	CCTCC	GATA	GCGAAG	ATCCTA	AGACTGTACCACCTGAGCA	AGCC	GATCA
pamA73	CCTCC	GATA	CCTCGT	ATCCTA	TATGAATATCACGGGGATG	AGCC	GGGAT
pamA100	CCTCC	GATA	GAGGCT	ATCCTA	ACACTCGGTCCAGGTTGAC	AGCC	GTTAA

Table S5. Results of antibacterial tests of different pamamycins.

Indicator strain	MIC [µM]		
	Pam-607	Pam-649A	Pam-663A
E. coli BW25113	> 128	32	32
E. coli ΔacrB	32	32	32
P. aeruginosa PA14	> 128	128	128
P. aeruginosa PA14 ΔmexAB	> 128	64	128
B. subtilis DSM-10	8	2	2
E. faecium DSM-20477	16	8	4
M. smegmatis mc^2155	16	8	8
S. aureus Newman	8	2	1
S. aureus N315 (MRSA)	16	2	1
S. pneumoniae DSM-20566	4-8	1	1

Table S6. Results of activity tests of pamamycins with different molecular weight against cell lines

Cell line	IC_{50} [µM]			
	Pam-607	Pam-649	Pam-663	Doxorubicin
KB-3.1	0.48	0.06	0.02	0.00034
HepG2	0.62	0.01	0.002	0.00029

Table S7. Results of activity tests of pamamycins with different molecular weight against zebra fish embryos

Danio rerio line and age	MTC [µM]		
	Pam-607	Pam-649	Pam-663
TL; 1 dpf (embryos)	< 10	< 10	< 10
TL; 3 dpf (larvae)	< 10	10	< 10

Table S8. Results of activity tests of pamamycins with different molecular weight against *Agrostis stolonifera*

Agrostis stolonifera	Different pamamycins and their Concentration [µM] / Number of grown plants		
Dilution	Pam-607	Pam-649A	Pam-663A
0	41.2 / 0	38.5 / 0	37.7 / 0
1	20.6 / 2	19.25 / 3	18.85 / 4
2	10.3 / 5	9.625 / >5	9.425 / 5
3	5.15 / >5	4.8125 / >5	4.7125 / >5
4	2.575 / >5	2.40625 / >5	2.35625 / >5

Table S’1. NMR data (500 MHz, $CDCl_3$) for Pamamycin-635 G

	δ_C m	δ_{CH} (J in Hz)
**1**	172.80 C	-------------------
**2**	54.86 CH	2.13 m
**3**	81.77 CH	3.61 td (10.5, 5.0)
**4**	29.55 CH_2	1.29 m and 1.93 m
**5**	30.59 CH_2	1.47 m and 2.03 m
**6**	74.47 CH	3.79 m
**7**	38.90 CH_2	1.44 m and 2.13 m
**8**	70.06 CH	5.32 ddd (12.5, 3.0, 1.5)
**9**	49.33 CH	1.30 m
**10**	79.65 CH	3.42 dt (10.5, 7.0)
**11**	29.03 CH_2	1.58 m and 2.05 m
**12**	30.89 CH_2	1.52 m and 2.06 m
**13**	78.65 CH	3.80 br m
**14**	33.73 CH_2	1.73 m and 2.18 m
**15**	67.91 CH	3.40 m
**16**	28.72 CH_2	1.46 m and 1.72 m
**17**	19.56 CH_2	1.40 m and 1.50 m
**18**	13.86 CH_3	0.99 t (7.0, 3H)
**1´**	173.44 C	-------------------
**2´**	49.89 CH_2	2.34 dt (11.3, 2.5)
**3´**	79.15 CH	3.89 ddd (9.3, 7.5, 2.5)
**4´**	27.35 CH_2	1.70 m and 1.83 m
**5´**	31.44 CH_2	1.36 m and 1.97 m
**6´**	74.38 CH	3.72 m
**7´**	38.26 CH_2	1.83 m and 1.58 m
**8´**	71.38 CH	4.80 m
**9´**	36.62 CH_2	1.47 m and 1.62 m
**10´**	18.02 CH_2	1.30 m (2H)
**11´**	13.96 CH_3	0.89 t (7.5, 3H)
**2-Et**	22.04 CH_2	1.43 m and 1.54 m
	11.74 CH_3	0.89 t (7.5, 3H)
**9-Et**	18.92 CH_2	1.21 m and 1.52 m
	13.81 CH_3	0.95 (3H)
**15-N(Me)$_2$**	36.31 CH_{3a}	2.86 d (5.0, 3H)
	43.33 CH_{3b}	3.08 d (5.0, 3H)
**2´-Et**	16.02 CH_2	1.38 m and 1.75 m
	12.69 CH_3	0.85 t (7.5, 3H)
**NH**		8.87 br s

Table S’2. HMBC Key correlations for Pamamycin-635 G

Position	HMBC correlations
H-2	C-1, C-3, C-2-Et
H-3	C-1, C-2, C-4, C-5, C-2-Et
H-6	C-3, C-8
H-8	C-6, C-7, C-9, C-10, C-1´, C-9-Et
H-10	C-8, C-9, C-9- C-2-Et
H-13	C-11, C-15
H-15	C-12, C-14, C-17, 15-N-CH_{3a}
H-18	C-16, C-17
H-2´	C-1´, C-3´, C-2´-Et
H-3´	C-1´, C-2´, C-4´, C-5´, C-2´-Et
H-6´	C-3´, C-8´
H-8´	C-1, C-6´, C-7´, C-10´
H-11´	C-9´, C-10´
CH_3-2 (from Et)	C-2
CH_3-9 (from Et)	C-9
CH_3-2´(from Et)	C-2´
CH_{3a}-N	C-15, 15-N-CH_{3b}
CH_{3b}-N	C-15, 15-N-CH_{3a}

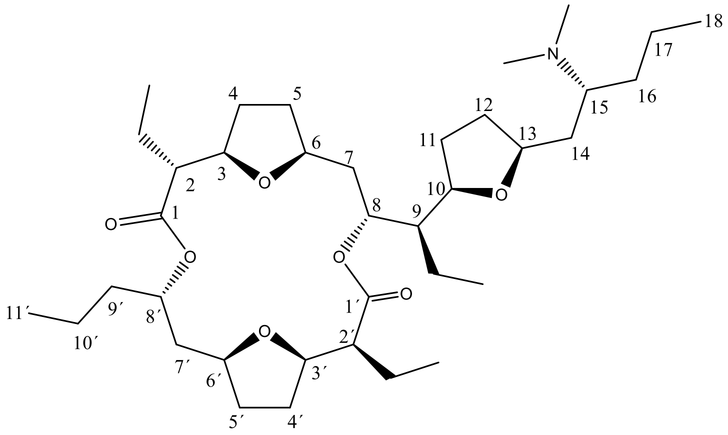

Fig. S’1. Structure of Pamamycin-635 G

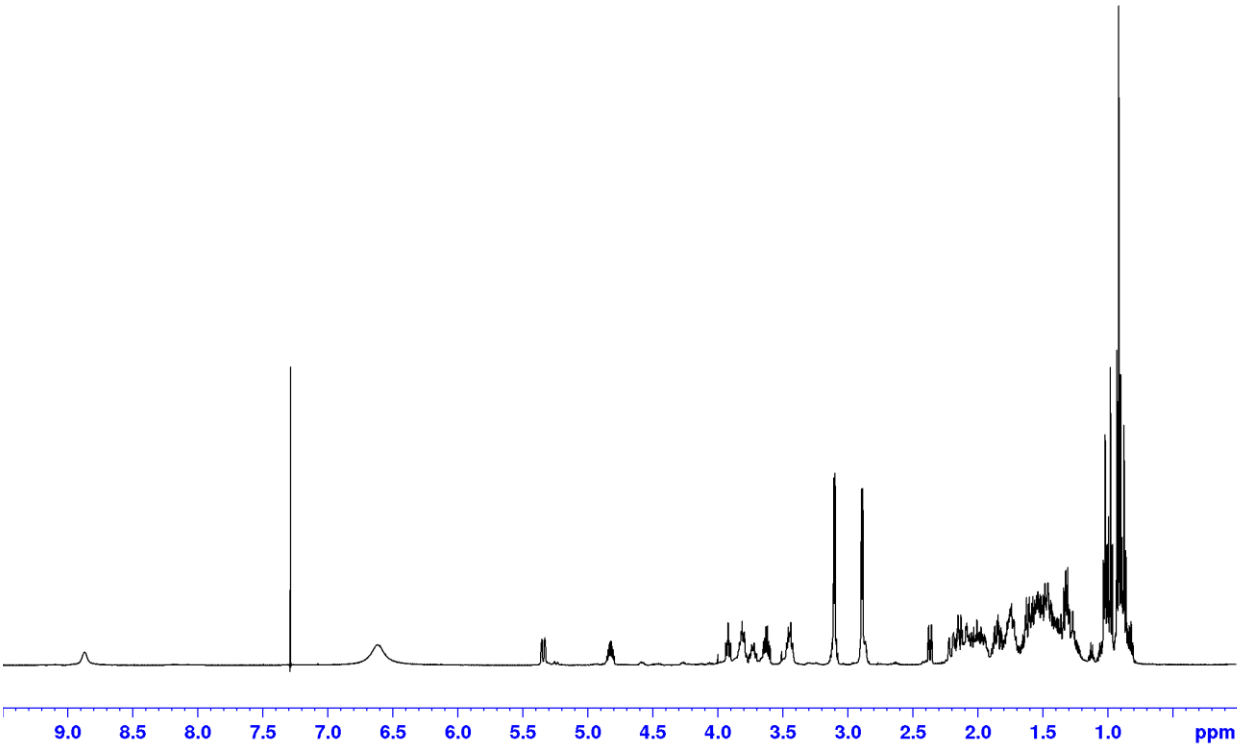

Fig. S'3. ^{1}H NMR spectrum (500 MHz, $CDCl_3$) of Pamamycin-635 G

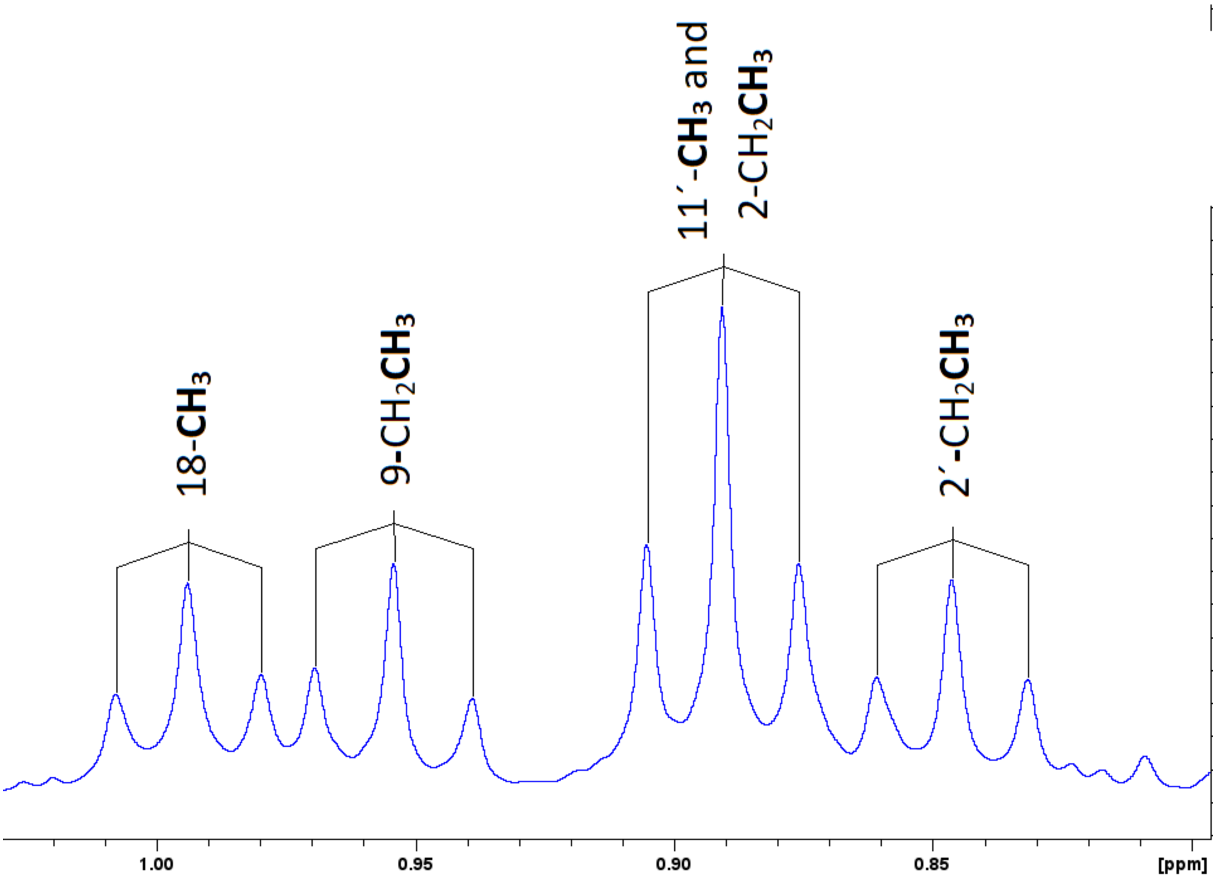

Fig. S'3. ^{1}H NMR spectrum (500 MHz, $CDCl_3$) of Pamamycin-635 G, methyl region

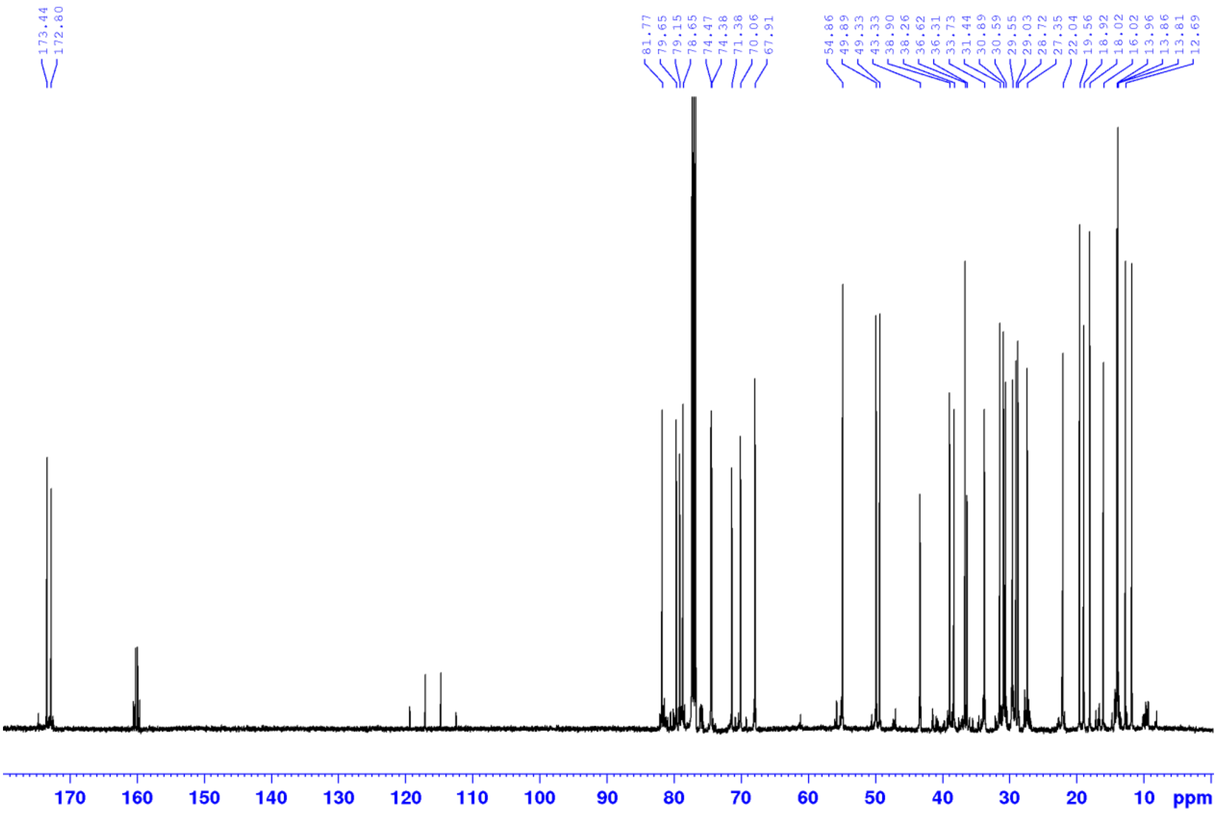

Fig. S'4. ^{13}C NMR spectrum (125 MHz, $CDCl_3$) of Pamamycin-635G

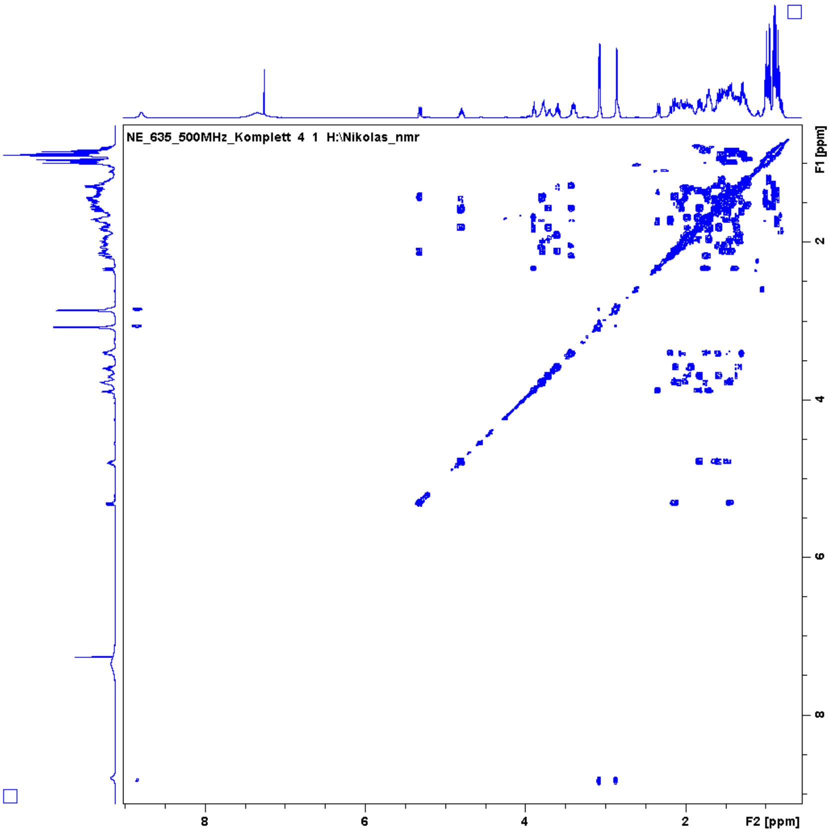

Fig. S'4. ^{1}H-^{1}H COSY spectrum (CDCl3) of Pamamycin-635 G

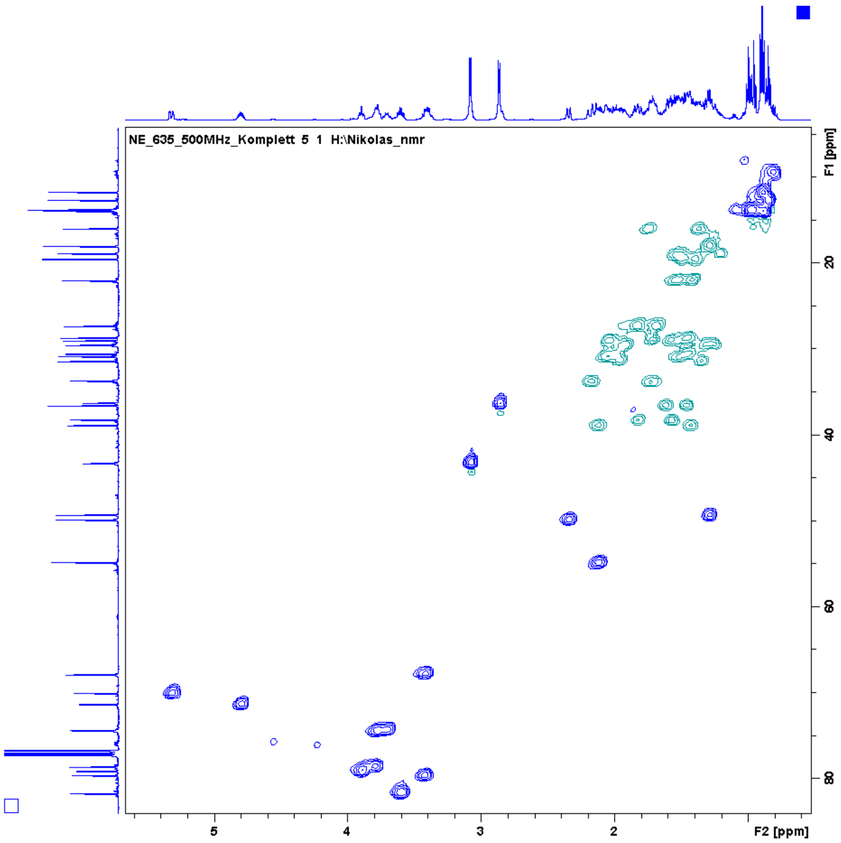

Fig. S'5. Edited HSQC spectrum (CDCl3) of Pamamycin-635 G

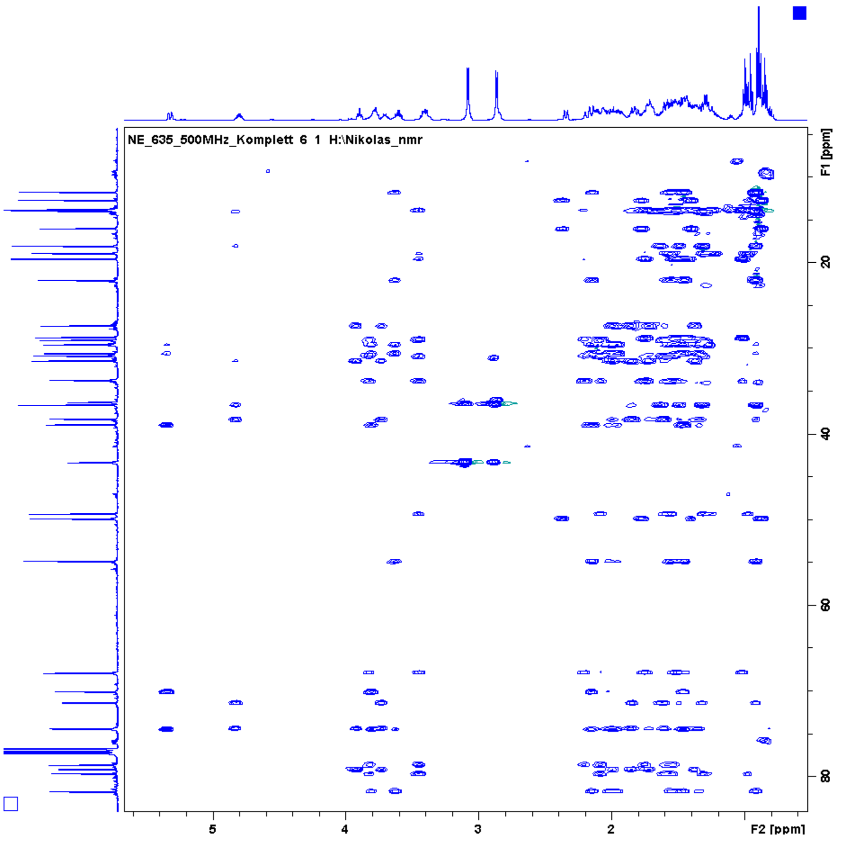

Fig. S'6. HSQC-TOCSY spectrum (CDCl3) of Pamamycin-635 G

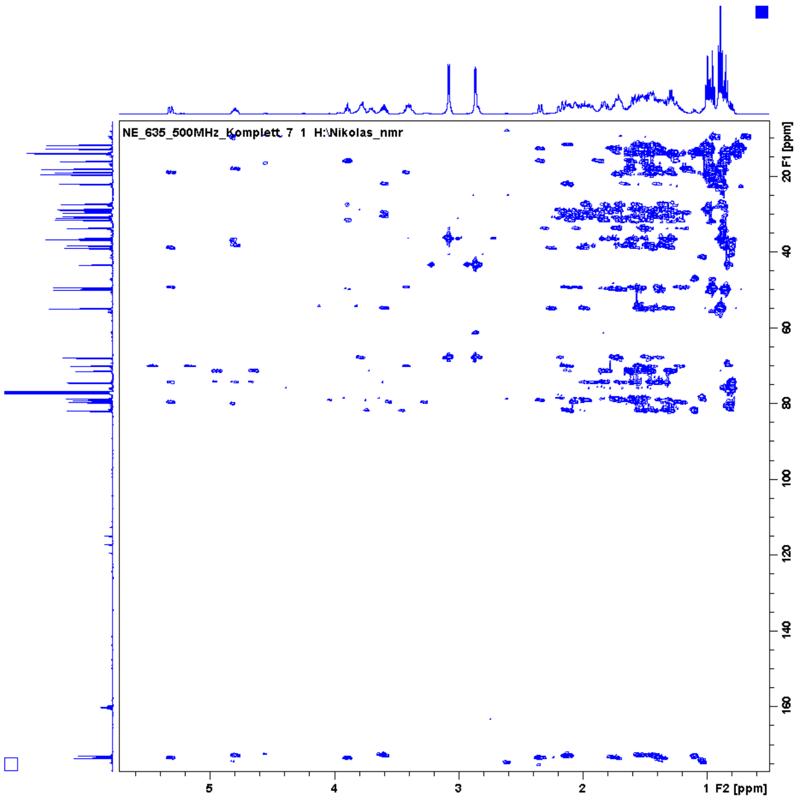

Fig. S'7. HMBC spectrum (CDCl3) of Pamamycin-635 G

Table S‘3. NMR data (700 MHz, $CDCl_3$) for Pamamycin-663 A

	δ_C m	δ_{CH} m (J in Hz)
**1**	172.31 C	-------------------
**2**	55.31 CH	2.11 ddd (14.0, 10.0, 4.0)
**3**	81.34 CH	3.66 ddd (11.0, 10.0, 4.2)
**4**	30.90 CH_2	1.20 m and 1.94 m
**5**	27.51 CH_2	1.71 m and 1.94 m
**6**	76.19 CH	4.21 ddd (9.0, 5.3, 2.2)
**7**	37.51 CH	1.93 m
**8**	75.20 CH	4.91 d (11.0)
**9**	46.41 CH	1.53 m
**10**	79.72 CH	3.36 dt (10.0, 7.0)
**11**	29.18 CH_2	1.61 m and 2.06 m
**12**	30.99 CH_2	1.53 m and 2.09 m
**13**	78.64 CH	3.75 br s
**14**	33.81 CH_2	1.71 m and 2.21 m
**15**	68.13 CH	3.39 br t (11.0)
**16**	28.72 CH_2	1.46 m and 1.72 m
**17**	19.61 CH_2	1.38 m and 1.52 m
**18**	13.81 CH_3	1.00 t (7.0, 3H)
**1´**	173.07 C	-------------------
**2´**	50.12 CH_2	2.36 dt (11.5, 2.5)
**3´**	78.13 CH	3.83 ddd (9.2, 6.0, 2.5)
**4´**	27.85 CH_2	1.62 m and 1.81 m
**5´**	26.70 CH_2	1.64 m and 1.77 m
**6´**	76.25 CH	3.94 td (7.6, 2.8)
**7´**	36.85 CH	1.87 m
**8´**	75.77 CH	4.57 dt (11.0, 4.0)
**9´**	34.24 CH_2	1.55 m and 1.68 m
**10´**	16.79 CH_2	1.25 m and 1.34 m
**11´**	14.21 CH_3	0.88 t (7.5, 3H)
**2-Et**	22.22 CH_2	1.42 m and 1.56 m
	11.75 CH_3	0.90 t (7.5, 3H)
**7-Me**	10.06 CH_3	0.82 d (7.0, 3H)
**9-Et**	18.98 CH_2	1.18 m and 1.54 m
	13.86 CH_3	0.95 (3H)
**15-N(Me)$_2$**	35.97 CH_{3a}	2.86 d (5.0, 3H)
	43.59 CH_{3b}	3.10 d (5.0, 3H)
**2´-Et**	15.99 CH_2	1.37 m and 1.77 m
	12.70 CH_3	0.83 t (7.5, 3H)
**7´-Me**	9.34 CH_3	0.82 d (7.0, 3H)
**NH**	-------------	8.74 br s

Table S’4. HMBC Key correlations for Pamamycin-663 A

**Position**	**HMBC correlations**
H-2	C-1, C-3, C-4, C-2-Et
H-3	C-1, C-2, C-5, C-2-Et
H-6	C-7, C-8, -7-Me
H-7	C-5, C-6, C-8, C-9, C-7-Me
H-8	C-6, C-7, C-9, C-10, C-1´, C-7-CH_3, C-9-Et
H-10	C-8, C-9, C-9- C-2-Et
H-13	C-15
H-18	C-16, C-17
H-2´	C-1´, C-3´, C-2´-Et
H-3´	C-1´, C-5´, C-2´-Et
H-6´	C-7´, C-8´, 7´-Me
H-7´	C-8´, C-9´, 7´-Me
H-8´	C-1, C-6´, C-7´, C-9´, C-10´, 7´-Me
H-11´	C-9´, C-10´
CH_3-2 (from Et)	C-2
CH_3-7	C-7
CH_3-9 (from Et)	C-9
CH_3-2´ (from Et)	C-2´
CH_3-7´	C-7´
CH_{3a}-N	C-15, 15-N-CH_{3b}
CH_{3b}-N	C-15, 15-N-CH_{3a}

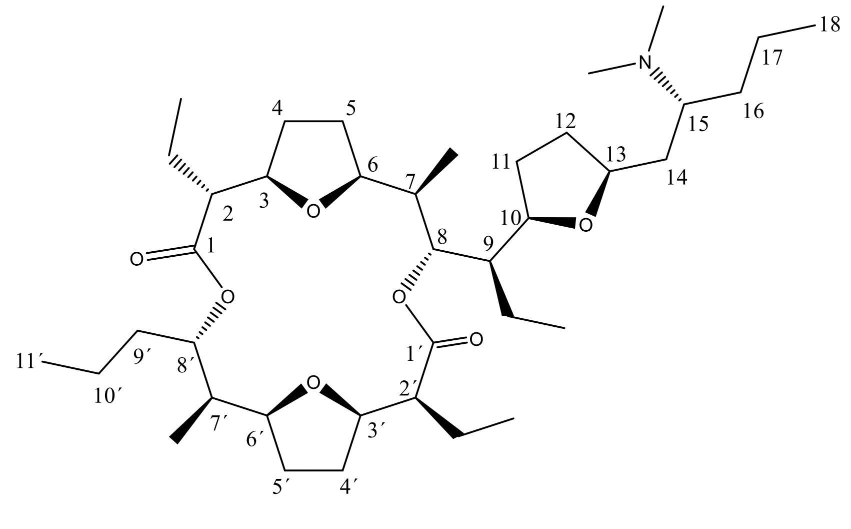

Fig. S’8. Structure of Pamamycin-663 A

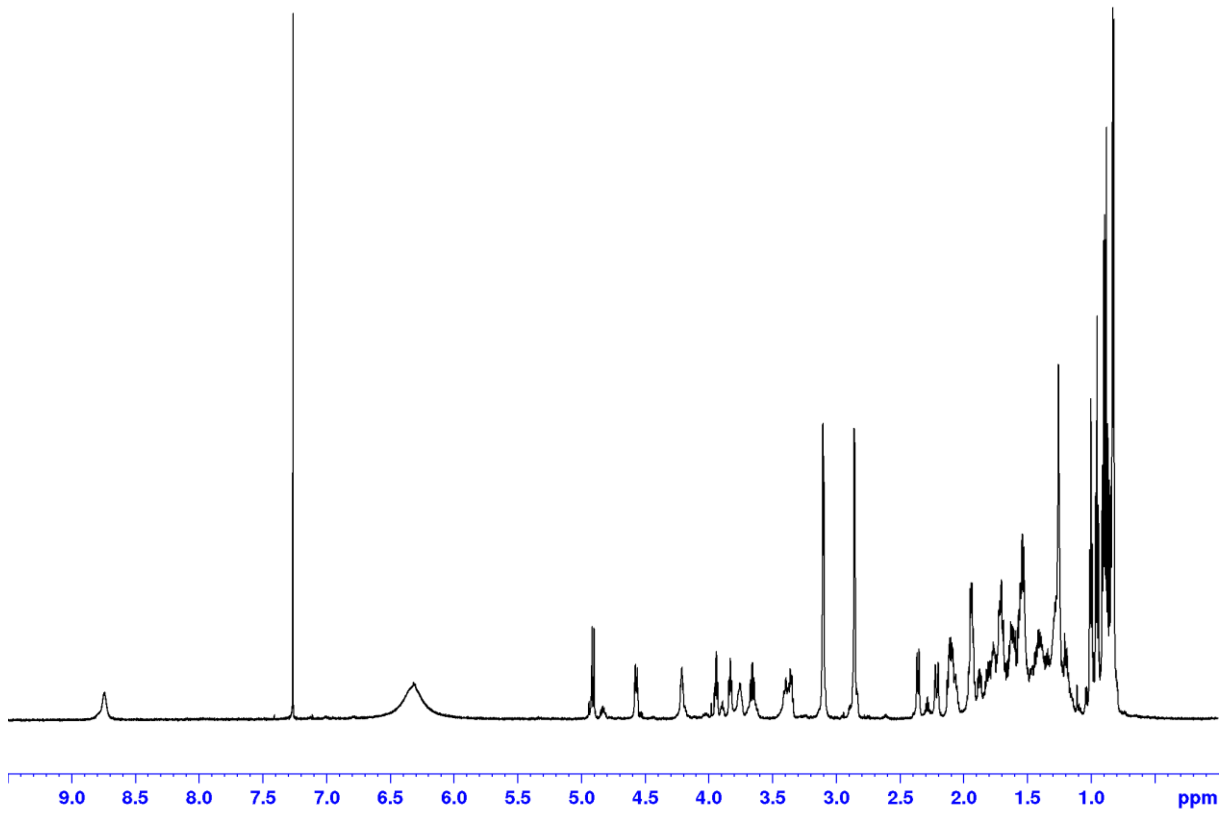

Fig. S’9. ^{1}H NMR spectrum (700 MHz, $CDCl_3$) of Pamamycin-663 A

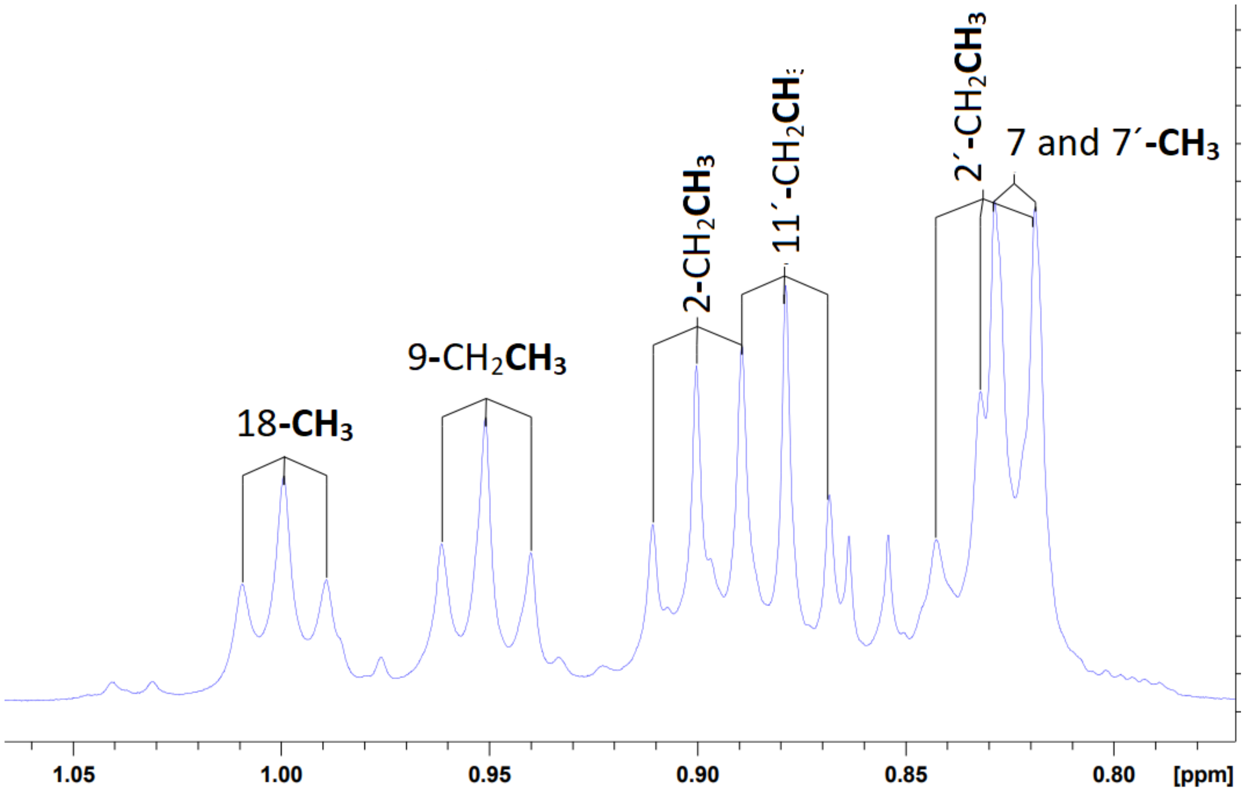

Fig. S’10. ^{1}H NMR spectrum (700 MHz, $CDCl_3$) of Pamamycin-663 A, Methyl region

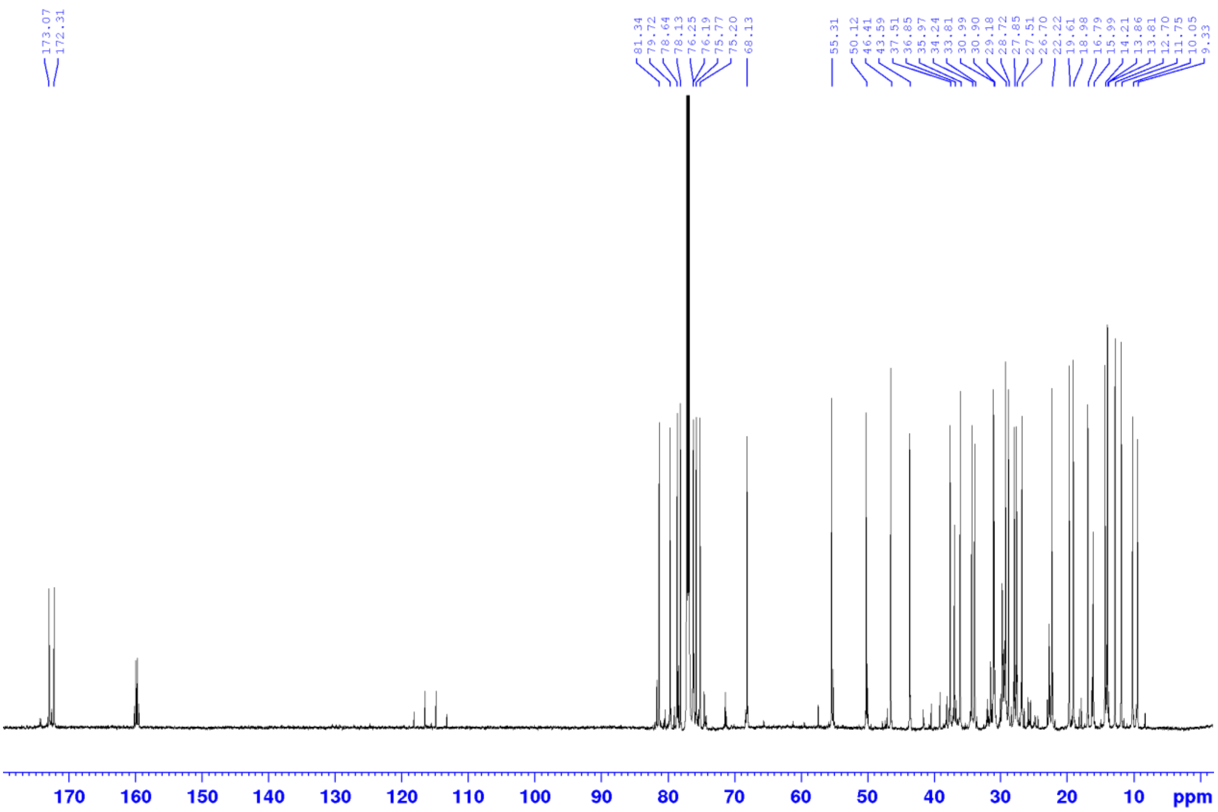

Fig. S'11. ^{13}C NMR spectrum (175 MHz, $CDCl_3$) of Pamamycin-663 A

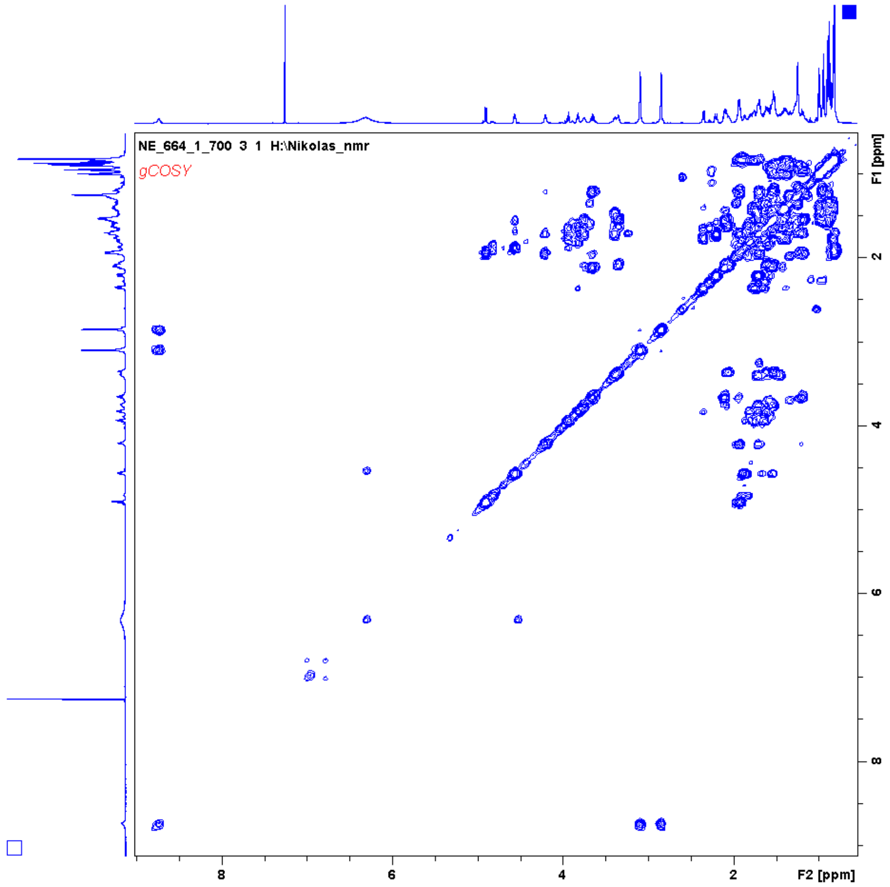

Fig. S'12. ^{1}H-^{1}H COSY spectrum (CDCl3) of Pamamycin-663 A

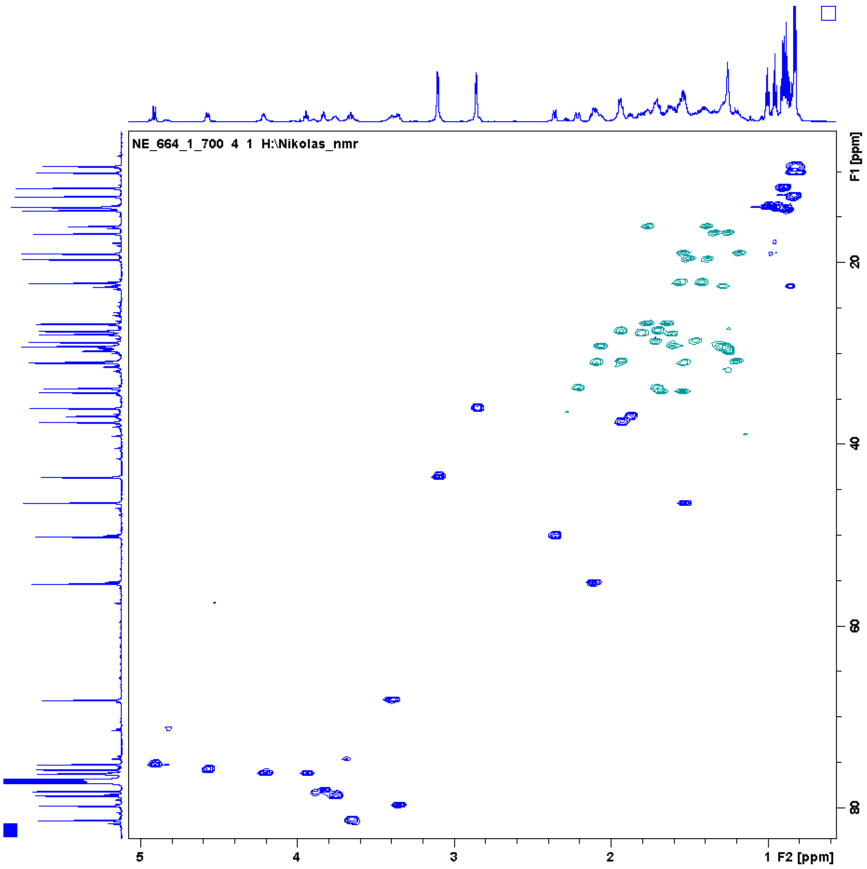

Fig. S’13. Edited HSQC spectrum (CDCl3) of Pamamycin-663 A

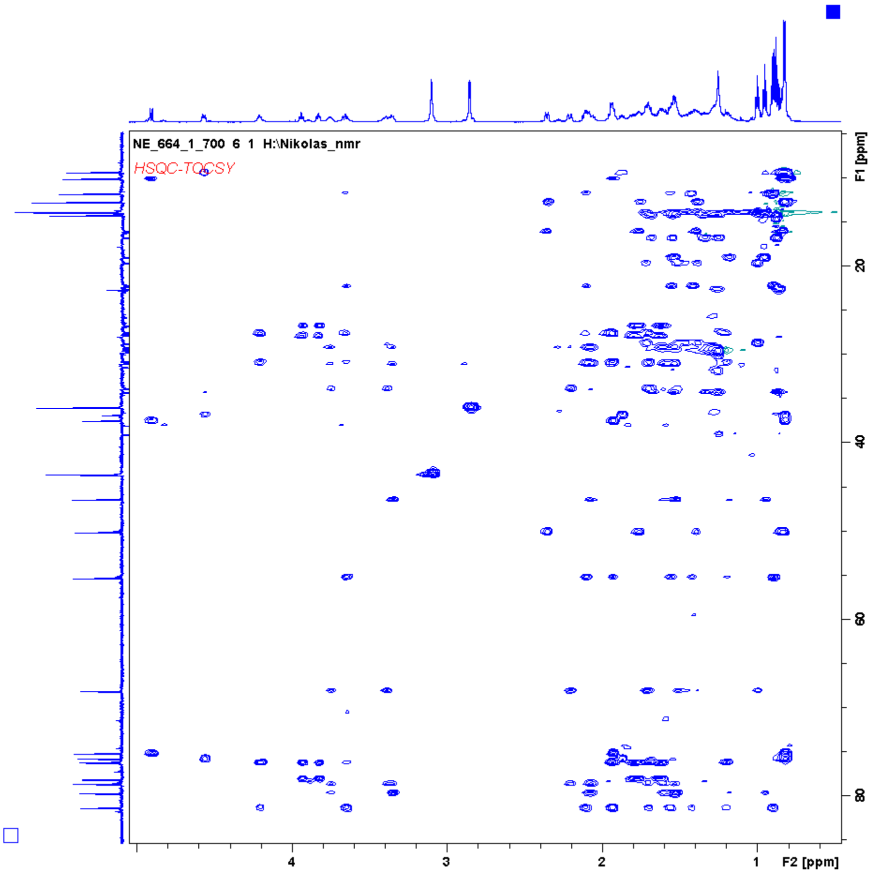

Fig. S’14. HSQC-TOCSY spectrum (CDCl3) of Pamamycin-663 A

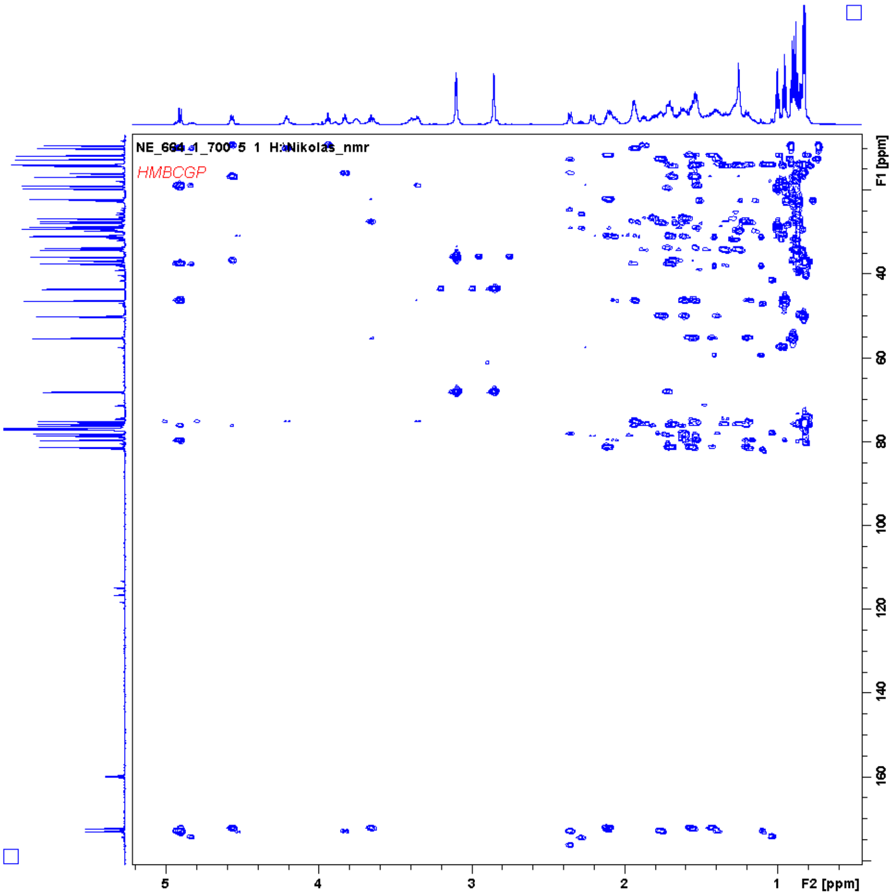

Fig. S’15. HMBC spectrum (CDCl3) of Pamamycin-663 A

Table S'5. NMR data (700 MHz, $CDCl_3$) of Homopamamycin-677 A

	δ_C m	δ_{CH} m (J in Hz)
**1**	172.30 C	-------------------
**2**	55.28 CH	2.11 m
**3**	81.34 CH	3.65 td (11.0, 4.0)
**4**	30.99 CH_2	1.20 m and 1.94 m
**5**	27.51 CH_2	1.70 m and 1.94 m
**6**	76.15 CH	4.22 m
**7**	37.46 CH	1.93 m
**8**	75.15 CH	4.92 d (11.0)
**9**	46.34 CH	1.53 m
**10**	79.65 CH	3.34 m
**11**	29.14 CH_2	1.61 m and 2.07 m
**12**	30.94 CH_2	1.53 m and 2.09 m
**13**	78.59 CH	3.76 m
**14**	33.71 CH_2	1.71 m and 2.21 m
**15**	68.05 CH	3.38 br t (11.0)
**16**	28.67 CH_2	1.46 m and 1.72 m
**17**	19.61 CH_2	1.38 m and 1.50 m
**18**	13.81 CH_3	0.99 t (7.0, 3H)
**1´**	173.04 C	-------------------
**2´**	50.07 CH_2	2.35 dt (11.5, 2.5)
**3´**	78.12 CH	3.82 td (6.5, 2.5)
**4´**	27.83 CH_2	1.61 m and 1.81 m
**5´**	26.66 CH_2	1.65 m and 1.77 m
**6´**	76.22 CH	3.91 td (7.6, 2.8)
**7´**	36.54 CH	1.89 m
**8´**	75.84 CH	4.59 dt (11.0, 4.0)
**9´**	34.12 CH_2	1.50 m and 1.79 m
**10´**	25.34 CH_2	1.25 m and 1.34 m
**11´**	22.89 CH_2	1.28 m (2H)
**12´**	14.01 CH_3	0.87 t (7.5, 3H)
**2-Et**	22.25 CH_2	1.43 m and 1.57 m
	11.77 CH_3	0.90 t (7.5, 3H)
**7-Me**	10.03 CH_3	0.82 d (7.0, 3H)
**9-Et**	18.98 CH_2	1.18 m and 1.54 m
	13.91 CH_3	0.95 t (7.5, 3H)
**15-N(Me)$_2$**	35.87 CH_{3a}	2.84 m
	43.50 CH_{3b}	3.09 m
**2´-Et**	15.99 CH_2	1.39 m and 1.77 m
	12.65 CH_3	0.83 t
**7´-Me**	9.27 CH_3	0.82 m
**NH**	--------------	8.74 br s

Table S'6. HMBC Key correlations for Homopamamycin-677 A

**Position**	**HMBC correlations**
H-2	C-1, C-3, C-2-Et
H-3	C-1
H-6	C-7-Me
H-8	C-7, C-9, C-10, C-1´, C-7-Me, C-9-Et
H-10	C-8, C-9, C-9-Et
H-13	C-15
H-18	C-16, C-17
H-3´	C-1´, C-2´-Et
H-6´	C-7´-Me
H-8´	C-1, C-6´, C-7´, C-7´-Me
CH_3-2 (from Et)	C-2
CH_3-7	C-7
CH_3-9 (from Et)	C-9
CH_3-2´ (from Et)	C-2´
CH_3-7´	C-7´
CH_{3a}-N	C-15, 15-N-CH_{3b}
CH_{3b}-N	C-15, 15-N-CH_{3a}

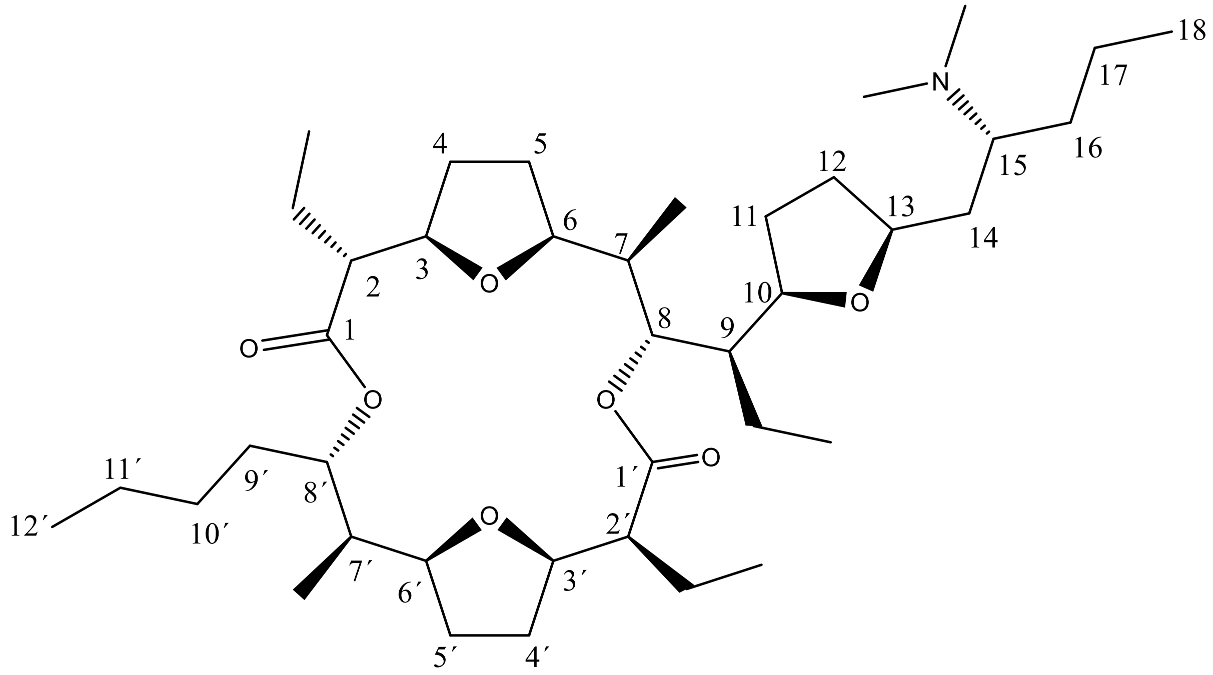

Fig. S'16. Structure of Homopamamycin-677 A

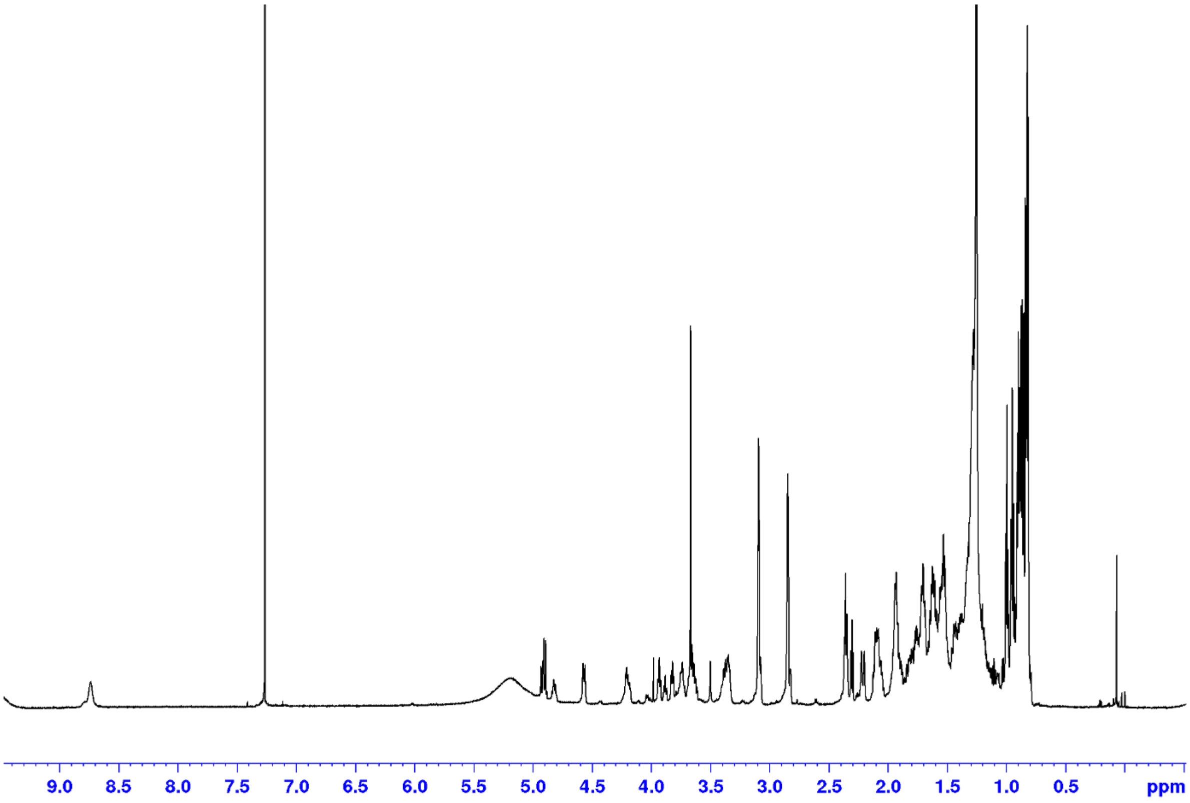

Fig. S'17. ¹H NMR spectrum (700 MHz, $CDCl_3$) of Homopamamycin-677 A

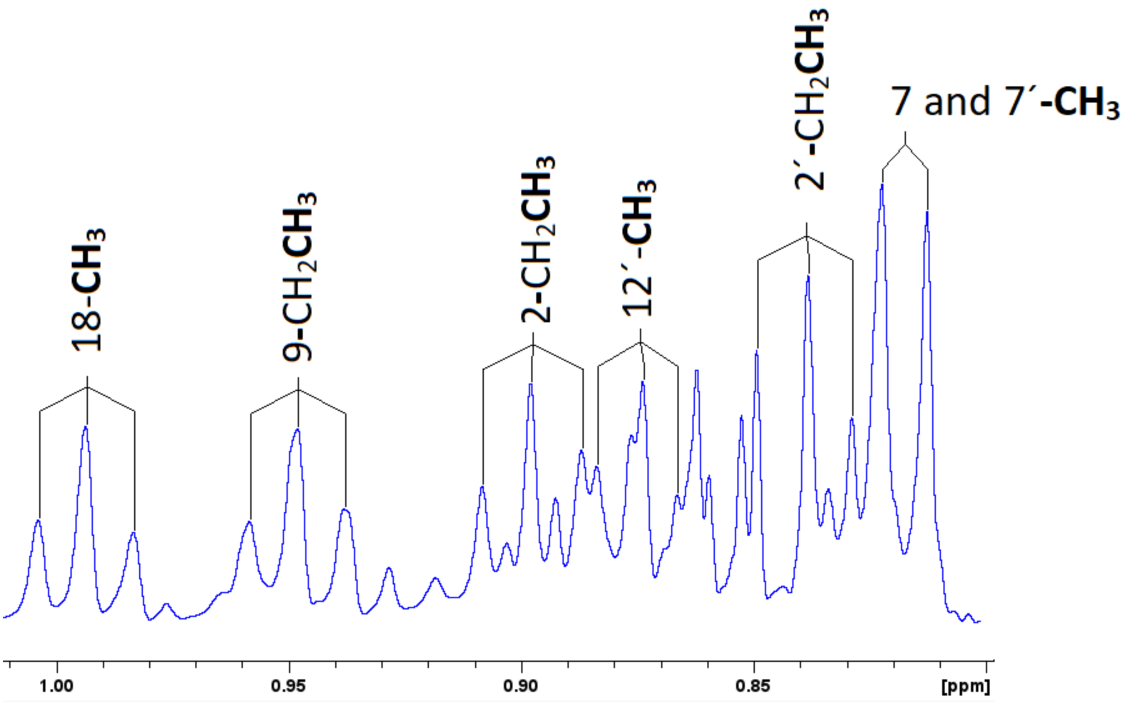

Fig. S'18. ¹H NMR spectrum (700 MHz, $CDCl_3$) of Homopamamycin-677 A, Methyl region

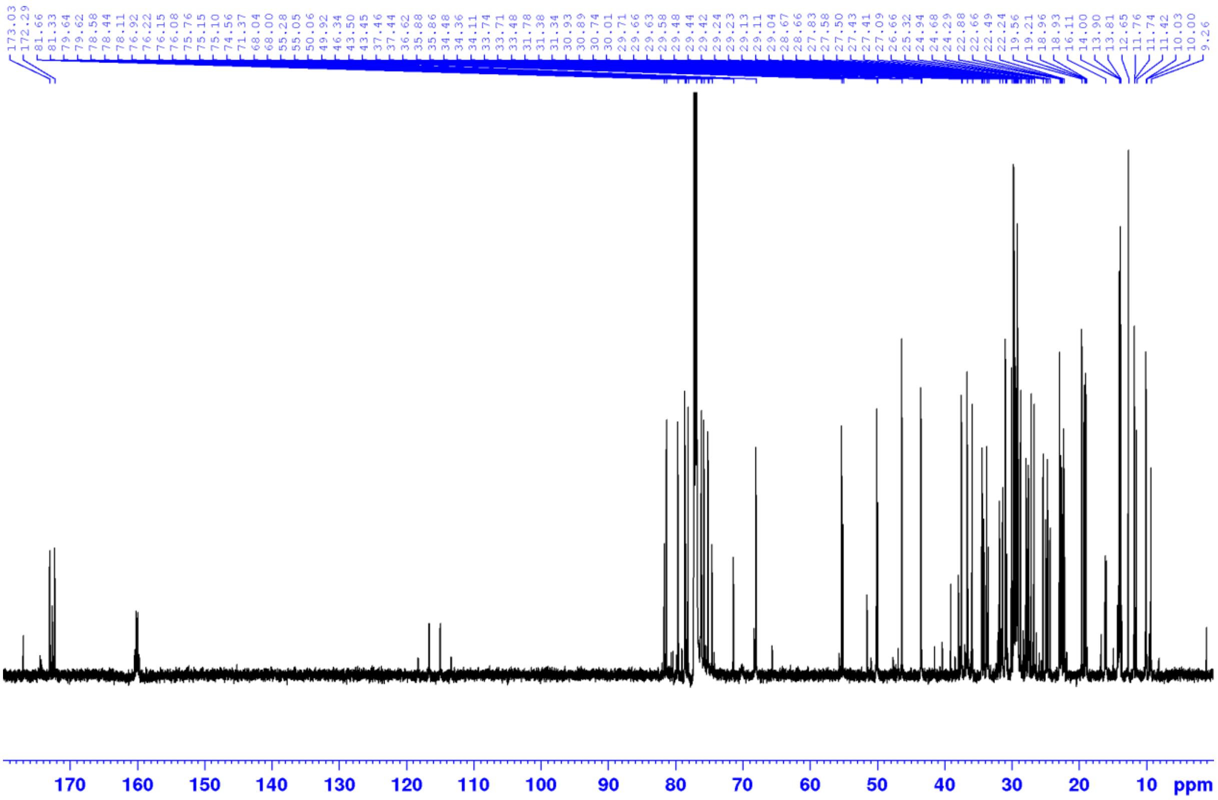

Fig. S’19. ^{13}C NMR spectrum (175 MHz, $CDCl_3$) of Homopamamycin-677 A

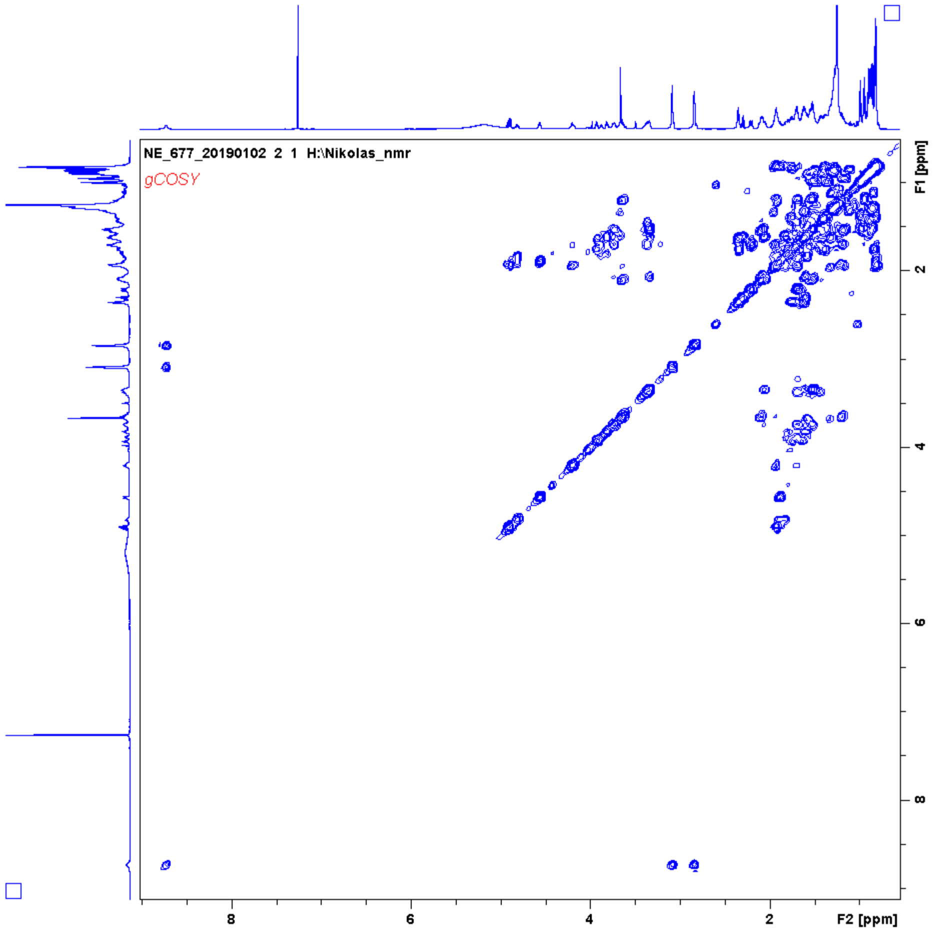

Fig. S’20. ^{1}H-^{1}HCOSY spectrum (CDCl3) of Homopamamycin-677 A

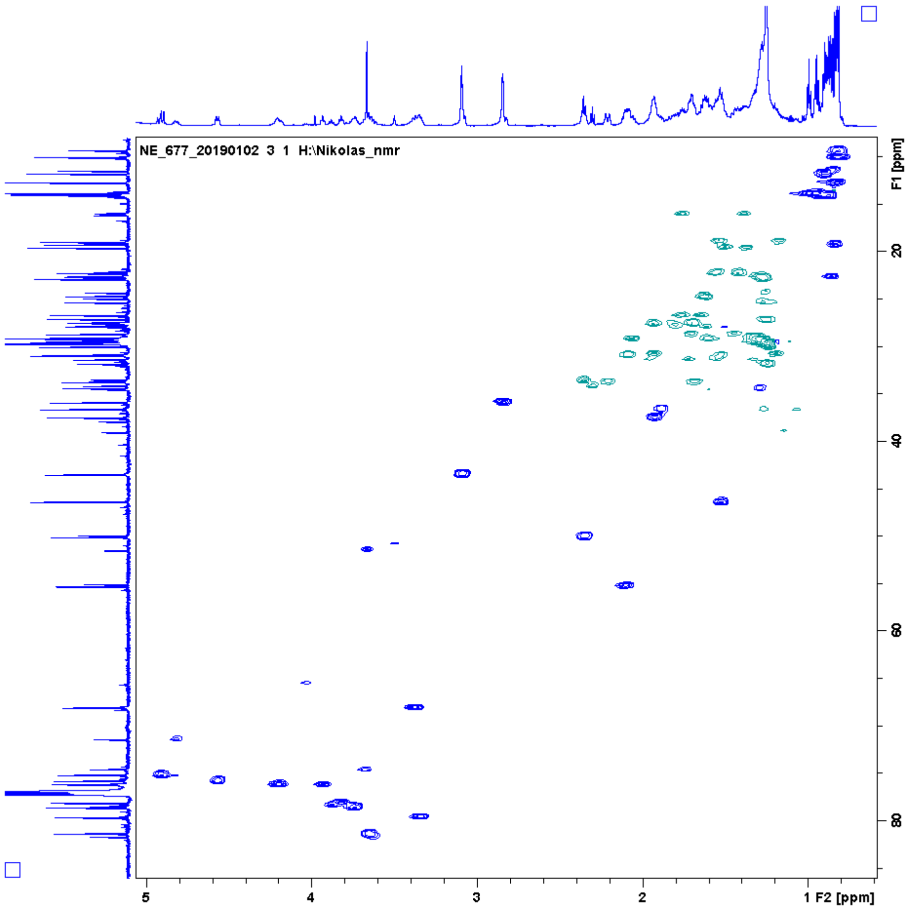

Fig. S’21. Edited HSQC spectrum (CDCl3) of Homopamamycin-677 A

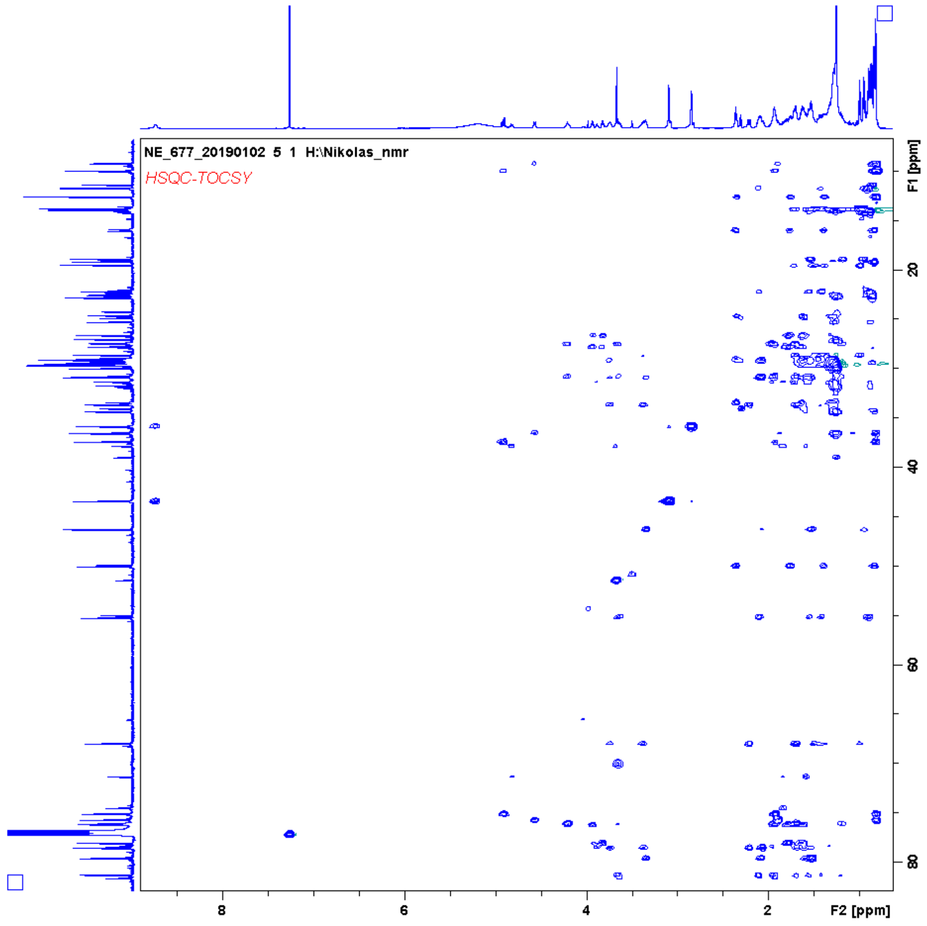

Fig. S’22. HSQC-TOCSY spectrum (CDCl3) of Homopamamycin-677 A

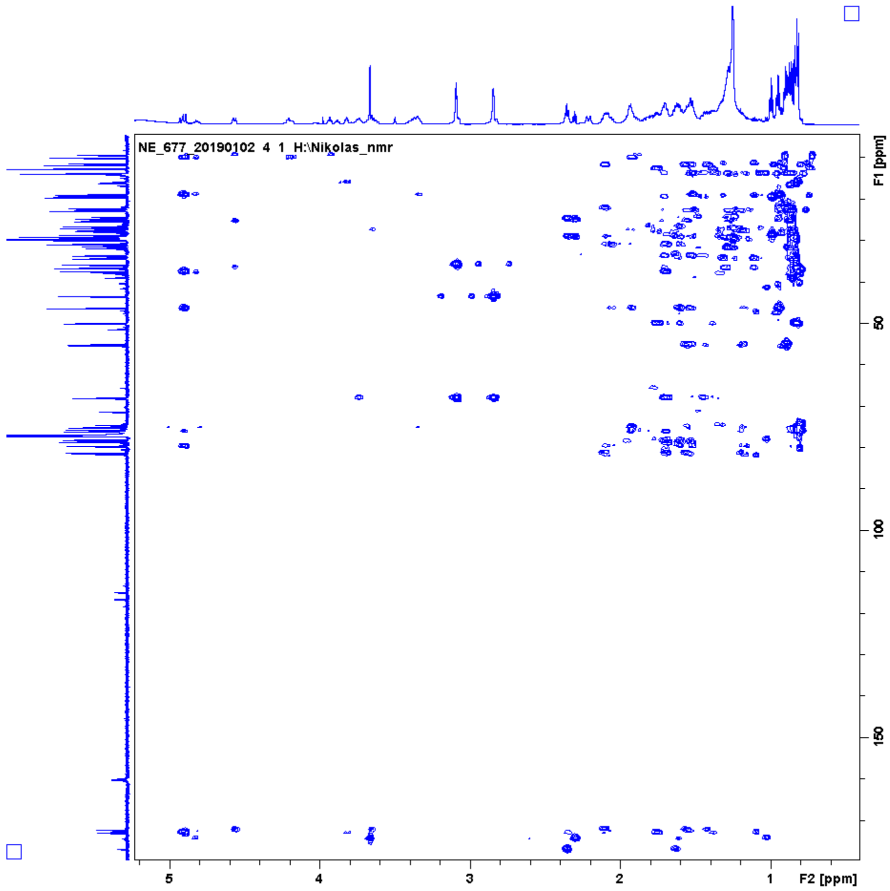

Fig. S’23. HMBC spectrum (CDCl3) of Homopamamycin-677 A

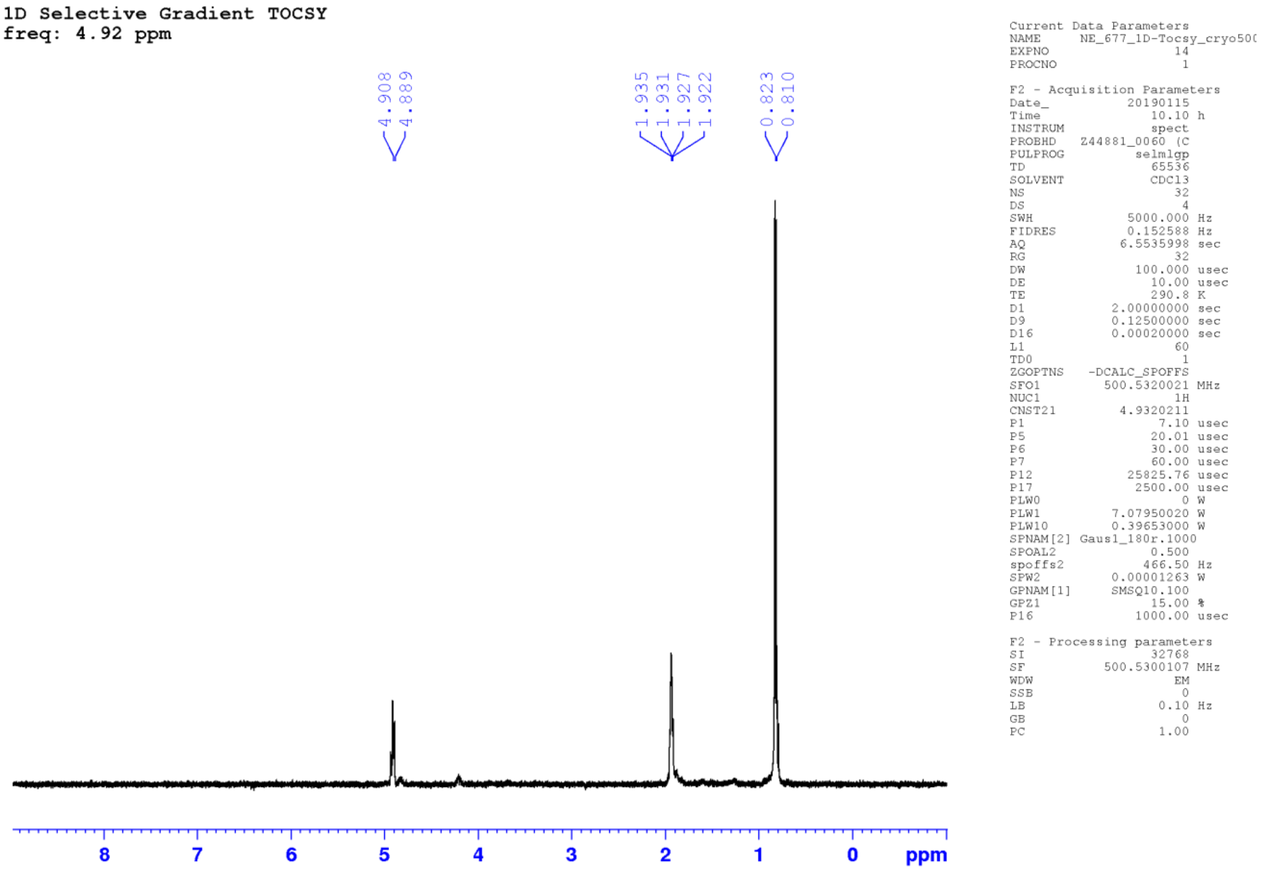

Fig. S’24. Sel. 1D TOCSY of Homopamamycin-677 A, excitation at 4.92 ppm

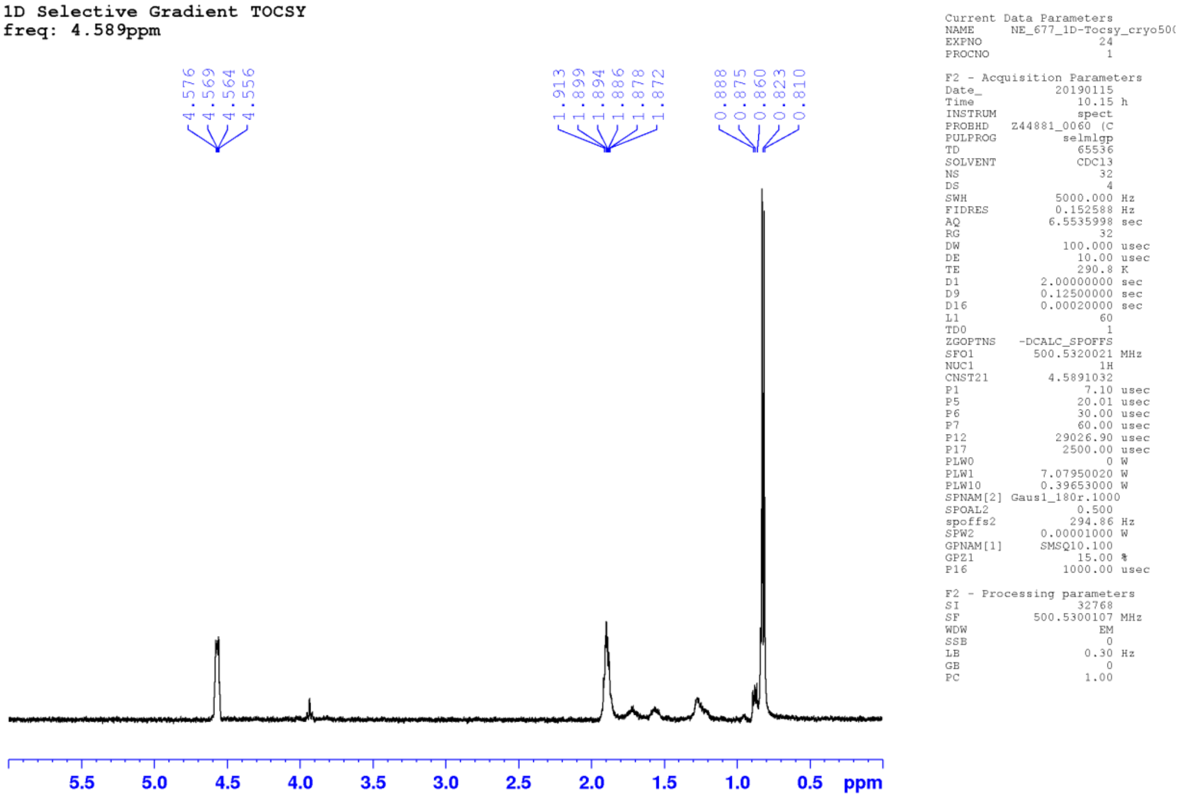

Fig. S'25. Sel. 1D TOCSY of Homopamamycin-677 A, excitation at 4.59 ppm

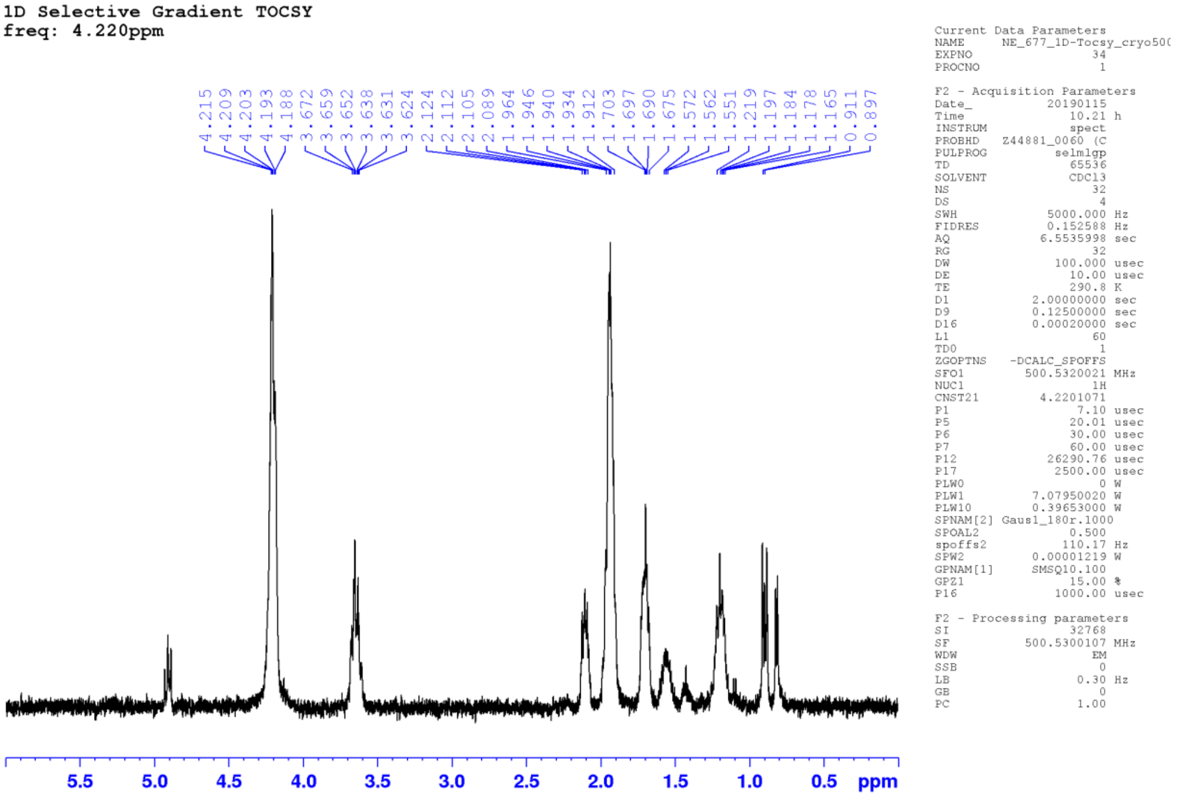

Fig. S'26. Sel. 1D TOCSY of Homopamamycin-677 A, excitation at 4.22 ppm

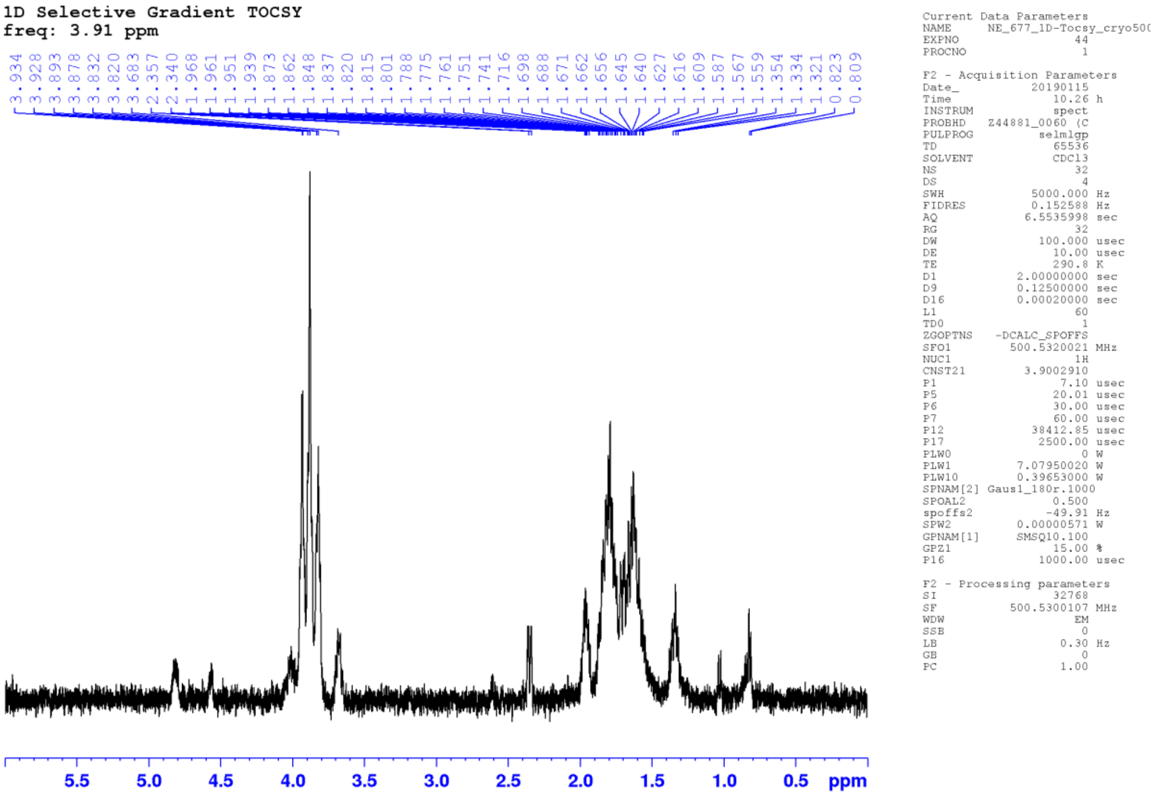

Fig. S'27. Sel. 1D TOCSY of Homopamamycin-677 A, excitation at 3.91 ppm

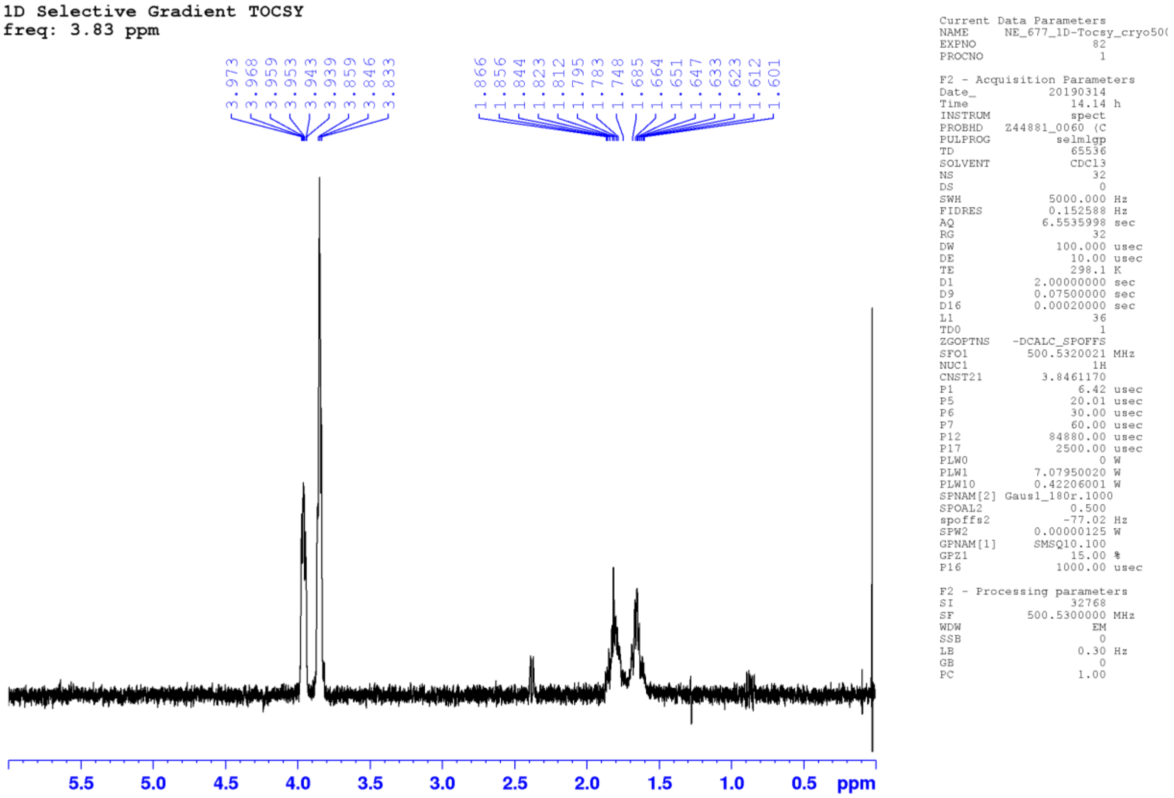

Fig. S'28. Sel. 1D TOCSY of Homopamamycin-677 A, excitation at 3.83 ppm

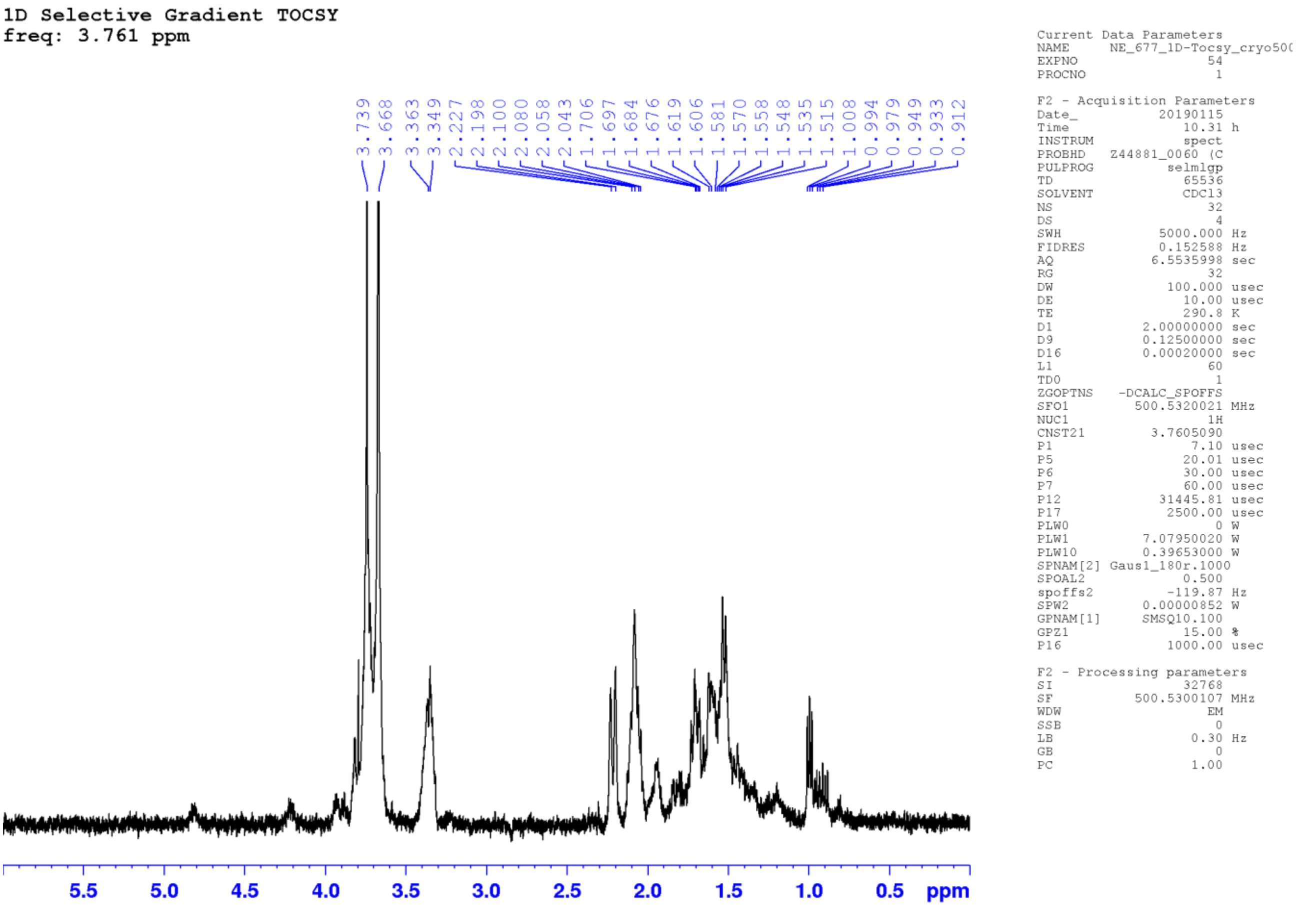

Fig. S'29. Sel. 1D TOCSY of Homopamamycin-677 A, excitation at 3.76 ppm

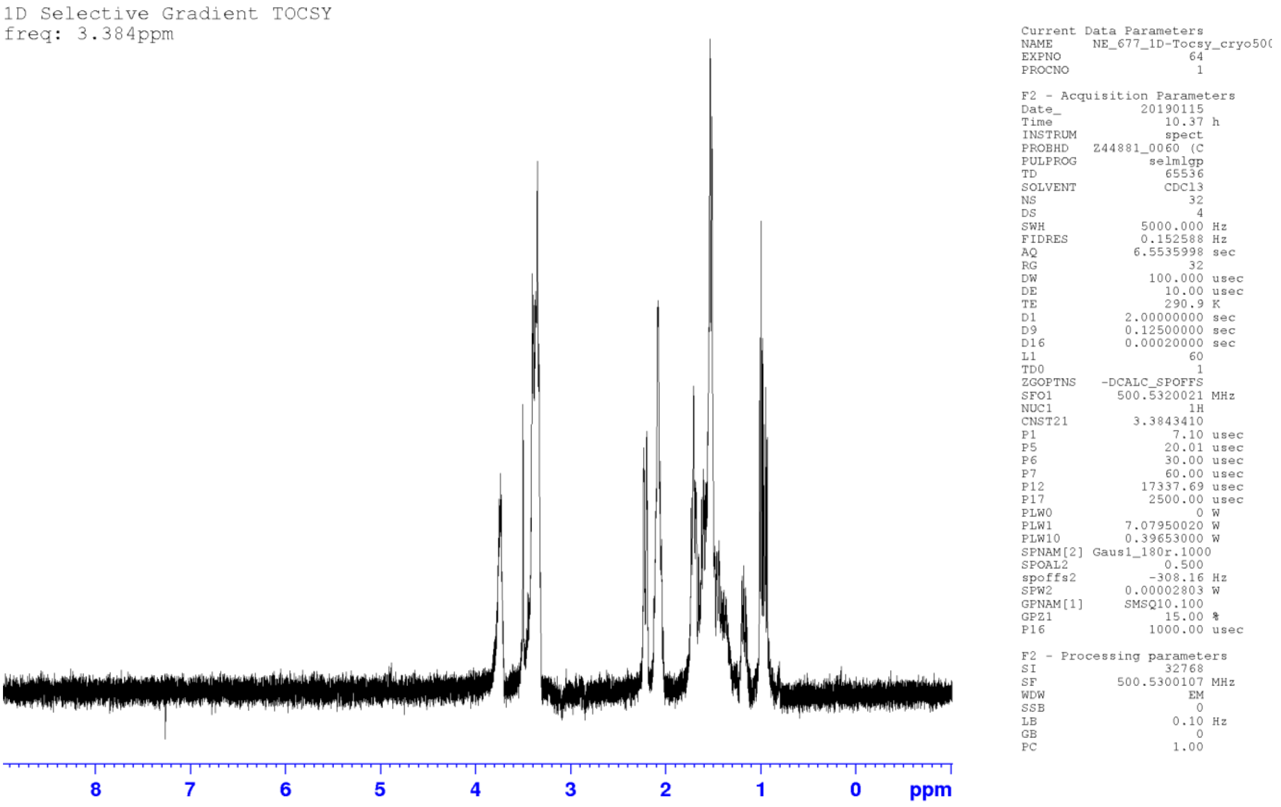

Fig. S'30. Sel. 1D TOCSY of Homopamamycin-677 A, excitation at 3.38 ppm

References

1. Flett, F., V. Mersinias, and C.P. Smith, *High efficiency intergeneric conjugal transfer of plasmid DNA from Escherichia coli to methyl DNA-restricting streptomycetes.* FEMS microbiology letters, 1997. **155**(2): p. 223-229.
2. Fu, J., et al., *Full-length RecE enhances linear-linear homologous recombination and facilitates direct cloning for bioprospecting.* Nature biotechnology, 2012. **30**(5): p. 440-446.
3. Fu, J., et al., *Efficient transfer of two large secondary metabolite pathway gene clusters into heterologous hosts by transposition.* Nucleic acids research, 2008. **36**(17): p. e113-e113.
4. Chater, K.F. and L.C. Wilde, *Streptomyces albus G mutants defective in the SalGI restriction-modification system.* Microbiology, 1980. **116**(2): p. 323-334.
5. Myronovskyi, M., et al., *Generation of a cluster-free Streptomyces albus chassis strains for improved heterologous expression of secondary metabolite clusters.* Metabolic engineering, 2018. **49**: p. 316-324.
6. Siegl, T., et al., *Design, construction and characterisation of a synthetic promoter library for fine-tuned gene expression in actinomycetes.* Metabolic engineering, 2013. **19**.
7. Herrmann, S., et al., *Site-specific recombination strategies for engineering actinomycete genomes.* Applied and environmental microbiology, 2012. **78**(6): p. 1804-1812.
8. Fedoryshyn, M., et al., *Functional expression of the Cre recombinase in actinomycetes.* Appl Microbiol Biotechnol, 2008. **78**(6): p. 1065-70.
9. Rebets, Y., et al., *Insights into the Pamamycin Biosynthesis.* Angewandte Chemie International Edition, 2014. **54**.
10. Myronovskyi, M., et al., *β-Glucuronidase as a sensitive and versatile reporter in actinomycetes.* Applied and environmental microbiology, 2011. **77**(15): p. 5370-5383.
